# An analytical and experimental study of the energy transition discourse on YouTube

**DOI:** 10.1371/journal.pone.0352691

**Published:** 2026-07-15

**Authors:** Aleix Bassolas, Piero Birello, Julian Vicens

**Affiliations:** 1 Big Data and Data Science Unit, Eurecat, Centre Tecnològic de Catalunya, Barcelona, Spain; 2 Department of Network and Data Science, Central European University, Vienna, Austria; Dong-A University College of Business Administration, KOREA, REPUBLIC OF

## Abstract

Energy production and management face significant political, economic, and environmental challenges. The rise in information consumption through social media can, under certain conditions, reduce the visibility and uptake of reliable knowledge, particularly in environments characterised by high information overload, algorithmic amplification, and low media literacy. This study examines the ideas discussed in the energy transition content on YouTube, assesses the most effective methods of communicating knowledge and information, and identifies the most engaged audiences. We examine videos related to the subject, analysing the themes discussed, the language used, and the emotions conveyed on YouTube, linking language formality to user engagement. To test the relationship experimentally, original content was uploaded to YouTube through two mirror channels containing the same material but using different levels of language formality. We conducted a systematic statistical analysis of engagement data collected from YouTube and the Google Ads platform. The YouTube engagement suggests that the conversational channel reaches a broader audience, although retention across video segments varies. While user retention was higher in the early segments for the conversational content, a higher retention in the mid to late sections of academic videos was found. Data from the Google Ads platform provided a deeper understanding of engagement across user profiles. Younger individuals and women show greater engagement regardless of language style, although women demonstrate relatively greater interest in the academic content.

## Introduction

Modern society depends fundamentally on energy, which sustains healthcare, food production, education, and economic development, and has long been considered the foundation of civilizational progress [1,2]. In recent years, global energy consumption has risen due to population growth and increasing demand from emerging economies [3]. Nevertheless, the increase in energy production comes with negative externalities, including environmental degradation and the depletion of natural resources [4–7]. Therefore, transitioning to a more sustainable energy system that reduces emissions, increases the share of renewable energy, and curtails overall energy consumption is crucial [8]. However, beyond technological and economic challenges, the success of this transition increasingly depends on how information is communicated and perceived by the public.

The energy transition faces several challenges, including technological [9], political [10], and cultural [11]. While renewable energy sources have seen increasing adoption worldwide, their integration with existing production systems and technologies remains a significant challenge [12]. On the political front, recent conflicts have disrupted energy resource availability and influenced policy shifts [13].

Furthermore, the energy transition is deeply intertwined with human behaviour, decision-making, and public discourse [14,15]. Strategic frameworks for prioritising renewable energy are essential for achieving sustainable development goals, yet their success ultimately depends on public engagement and acceptance [16]. This engagement is shaped by complex human factors, including how individuals process information and make decisions within energy markets, a process influenced by cognitive mechanisms and communication frames [17]. For instance, the adoption of related technologies, such as new energy vehicles, is significantly affected by perceived behavioural control and social influence [18]. In the digital age, social media platforms, particularly YouTube as a major source of long-form informational content, have become critical arenas where these perceptions are formed and contested, highlighting the need for effective and reliable scientific communication. However, the increasing consumption of information driven by social media has made it more challenging to gain visibility and actively engage citizens [19,20]. In this context, researchers have explored how content features shape user interest and engagement.

Communication represents a critical bottleneck in the energy transition. Analysing this digital discourse therefore requires the systematic characterisation of language use, emotional tone, and thematic alignment. Language analysis initially relied on manual annotation to assess opinions and themes [21,22], which progressively evolved into the use of closed-lexicon approaches based on dictionaries created by domain experts to identify specific word categories [23,24]. These developments gave rise to a wide range of methods addressing distinct dimensions of language analysis, including sentiment evaluation [25], automated text categorisation [26], and the detection of stylistic formality [27]. However, these methods face limitations in scalability and in capturing contextual and semantic nuances, which were addressed through the increase of computing power and the development of probabilistic and machine learning methods [28,29]. This transition led to significant advances in text indexing through co-occurrence patterns [30] and more robust topic analysis based on word distributions [31,32].

More recently, transformer-based architectures, such as BERT, have enabled improved contextualised representations of textual meaning by capturing relationships between words beyond simple word frequencies [33–35]. Nowadays, topic analysis is essential to unveil thematic discussions in social media related to political events [36,37], advertising strategies [38], public health [39,40], and scientific topics such as climate change [41]. Sentiment analysis reveals crucial insights related to public perceptions and opinions on political and social subjects [42,43], as well as global events and crises [44–46]. Furthermore, the analysis of language formality allows for the comparison of formality across scientific publications and online platforms such as Reddit, Twitter, and Wikipedia [47,48]. Together, these approaches enable large-scale examination of online discourse and highlight the need to understand how linguistic features shape information dissemination, informing the present study, which investigates energy transition content on YouTube in terms of topics, sentiment, and language formality.

Building on these methodological advances, previous research has investigated drivers of digital engagement. On Twitter (now X), studies have shown that factors such as the number of hashtags, URLs, and followers positively impact content reach and engagement [49,50]. Similarly, on YouTube, the number of subscribers and the upload date play a key role in predicting future views [51]. Content type also influences the spread of information [52–54], with videos in the music and entertainment categories tending to generate higher user engagement [51]. Beyond content type, the emotions and sentiments expressed in posts influence both diffusion and engagement. Content with strong emotional appeal, whether positive or negative, tends to attract more interest [55]. Some studies suggest that while negative sentiment drives faster reactions, positive sentiment promotes longer-term content diffusion [56]. Additionally, content with a negative sentiment has been associated with increased views and comment activity on YouTube [57]. In fact, YouTube has become a widely used platform for educational content, offering longer videos than other platforms, which allows for the discussion of more complex subjects [58]. Despite extensive research on content features and engagement, particularly in the context of scientific communication on social media [59,60], the role of linguistic formality remains insufficiently understood, with existing findings being limited and inconclusive [61,62].

This study contributes by combining large-scale observational analysis with a controlled experiment with two objectives: to understand how information on the energy transition is conveyed on YouTube and whether language features, specifically formality, influence engagement. As social media platforms become more influential in disseminating information on public policy and science to the general public, understanding the role of language use on engagement has become increasingly important [63,64]. While most studies focus on observational data [55], there is a limited implementation of ad hoc experiments on online platforms. Such experiments provide a different perspective on the impact of language, communication, and platform design on engagement.

To address this gap, we analyse energy transition content on YouTube by examining its thematic structure and linguistic features, focusing on emotional tone and formality, and conduct an experiment to assess the influence of language on audience engagement and profiles. In the first part of this study, we collected videos related to energy transition keywords and analysed the topics covered, the sentiments expressed, and their correlation with engagement metrics (views, likes, and comments). In the second part, 20 original science communication videos were produced and published on two identical channels that differed in language formality. We then conducted systematic comparisons and statistical tests to evaluate the effect of language formality on content dissemination, focusing on the engagement and user retention. Finally, we characterise audience segments based on their differential engagement patterns, providing insights into how communication style interacts with audience heterogeneity in shaping the reach and impact of energy transition content.

## Materials and methods

### Data

We conducted two complementary studies on energy transition content on YouTube: one focused on analysing existing videos, and the other on examining original content produced and published on the platform. Data for both studies were collected using the YouTube Data API [65], following the official documentation and adhering to the platform’s quota limitations. To capture different aspects of public discourse on energy management and the energy transition, we selected 13 key concepts: energy economy, energy supply and demand, geopolitics of energy, electricity market, renewable energy, energy and mobility, energy savings, energy storage, energy transition, energy efficiency, decarbonisation, energy model, and energy resources. For each concept, we retrieved information about 200 Spanish videos. Data extraction was restricted to Spanish to ensure contextual consistency in the observational analysis and to maximise the availability of comparable content on the platform. The data was collected using the *Search* function [66] of the YouTube Data API, which allows content searches based on specific keywords. The search was performed using relevant keywords for each concept, specifying Spanish in the *relevanceLanguage* field. However, while this parameter prioritises a given language, it does not guarantee that all retrieved content is in the intended language. We have detected the language of the video descriptions and transcriptions, keeping only those in Spanish. Additionally, to ensure that the videos were meaningful to the subject, we ensured that the video descriptions included variations of the terms energy or electricity. The final dataset analysed has 2,108 videos distributed across the 13 concepts. Since the algorithm governing video retrieval is not publicly disclosed, we cannot provide further details on how videos are selected. We provide a detailed analysis of the dataset in S1 File.

The data from the channels created during this research was collected using two sources: the YouTube API and the Google Ads API. The data from YouTube was obtained through the Analytics and Reporting API [67], which provides access to daily and aggregated measures of engagement and retention to channel owners [68]. Engagement measures include the number of views, likes, and comments, and retention measures include the *audience watch ratio* and the *relative retention performance*. We collected data for the 20 challenges for which we published videos. The first 2 videos were used to assess the engagement on YouTube for content posted on newly created channels. The following 3 videos were used to test the Google Ads platform and adjust the campaign parameters (available in S1 File). Thus, the main analysis is centred on the last 15 challenges. Considering that each challenge has four different formats and there are two mirror channels, we analysed a total of 120 videos. Each challenge had two complete videos, which were longer, and two brief videos (See S8 Fig). The complete videos have a promoted and a non-promoted version, and the brief videos, both promoted, have standard and short formats with vertical orientation. The detailed data related to the Google Ads promotion campaigns is available through the Google Ads API [69]. Besides the number of visualisations and user interactions (clicks), the platform also provides the number of impressions. The metrics are also provided disaggregated by age, gender, and device. The data from Google Ads is at the campaign level, grouping the videos for each individual promotion. Thus, we have collected and analysed data on 15 challenges.

### Methods

**Fig 1:**
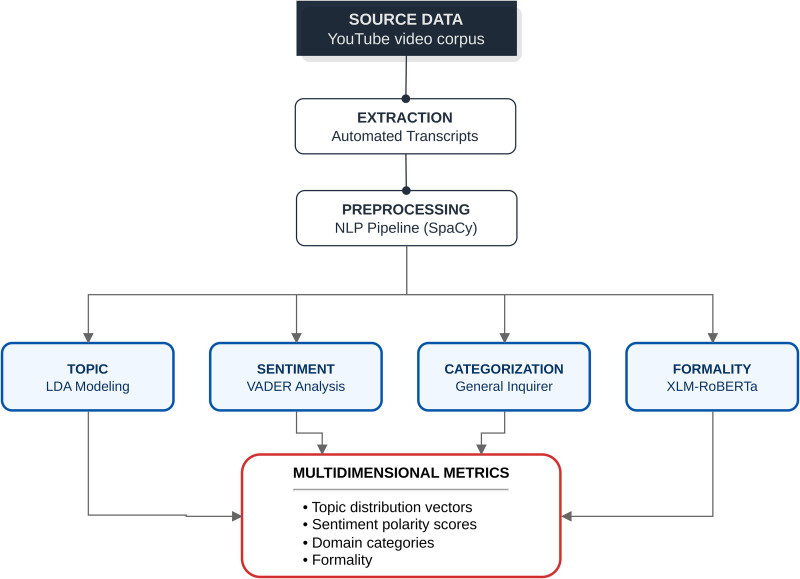
Computational workflow for YouTube transcript analysis. This pipeline details the transition from a raw video corpus to structured data. It concludes with the generation of multidimensional metrics, including topic distributions, polarity scores, formality scores and domain categories.

This study employs a multi-method natural language processing framework (Fig 1) to characterise the content and dissemination of energy transition discourse in online video transcripts. The analytical pipeline integrates complementary approaches to capture thematic structure, emotional tone, and linguistic formality, enabling a comprehensive assessment of how communication styles relate to audience engagement.

#### Topic modelling.

We apply topic modelling to identify latent semantic structures within a corpus of documents, in this case, video transcripts. We represent topics as groups of words that co-occur within documents and reflect shared semantic themes. To facilitate the extraction of meaningful topics, we have preprocessed the texts using the package Spacy [70], removing stopwords, pronouns, and verbs, keeping nouns, proper names, adjectives, and adverbs.

We employ Latent Dirichlet Allocation (LDA), a probabilistic model based on Bayesian methods, to detect topics in the corpus of video transcripts. The model infers topic distributions by assuming that each document is a mixture of topics and that each topic corresponds to a distribution of words learned through statistical inference [71,72].

#### Polarity calculation.

We compute sentiment polarity using the VADER model [25], a lexicon- and rule-based approach built on validated word classification dictionaries. The model assigns scores that capture the intensity of sentiment conveyed by words and, therefore, the content. It is specifically designed for social media text, where the content often contains informal language, slang, abbreviations, or emoticons. VADER outputs three normalised polarity scores (positive, neutral, negative) that sum to one.

#### General inquirer.

To complement polarity analysis, we employ the General Inquirer [73], a dictionary-based tool that classifies words into categories related to emotional, cognitive, and psychological processes. The specific categories used in this study are detailed in the Supplementary Information.

#### Formality calculation.

To quantify linguistic formality, we employ the XLM-Roberta-base [74] language model trained on the X-FORMAL [75] dataset and apply it to English-translated transcripts. The model receives text as input and returns complementary scores for formal and informal language. Because the model is designed for written text, it is sensitive to punctuation and capitalisation, which are typically absent in YouTube transcripts. To address this, we detect sentences within the transcripts, introduce punctuation to separate them, and restore sentence-initial capitalisation. We compute formality scores at the sentence level and then average them to obtain transcript-level measures.

## Results

### An analysis of the discussion on YouTube of the energy

We first conducted a statistical analysis of energy-related content within the Spanish YouTube community (S3 and S4 Figs). The results indicate that total views have the highest engagement values, followed by likes and comments. Comparing the engagement across the 13 concepts, the content related to the *renewable energy* has the highest number of views and comments. We also calculated the ratio between the average number of comments and views to assess the user interaction per view, where concepts with higher values are the *electricity market* and the *energy model*.

**Fig 2:**
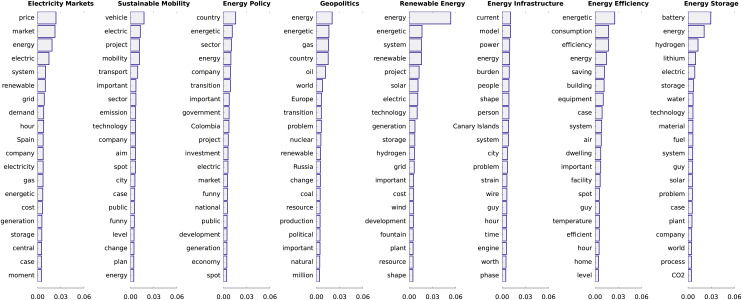
Importance of the words in the detected topics. Ranking and weight of the most important words by topic detected.

We have conducted an LDA topic modelling to identify the main themes discussed in YouTube videos and how the concepts are distinguished. The eight topics detected are depicted in Fig 2, where a weighted set of words represents each topic. Despite the concepts revolving around the same shared subject, the topics have distinctive words.

Each video was assigned a single topic based on the highest probability score from the topic modelling process. The eight identified topics reflect distinct thematic areas within the Spanish YouTube energy discourse:

- *Electricity markets* includes content related to the electricity market, energy models, supply and demand, and the energy economy. It captures discussions around the economic and regulatory aspects of energy systems.
- *Sustainable mobility* focuses on transportation-related terms such as vehicle and transport, highlighting the role of mobility in the energy transition.
- *Energy policy* encompasses videos discussing legislation, regulation, and government strategies, addressing how political frameworks shape the energy sector.
- *Geopolitics* features mentions of countries and energy resources like oil and gas, reflecting the international and strategic dimensions of energy.
- *Renewable energy* contains the largest number of videos, likely due to its broader and more accessible terminology with words such as electric, energy, solar, and wind. This topic covers interconnected themes like renewable energy, energy storage, and energy resources.
- *Energy infrastructure* relates to physical systems and projects supporting energy generation and distribution, including terms like grid, installation, and development.
- *Energy efficiency* groups content around terms such as energy savings, buildings, decarbonisation, and efficiency, highlighting efforts to reduce energy consumption and emissions.
- *Energy storage* includes videos focused on technologies and strategies for storing energy, featuring specific terminology and more technical discussions.

**Fig 3:**
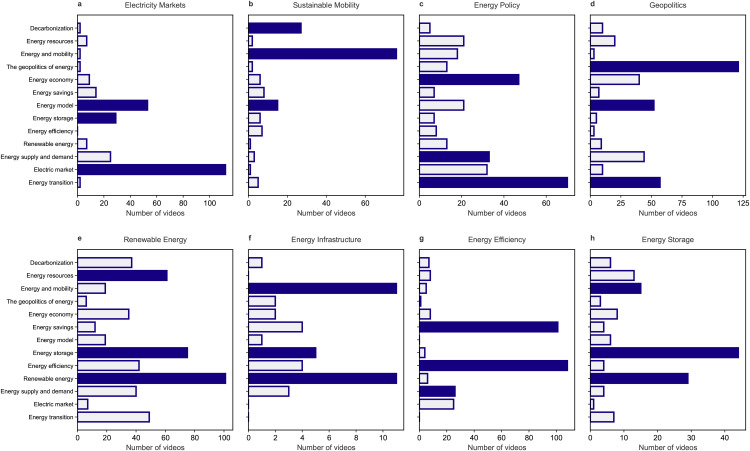
Distribution of concepts by topic. Number of videos per concept in each topic, where the highlighted bars in blue represent values above the 75th percentile, topics in the top 25%.

Topics with broader or more widely used vocabulary—such as *renewable energy*, *electricity markets*, and *energy efficiency—*tend to include a higher number of videos. In contrast, topics with more specialised or technical focus—such as *energy infrastructures*, *sustainable mobility* (including discussion on electric vehicles), and *energy storage* generally correspond to a smaller number of videos (Fig 3).

**Fig 4:**
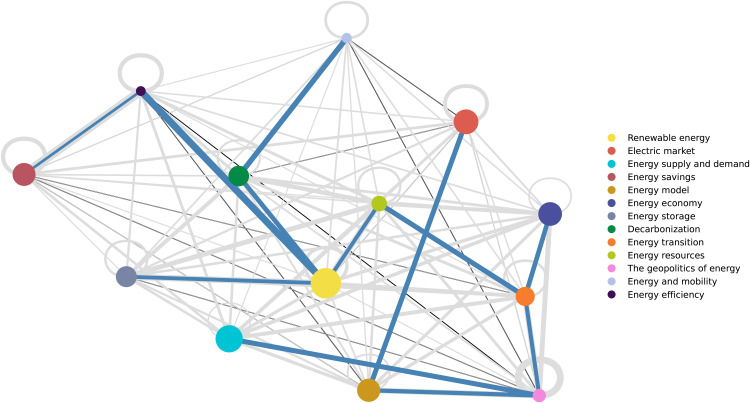
Concept network. Network between concepts based on the number of videos that share the attributed topic. The thickness of the links is determined by the number of concept videos assigned to the same topic, normalised by the number of possible pairs of videos from the two connected concepts. The links in blue correspond to the minimum spanning tree and the dot size to the concept outflow.

We constructed a network in which nodes represent the 13 original concepts, and links reflect videos from two concepts assigned to the same topic. To account for differences in concepts’ frequency, each link weight is normalised by the number of videos associated with the connected concepts. We show the structure of this network in Fig 4, which illustrates the relations between concepts. We have assessed the most connected pairs and triads of keywords by calculating the weighted keyword co-occurrence given by


$$\begin{array}{c} W_{ij}=\sum_{t\in T}w_{ti}\cdot w_{tj} \end{array}$$ (1)

for keyword pairs, and


$$\begin{array}{c} W_{ijk}=\sum_{t\in T}w_{ti}\cdot w_{tj}\cdot w_{tk} \end{array}$$ (2)

for keyword triads. We have focused on dyads and triads to facilitate interpretability, taking into account that they are already representative given the small network size. Where $w_{ti}$ is the number of times that keyword *i* appears in topic *t*. The most connected pairs are: (i) the *energy efficiency* and *energy savings* ($W_{ij}=11577$); and (ii) *t*he *renewable energy* and *energy storage* ($W_{ij}=9275$). The results suggest that videos on renewable energy are closely related to energy storage, as are the videos related to decreasing energy consumption. The most connected unique triads are: (i) the *renewable energy*, the *energy storage*, and the *energy resources* ($W_{ijk}=481188$); and (ii) the *energy transition*, the *geopolitics of energy*, and the *energy model* ($W_{ijk}=364356$). The concepts most isolated from the rest, computed as the ratio between the outflow (sum of outgoing weights excluding self-loops) and the total flow (sum of outgoing weights including self-loops), are the *geopolitics of energy* (with 0.83) and the *energy savings* (with 0.87). We have also computed the Minimum Spanning Tree (MST) to identify the connection backbone between topics since the network is fully connected. The concepts with highest betweenness centrality $b_{c}$ in the MST (blue edges in Fig 4) are the *renewable energy* ($b_{c}=0.65$), the *energy transition* ($b_{c}=0.59$) and the *energy resources* ($b_{c}=0.55$).

After extracting themes from our corpus of video transcripts, we conducted sentiment and content analysis to detect and quantify relevant semantic features. We analysed the polarity of the transcripts and classifications related to emotions, morality, semantic areas, and formality. We used the VADER model to quantify the polarity of the transcripts and compare the emotions conveyed.

**Fig 5:**
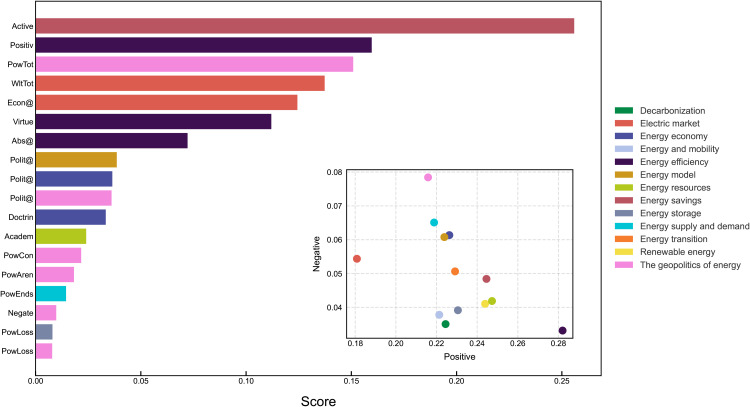
Analysis of the polar sentiment and semantic areas of the transcripts. Concept with the highest value for each semantic area. Semantic areas are shown in the vertical axes, and the colour indicates the keyword with the highest value. Only semantic areas where a keyword has a significantly larger value than the rest are shown. The inset shows the average negative emotions as a function of positive emotions for each concept.

Neutral sentiment predominates with values around 0.7 for a total of 1, implying that the discussion is carried out mostly neutrally without emotional connotations. Overall, positive sentiment exceeds negative sentiment, which only reaches 0.1 in one concept. We analysed the negative sentiment versus the positive sentiment in the inset of Fig 5. *Energy geopolitics* is the topic with the highest negative sentiment, followed by *energy supply and demand*, which suggests that the perception of the political and economic situation related to energy management is the most negative among the concepts studied. The main conclusion is that concepts assigned to topics with a higher presence of economic and political terms are more negative compared to those related to *energy efficiency* and *energy savings*, which have a more pronounced positive connotation. The statistical tests (S5 and S6 Figs) confirmed that positive and negative polarity are significantly higher in the concepts *energy efficiency* (p-value $<4.4\cdot 10^{-7}$) and *energy geopolitics* (p-value $<4.2\cdot 10^{-4}$), respectively.

We further assessed the semantic areas dominating each concept by calculating the average scores for the General Inquirer of the videos (the definitions of each semantic area are available in S1 File). Fig 5 presents the concepts with significantly higher scores compared to the others (detailed values are available in S7 Fig). The *energy efficiency* is significantly higher in the positive and virtue categories (p-value $<1.22\cdot 10^{-3}$), the latter defined by words indicating moral approval. The politics domain is dominated by *energy geopolitics*, *energy economy* and *energy model* (p-value $<3.66\cdot 10^{-3}$). In addition, the *energy geopolitics* is also significantly higher in the negative categories (p-value $<3.07\cdot 10^{-2}$) and most of the categories related to power, such as power control and total (p-value $<3.16\cdot 10^{-2}$). The concept of *energy storage* has high power loss scores, which could imply efficiency discussions. The *electricity market* displays significantly higher values in the economic (p-value $<2.06\cdot 10^{-9}$) and wealth (p-value $<8.45\cdot 10^{-3}$) domains and the *energy supply and demand* in the power ends (p-value $<6.80\cdot 10^{-10}$).

**Fig 6:**
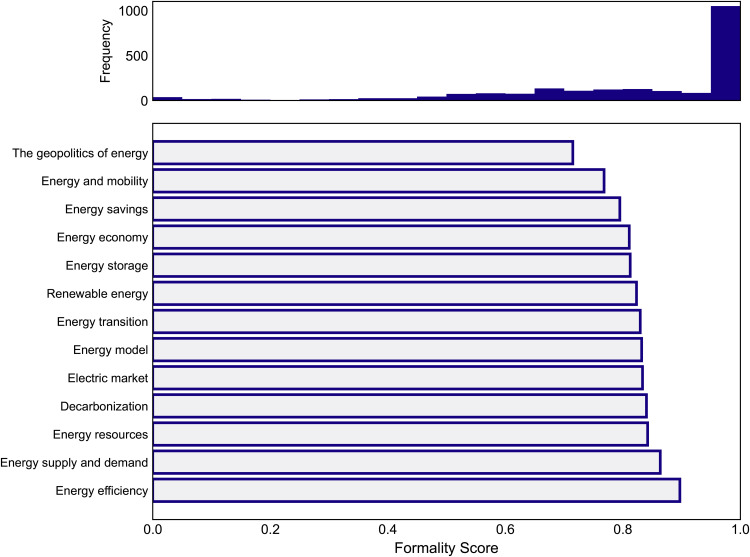
Language formality. (**a**) Distribution of formality scores in the analysed data set. (**b**) Average formality of the videos for each of the concepts.

An interesting feature to explore further in science communication, but not only, is the formality of language and its effect on engagement. We have computed the formality scores across our dataset, obtaining a distribution skewed to high values (Fig 6). The results suggest that the dissemination of content related to the energy transition field is overwhelmingly formal, which could indicate that it is often discussed with rigour. The average formality split by concept ranges from 0.7 to 1. While the *energy efficiency* and other related subjects have the highest formality scores, the most informal concept is the *energy geopolitics*.

**Fig 7:**
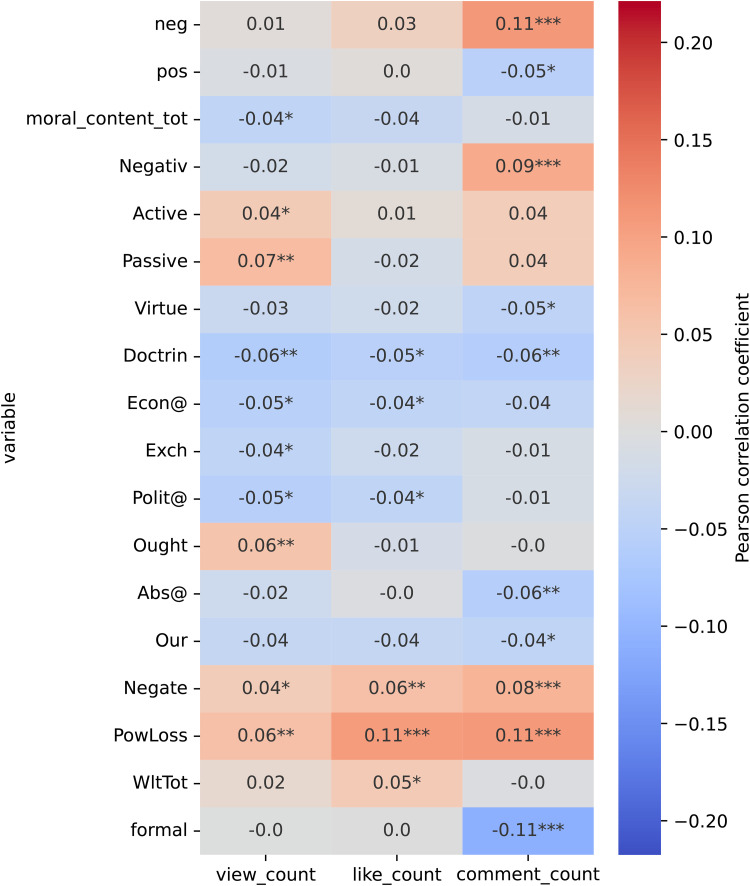
Correlation between the language features and the measures of content engagement. Correlation of the engagement metrics (number of views, likes, and comments) with the language features. We only show the combinations in which at least one of the engagement metrics has a significant correlation. Asterisks indicate the significance of the correlations (^*^p-value<0.05, ^**^p-value<0.01, ^***^p-value<0.001).

Finally, we analysed how the different language features identified in this study correlate with user engagement on YouTube, focusing on the number of views, likes, and comments (Fig 7). Overall, the correlations between engagement and content features are low, with only a few reaching statistical significance. The strongest correlations (in absolute value) are found for the negative tone (*r* = 0.11, $p=2.5\times 10^{-7}$), loss of power (*r* = 0.11, $p=3.9\times 10^{-7}$), and language formality (r=−0.11, $p=4.5\times 10^{-7}$). Higher scores in negative tone and loss of power are positively associated with more comments, whereas more formal language is associated with fewer comments.

### Original content creation and diffusion

#### Experimental design and content description.

We experimentally test the influence of language formality on content engagement by creating and publishing original content related to the energy transition on YouTube through two mirror channels that feature equal content but differentiated language use. The *Euro al Joule* [76] channel uses formal and scientific language grounded on data. It is narrated by academics, whereas the *eur2j* [77] channel uses a direct style mimicking natural conversation [78,79], with more casual and less technical terms, and is presented by a narrator with well-developed interpretative and communication abilities. Hereafter, we refer to *Euro al Joule* as the *academic channel*, and *eur2j* as the *conversational channel*. We provided consistency and coherence to the experiment by designing, recording, and publishing 20 videos on 20 challenges that represent more detailed versions of the concepts previously studied (See S1 File).

The first two videos were used to assess the diffusion of the content under neutral conditions, as they were published in the channels created in the context of this research. As of February 20, 2025, the videos published in August 2024 have 18 and 31 views in the academic channel, and 29 and 24 in the conversational channel, highlighting the difficulty of reaching a broad audience from scratch on the platform. The following three were used to conduct a series of tests with the Google Ads platform, which handles content promotion. Finally, the experiment focuses on the remaining 15 videos, whose broadcast was carried out identically on both channels. Four different content formats are published for each challenge: a promoted complete video, a non-promoted complete video for comparison, and two brief videos in standard and short versions [80]. The details of the Google Ads campaign parameters and the content format types are available in S1 File. The duration is the major factor that distinguishes complete and brief videos, along with the absence of supporting visual information (S8 Fig). While most brief videos are under 25 seconds, the complete videos are over a minute long, without distinction between promoted and non-promoted. The complete videos on the academic channel are longer, averaging around two minutes, compared to the conversational ones, which average around one minute. We applied the same methodology as in the previous section to measure the formality of original videos. The conversational channel content has lower formality scores and a more elongated distribution towards small values. The complete videos of both channels feature lower scores, as they might have a greater variety of vocabulary (S9 Fig).

#### Engagement analysis on YouTube.

We compared channels and formats by computing the mean and standard deviation of the three main engagement metrics: the views, likes, and comments (Table 1). The first general observation is that the number of likes and comments is very limited despite the promotion campaigns. There are almost no comments, and the average likes are only significant for brief videos in short format. Overall, the type of promotion has a higher effect on the content outreach than the channel and, therefore, language formality. The average number of views of the promoted complete videos is an order of magnitude higher than that of their non-promoted counterparts, going from around 10–100 views. Similarly, the average views of the brief content are one order of magnitude above the complete promoted one, going from around 100–1000 views.

**Table 1:** Summary of video engagement statistics by channel and format type. We show the mean (μ) and standard deviation (σ) of views, likes, and comments.

Channel	Promoted	N	Views (μ)	Views (σ)	Likes (μ)	Likes (σ)	Com. (μ)	Com. (σ)
Academic	Comp. not prom.	15	7.1	8.6	0.1	0.3	0	0
Conversational	Comp. not prom.	15	7	2.1	0.7	0.6	0.1	0.3
Academic	Comp. prom.	15	93.5	80.6	0.1	0.4	0	0
Conversational	Comp. prom.	15	100.2	47	0.2	0.4	0	0
Academic	Brief prom.	15	895.1	482.5	0.1	0.3	0	0
Conversational	Brief prom.	15	906.9	597.9	0	0	0.1	0.3
Academic	Brief (short) prom.	15	730.5	364.7	22.4	11	0	0
Conversational	Brief (short) prom.	15	936.5	529.5	21.5	10	0	0

**Fig 8:**
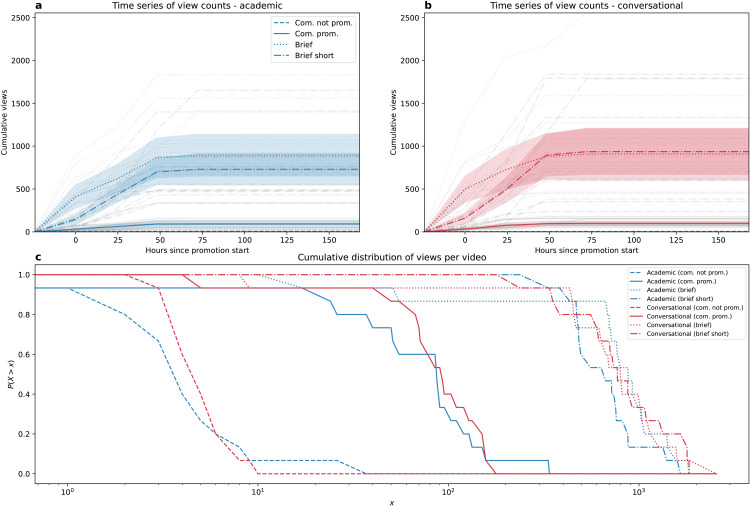
Evolution and cumulative distribution of views. Number of cumulative views depending on the hours since the promotion start date of each video by (**a**) the academic and (**b**) the conversational channel. We show in grey the values of each individual video, and in colour the average by type of content. (**c**) Inverse cumulative probability distributions of the views split by content type and channel.

We analyse the temporal evolution of views by calculating the daily cumulative views per channel and content format, aligning the content by the date of the promotion start (Fig 8a-8b). The daily values are higher for promoted content, especially brief videos. Although conversational videos have slightly higher average views, the differences are minor compared to the effect of promotion. The maximum increase in views is reached during the two days following the start of the promotion campaign, evidencing the promotion effect. The views flatten three days after the promotion, suggesting that the content does not draw further attention. The plateau reaches similar values for both channels, with the exception of the brief videos in short format, where the academic channel has an average of 750 views, and the conversational channel an average of 1000 views.

We assess the overall differences between channels and content format by calculating the inverse cumulative probability distribution of the total views per video (Fig 8c). The non-promoted complete videos have the fewest views, with none of them reaching over a hundred. Promoted complete videos have around a hundred views, while brief videos in either format have around one thousand views. We did not find noticeable differences between standard brief videos and brief videos in short format. According to the distributions, the influence of the channel and language use is limited compared to the promotion. Although the conversational channel has slightly higher views, the overall differences are minor, suggesting that promotion could mitigate the role of language in content engagement and its dissemination. We have conducted the Mann-Whitney U test [81] to identify significant differences between the distributions (S10 Fig). The results confirm that the differences in views per content format are statistically significant. The non-promoted content has significantly smaller views than the rest (p-value between $1.6\cdot 10^{-6}$ and $3.9\cdot 10^{-5}$). Similarly, the complete promoted one has significantly smaller views than the brief formats (p-value between $1.7\cdot 10^{-6}$ and $2.1\cdot 10^{-4}$). The comparison between the brief videos in standard and short formats did not yield significant differences in terms of views. When comparing the views between channels controlling for format, to evaluate if the language plays a role, we did not find statistically significant differences.

**Fig 9:**
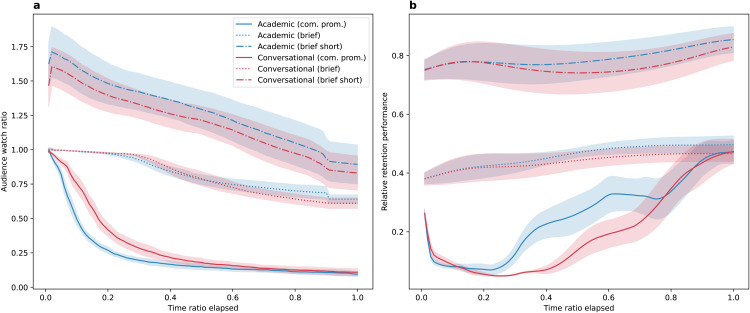
User retention by channel and content type. User retention metrics based on elapsed video ratio divided by content type. (**a**) *audience watch ratio* and (**b**) *relative retention performance*.

Beyond the engagement metrics, we have also analysed user retention. Specifically, we used the *audience watch ratio*, which is calculated as the number of times a part of the video was viewed divided by the total number of views, and the *relative retention performance*, which captures the retention performance of a fragment compared to other videos on the platform of similar length [68]. Thus, for a *relative retention performance* of 0.5, half of the videos of similar length have a better retention, and the other half have a worse retention. Similarly, a value greater than 0.5 implies that the fragment retention is better than most videos on the platform. The metrics are reported as a function of the *time ratio elapsed*, which stands for the fraction of content elapsed. In Fig 9, we show the evolution of the retention metrics split by channel and content type. The results for non-promoted complete videos are unavailable due to the limited visualisations.

According to the *audience watch ratio*, almost 75% of the audience watched the brief videos, while only 15% watched the complete videos in their entirety. The conversational channel (eur2j) has better retention. However, its shorter duration could bias the interpretation since the *time ratio elapsed* corresponds to a different temporal range in seconds. Instead, the *relative retention performance* offers a more balanced view of audience retention, as it is calculated relative to content of similar length, washing out the duration effect. There are no major differences in the brief content, but there are in the complete one. For the complete videos, the conversational channel has a better retention in the initial fragments, whereas the academic channel has a better retention in the central fragments. To assess whether these differences are statistically significant, we calculated the two-sided Mann-Whitney U test at each time ratio (S11 Fig), revealing that they are significant both at the beginning and in the central part. Interestingly, the channel with the highest value changes between the beginning and the middle sections. The conversational channel has a significantly higher retention between the time ratios 0.02 and 0.07 (p-value between 0.011 and 0.033), while the academic channel has a significantly higher retention between 0.23 and 0.7 (p-value between 0.045 and $3.9\cdot 10^{-5}$). These results suggest that conversational content is more efficient in retaining users at the beginning of the video, but academic content has better long-term retention.

#### Advertisement campaign analysis.

In this section, we analyse the data from the Google Ads platform [69] where the promotion was implemented, offering a complementary view to the YouTube data. The data is reported at the campaign level, aggregating the three promoted videos by challenge, and has additional engagement metrics and audience details. We have analysed the following three metrics:

- **Number of views.** Total number of views across all videos of the campaign. A view is counted when either a user interacts with the content or watches 30 seconds, or the entire video if it is shorter.
- **Percentage of views.** Percentage of users who watched a video when shown to them or when they saw the thumbnail. It is equivalent to the number of views divided by the number of impressions, which counts the advertisement views.
- **Percentage of interactions.** Percentage of users who interacted with the content when it was shown to them or when they saw the thumbnail. It is equivalent to the number of interactions divided by the number of impressions, which counts the advertisement views.

The Google Ads platform also reports details on audience characteristics. The YouTube metrics report views and retention, yet no sociodemographic information is provided. This disaggregation is essential for gaining a deeper understanding of engagement heterogeneities across the population, allowing for the interpretation of differences between channels and avoiding the overrepresentation of certain segments. In particular, we have focused on the age and gender of users, along with the device used to watch the videos. We have analysed the audience distribution by category, finding that it is composed of a majority of men (60%) in the older age groups (over 54) (S12 Fig).

**Fig 10:**
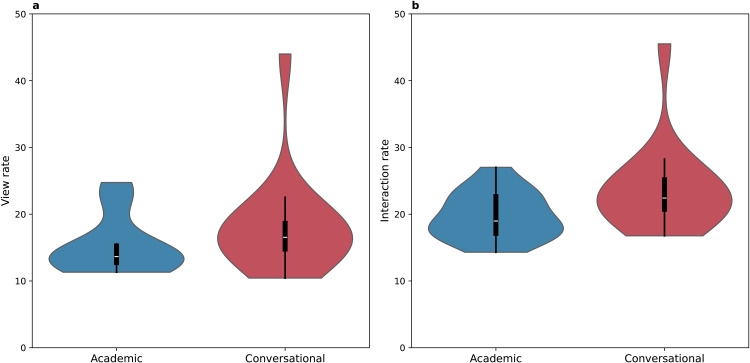
Distribution of the view and interaction rates. Distribution of (**a**) the viewing percentage and (**b**) the interaction rate of the promotion campaigns divided by conversational and academic content. In both cases, the distribution of conversational videos is significantly higher with p-values of 0.03 and 0.02, respectively.

Since the number of views was already analysed in the previous section, and does not account for additional factors such as advertisement impressions, we focus here on view rates and interaction rates as more informative engagement metrics. To assess differences on engagement between channels, we compared the distributions of view and interaction percentages (Fig 10). The results show that conversational videos exhibit significantly higher values for both metrics, indicating stronger audience engagement.

**Fig 11:**
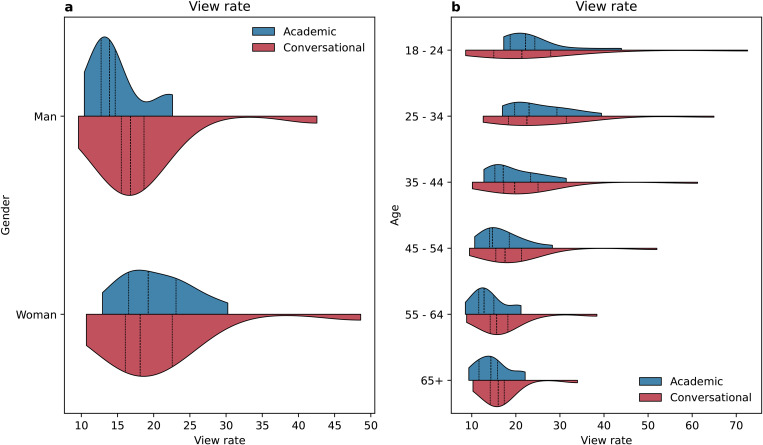
View rate by audience profile. Distribution of the view rate by (**a**) gender and (**b**) age.

The audience information allows us to compare the engagement across demographics by plotting the view percentage distribution for each characteristic (Fig 11). Women have higher view percentages for both channels, although the difference is more noticeable for the academic channel. Younger age groups have a higher percentage of views, especially individuals between 25 and 34. Conversational videos have a higher percentage of views across all age groups compared to the academic ones. We have conducted the Mann-Whitney U test between profiles to assess the significance of their differences (S13 Fig). The main statistically significant results by gender are that the percentage of views for the academic channel among women is higher than among men, regardless of the channel (p-value of $7\cdot 10^{-7}$ for the academic and 0.044 for the conversational), but the percentages for the conversational channel between genders are not significantly different. Similarly, the view percentage of men for the academic channel is significantly lower than any other combination of gender and channel (p-value between $7\cdot 10^{-7}$ and 0.018).

The tests by age reveal that the view percentage among individuals between 25 and 34 years for both channels is significantly higher than for those aged 35 or older. Conversely, the views of academic videos by people over 55 years old are significantly lower than those under 55 years old, regardless of the channel. In agreement with the previous results, the view percentage on computers is significantly higher than for other devices. Finally, we analysed the differences between the combinations of age and gender. Women aged between 25 and 34 have a significantly higher percentage of views compared to the vast majority of other age and gender profiles. In contrast, men over 45 have a statistically significantly lower percentage of views than most profiles. In summary, while men account for a higher number of total views, young women have a higher percentage of views when we consider the impressions. We conducted the same analysis for the percentage of interactions in S14 and S15 Figs, with similar findings. The audience retention by profile is consistent with our results on the view percentage, with young individuals and women watching a larger percentage of the content (S16 Fig).

## Discussion

Recent events, including the Iberian Peninsula blackout or the escalation of worldwide political tensions [13,82,83], have exposed the need for improved communication about the current and future challenges of energy management. This need is further amplified by the increasing risk of polarisation and political manipulation around the subject [84–86]. In the first part of this research, we analysed content from online social media platforms, motivated by the growing reliance on these channels for information consumption, particularly among younger generations [87]. We have characterised the YouTube content related to the energy transition, focusing on the topics discussed, the associated semantic areas, the emotions conveyed, and their language formality. The analysis of YouTube content revealed that there is a wide range of subtopics discussed around the energy transition, including practical subjects such as *energy efficiency*, focused on improving the efficiency of buildings and houses, and more political subjects such as *energy economy* or *energy geopolitics*, which explore market prices and examine how nations influence and shape global energy systems through policy decisions, resource control, and alliances. The polarity around each of the concepts is similar, with high values of neutrality, evidenced by the lack of terms with strong positive or negative connotations in the topic analysis. The results suggest that the energy transition is discussed in a factual manner without strong affective polarisation. Despite the emotional neutrality of the content, we identified certain outliers with higher emotional scores. Whereas the *energy efficiency* content is more positive, the *energy geopolitics* content is paired with negative emotions. The formality analysis aligns with the high neutrality scores as the content is shaped by formal language. Notably, the concept with higher negativity, *energy geopolitics*, also features lower formality scores. The results of the correlation analysis indicate that content characterised by negative sentiment and semantic themes related to power loss tends to generate a higher number of comments. In contrast, a higher level of formality is associated with reduced audience engagement, reflected by a lower comment count. Our results agree with previous research [57,88] and highlight the role of emotions in audience engagement. Although studies on the effect of language formality on engagement are limited, previous results from other platforms and contexts have found a positive relation between language informality and higher user engagement [89,90].

In the second part of our work, we empirically tested the relationship between language formality and engagement. Previous studies have already raised doubts about the role of content, showing how videos with high-quality content do not engage more than their low-quality counterparts [91]. Similarly, user-generated content receives higher engagement than professional content [59]. Our initial content publication in the newly created channels revealed difficulties in reaching a broad audience without prior visibility. To achieve a sufficient number of views for in-depth statistical analysis, we used the Google Ads platform to promote the content. The process of promotion had a significant effect compared to the base scenario, with a visualisation increase of three orders of magnitude. Although the conversational channel received slightly more views than the academic one, the difference was not statistically significant. The small sample size and high variance of engagement across videos might affect the results of the statistical tests. The detailed data reports from Google Ads enabled a fair comparison between channels and an assessment of the interest by demographic characteristics. The comparisons revealed that view and interaction rates are significantly higher in the conversational channel, suggesting that direct and casual language may improve user engagement and broaden the audience of energy transition content. The results on view and interaction rates complement previous studies that analysed a popular science YouTube channel and did not use promotion campaigns [60]. While they found that the majority of the audience was male, our results suggest that women are more engaged with the content when normalised by impressions. Our work also highlights that young people are more interested in the content related to the energy transition [87]. Notably, interest varies by age, gender, and other socioeconomic characteristics depending on the subject.

Our work summarises the discussions around the energy transition and assesses how language features can condition user engagement from an analytical and experimental perspective. Our work provides evidence on the need to reshape scientific dissemination to provide more trustful information on the energy transition and the challenges arising. Whether young individuals are inherently more interested in the content, or additional factors like platform typology or recommendation algorithms are involved, remains an open question. Our work emphasises the need for experimental studies to obtain insights into the role of content format in user engagement. The results might not be extrapolated to all social media but vary across platforms and audiences. Our static perspective opens the door to longitudinal studies that assess the temporal evolution of engagement in energy transition content based on internal platform dynamics and external events. Similarly, qualitative studies could provide more insights into the reasons behind the interest disparities in the content by sociodemographics.

### Limitations

This study presents several limitations that should be considered. First, the observational datasets were constructed using the YouTube API based on 13 predefined energy-related keywords. This approach defines the scope of data collection to mainstream energy transition topics, which may lead to an overrepresentation of such content and could partly explain the formality scores observed. At the same time, this strategy may underrepresent niche communities that discuss energy-related issues using different levels of formality. In addition, the video retrieval algorithm of the YouTube API is opaque and not publicly disclosed, which can generate uncertainty over the dataset composition, as we are unaware of hidden prioritisation or suppression mechanisms. Thus, we cannot ascertain how content attributes (e.g., video length, popularity, etc.) influence the type of content retrieved.

Regarding the experimental design, it is important to note that different narrators were used for each channel, which may introduce additional confounding factors. While the academic channel was narrated by academics, the conversational channel featured a narrator with interpretative and communication skills. Paralinguistic features may also influence engagement; therefore, the observed differences between channels may reflect not only variations in language but also differences in communicative style. These limitations highlight the need for future experimental designs in which a broader range of factors is analysed independently to gain deeper insights into the relationship between communication style and engagement.

## Conclusion

This study provides a combined observational and experimental assessment of how linguistic features relate to engagement with energy transition content on YouTube. By integrating large-scale content analysis with a controlled communication experiment, we examine the role of sentiment, thematic framing, and language formality in shaping audience interaction.

Our findings indicate that language style is a relevant dimension in the dissemination of energy-related content, with more conversational formats tending to be associated with higher levels of user engagement, particularly in terms of interaction rates. At the same time, engagement patterns vary across audience segments, suggesting that communication strategies may not have uniform effects across different demographic groups.

More broadly, these results highlight the importance of aligning scientific communication practices with the dynamics of digital platforms, where accessibility, tone, and framing can influence how information is received and interacted with. However, the relationship between communication style and engagement is mediated by platform-specific factors, including recommendation systems, visibility mechanisms, and audience composition, which should be considered when interpreting these findings.

Overall, this work contributes to a growing body of research on digital communication of complex societal challenges, offering empirical insights into how energy transition narratives circulate and engage audiences in online environments. Further research is needed to explore these dynamics across platforms, languages, and temporal scales, and to better understand how communication strategies can support informed and inclusive public engagement with energy transition processes.

## Supporting information

S1 File Supplementary Material for “An analytical and experimental approach to the discourse of the energy transition in YouTube”. (PDF)

S1 Fig Number of videos per year. (PDF)

S2 Fig Distribution of videos per concept. (PDF)

S3 Fig Distribution of engagement metrics. (PDF)

S4 Fig Statistics by concept for Spanish content. (PDF)

S5 Fig Statistical tests between positive polarity across concepts. (PDF)

S6 Fig Statistical tests between negative polarity across concepts. (PDF)

S7 Fig Polar plots by concept. (PDF)

S8 Fig Distribution of the duration of the videos by channel and video type. (PDF)

S9 Fig Distribution of formality scores. (PDF)

S10 Fig Mann-Whitney U test between views distributions. (PDF)

S11 Fig Significance of retention values for the full promoted content. (PDF)

S12 Fig Audience distribution. (PDF)

S13 Fig Statistical tests between the percentage of views by audience profile. (PDF)

S14 Fig Interaction rate by audience profile. (PDF)

S15 Fig Statistical tests between the interaction rate by audience profile. (PDF)

S16 Fig Video retention based on audience profile. (PDF)

## References

[1] Smil V. Energy and Civilization: A History. MIT Press; 2017.

[2] United Nations. Sustainable Development Goal 7: Affordable and Clean Energy. 2015. https://sdgs.un.org/goals/goal7

[3] van Ruijven BJ, De Cian E, Sue Wing I. Amplification of future energy demand growth due to climate change. Nat Commun. 2019;10(1):2762. doi: 10.1038/s41467-019-10399-3 31235700 PMC6591298

[4] Friedrich R, Voss A. External costs of electricity generation. Energy Policy. 1993;21(2):114–22. doi: 10.1016/0301-4215(93)90133-z

[5] Malla S. CO2 emissions from electricity generation in seven Asia-Pacific and North American countries: a decomposition analysis. Energy Policy. 2009;37(1):1–9.

[6] Sakulniyomporn S, Kubaha K, Chullabodhi C. External costs of fossil electricity generation: health-based assessment in Thailand. Renew Sustain Energy Rev. 2011;15(8):3470–9.

[7] Rodrigues JF, Wang J, Behrens P, de Boer P. Drivers of CO2 emissions from electricity generation in the European Union 2000–2015. Renew Sustain Energy Rev. 2020;133:110104.

[8] Solomon BD, Krishna K. The coming sustainable energy transition: history, strategies, and outlook. Energy Policy. 2011;39(11):7422–31.

[9] Østergaard PA, Duic N, Noorollahi Y, Kalogirou SA. Recent advances in renewable energy technology for the energy transition. 2021.

[10] Lee J, Yang JS. Global energy transitions and political systems. Renew Sustain Energy Rev. 2019;115:109370.

[11] Carley S, Evans TP, Konisky DM. Adaptation, culture, and the energy transition in American coal country. Energy Res Soc Sci. 2018;37:133–9.

[12] Markard J. The next phase of the energy transition and its implications for research and policy. Nature Energy. 2018;3(8):628–33.

[13] Kuzemko C, Blondeel M, Dupont C, Brisbois MC. Russia’s war on Ukraine, European energy policy responses & implications for sustainable transformations. Energy Res Soc Sci. 2022;93:102842. doi: 10.1016/j.erss.2022.102842

[14] Steg L, Perlaviciute G, van der Werff E. Understanding the human dimensions of a sustainable energy transition. Front Psychol. 2015;6:805. doi: 10.3389/fpsyg.2015.00805 26136705 PMC4469815

[15] Komendantova N, Neumueller S. Discourses about energy transition in Austrian climate and energy model regions: Turning awareness into action. Energy Environ. 2020;31(8):1473–97. doi: 10.1177/0958305x20907086

[16] Seran K, Rotimi J, Le A. Decision making support tool for renewable energy prioritization to achieve sustainable development goals (SDGs): Conceptual framework. Energy Environ Sustain. 2025;1(4):100044. doi: 10.1016/j.eesus.2025.100044

[17] Xia Y, Wang K, Huang Y, Lin T, Shi L, Wu F. Bounded rational decision-making modeling and analysis in local energy markets: A state-of-the-art review. Renew Sustain Energy Rev. 2026;226:116310. doi: 10.1016/j.rser.2025.116310

[18] Ji T, Wang S, Wang J, Zhang J. Beyond technology: Role of technology-organization-environment-human factors in autonomous driving adoption for new energy vehicles. J Retail Consum Serv. 2026;88:104552.

[19] Althaus SL, Tewksbury D. Patterns of internet and traditional news media use in a networked community. Polit Commun. 2000;17(1):21–45. doi: 10.1080/105846000198495

[20] Fletcher R, Park S. The impact of trust in the news media on online news consumption and participation. Dig Jour. 2017;5(10):1281–99.

[21] George AL. Propaganda analysis: A study of inferences made from Nazi propaganda in World War II. The University of Chicago; 1959.

[22] Krippendorff K. Content analysis: An introduction to its methodology. Sage Publications; 2018.

[23] Eichstaedt JC, Kern ML, Yaden DB, Schwartz HA, Giorgi S, Park G, et al. Closed- and open-vocabulary approaches to text analysis: A review, quantitative comparison, and recommendations. Psychol Methods. 2021;26(4):398–427. doi: 10.1037/met0000349 34726465

[24] Pennebaker JW, Francis ME, Booth RJ. Linguistic inquiry and word count: LIWC 2001. Mahway: Lawrence Erlbaum Associates; 2001.

[25] Hutto C, Gilbert E. Vader: A parsimonious rule-based model for sentiment analysis of social media text. In: Proceedings of the international AAAI conference on web and social media. Vol. 8. 2014. pp. 216–225.

[26] Stone PJ, Dunphy DC, Smith MS, Ogilvie DM. The general inquirer: A computer approach to content analysis. Cambridge, MA: MIT Press; 1966.

[27] Heylighen F, Dewaele JM. Formality of language: definition, measurement and behavioral determinants. Interner Bericht, Center “Leo Apostel”, Vrije Universiteit Brüssel. 1999.

[28] Griffiths TL, Steyvers M, Tenenbaum JB. Topics in semantic representation. Psychol Rev. 2007;114(2):211–44. doi: 10.1037/0033-295X.114.2.211 17500626

[29] Catal C, Nangir M. A sentiment classification model based on multiple classifiers. Appl Soft Comput. 2017;50:135–41.

[30] Foltz PW. Using latent semantic indexing for information filtering. SIGOIS Bull. 1990;11(2–3):40–7. doi: 10.1145/91478.91486

[31] Steyvers M, Griffiths T. Handbook of latent semantic analysis. Psychology Press; 2007. pp. 439–60.

[32] Blei DM, Ng AY, Jordan MI. Latent dirichlet allocation. J Mach Learn Res. 2003;3(Jan):993–1022.

[33] Reuter A, Thielmann A, Weisser C, Safken B, Kneib T. Probabilistic topic modeling with transformer representations. IEEE Trans Neural Netw Learn Syst. 2025;36(8):14551–65. doi: 10.1109/TNNLS.2025.3538262 40036454

[34] Singh M, Jakhar AK, Pandey S. Sentiment analysis on the impact of coronavirus in social life using the BERT model. Soc Netw Anal Min. 2021;11(1):33. doi: 10.1007/s13278-021-00737-z 33758630 PMC7976692

[35] Catelli R, Pelosi S, Esposito M. Lexicon-based vs. Bert-based sentiment analysis: a comparative study in Italian. Electronics. 2022;11(3):374. doi: 10.3390/electronics11030374

[36] Ilyas SHW, Soomro ZT, Anwar A, Shahzad H, Yaqub U. Analyzing Brexit’s impact using sentiment analysis and topic modeling on Twitter discussion. In: Proceedings of the 21st Annual International Conference on Digital Government Research. 2020. pp. 1–6.

[37] Zhang MM, Wang X, Hu Y. Strategic framing matters but varies: A structural topic modeling approach to analyzing China’s foreign propaganda about the 2019 Hong Kong protests on Twitter. Soc Sci Comput Rev. 2023;41(1):265–85.

[38] Xu S, Xiong Y. Setting socially mediated engagement parameters: a topic modeling and text analytic approach to examining polarized discourses on Gillette’s campaign. Public Relat Rev. 2020;46(5):101959. doi: 10.1016/j.pubrev.2020.101959

[39] Tapi Nzali MD, Bringay S, Lavergne C, Mollevi C, Opitz T. What patients can tell us: topic analysis for social media on breast cancer. JMIR Med Inform. 2017;5(3):e23. doi: 10.2196/medinform.7779 28760725 PMC5556259

[40] Xue J, Chen J, Chen C, Zheng C, Li S, Zhu T. Public discourse and sentiment during the COVID 19 pandemic: using Latent Dirichlet Allocation for topic modeling on Twitter. PLoS One. 2020;15(9):e0239441. doi: 10.1371/journal.pone.0239441 32976519 PMC7518625

[41] Bassolas A, Massachs J, Cozzo E, Vicens J. Multifaceted polarization and information reliability in climate change discussions on social media platforms. R Soc Open Sci. 2025;12(11):241974. doi: 10.1098/rsos.241974 41307068 PMC12646745

[42] Wang H, Can D, Kazemzadeh A, Bar F, Narayanan S. A system for real-time twitter sentiment analysis of 2012 us presidential election cycle. In: Proceedings of the ACL 2012 system demonstrations. 2012. pp. 115–120.

[43] Sandoval-Almazan R, Valle-Cruz D. Facebook impact and sentiment analysis on political campaigns. In: Proceedings of the 19th annual international conference on digital government research: governance in the data age; 2018. pp. 1–7.

[44] Bhat M, Qadri M, Kundroo M, Ahanger N, Agarwal B, et al. Sentiment analysis of social media response on the Covid19 outbreak. Brain Behav Immun. 2020;87:136. 32418721 10.1016/j.bbi.2020.05.006 PMC7207131

[45] Liu S, Liu J. Public attitudes toward COVID-19 vaccines on English-language Twitter: a sentiment analysis. Vaccine. 2021;39(39):5499–505. doi: 10.1016/j.vaccine.2021.08.058 34452774 PMC8439574

[46] Nagpal M, Jalali N, Sherifali D, Morita P, Cafazzo JA. Investigating Reddit data on type 2 diabetes management during the COVID-19 pandemic using latent Dirichlet allocation topic modeling and valence aware dictionary for sentiment reasoning analysis: content analysis. JMIR Form Res. 2025;9:e51154. doi: 10.2196/51154 39983050 PMC11870598

[47] Nguyen T, Venkatesh S, Phung D. Large-Scale Stylistic Analysis of Formality in Academia and Social Media. Lecture Notes in Computer Science. Springer International Publishing; 2016. pp. 137–45. doi: 10.1007/978-3-319-48743-4_11

[48] Nguyen T, Venkatesh S, Phung D. Academia versus social media: A psycho-linguistic analysis. J Comput Sci. 2018;25:228–37. doi: 10.1016/j.jocs.2017.08.011

[49] Jenders M, Kasneci G, Naumann F. Analyzing and predicting viral tweets. In: Proceedings of the 22nd international conference on world wide web; 2013. pp. 657–664.

[50] Nesi P, Pantaleo G, Paoli I, Zaza I. Assessing the reTweet proneness of tweets: predictive models for retweeting. Multimed Tools Appl. 2018;77(20):26371–96. doi: 10.1007/s11042-018-5865-0

[51] Hoiles W, Aprem A, Krishnamurthy V. Engagement and popularity dynamics of YouTube videos and sensitivity to meta-data. IEEE Trans Knowl Data Eng. 2017;29(7):1426–37. doi: 10.1109/tkde.2017.2682858

[52] Lagnier C, Denoyer L, Gaussier E, Gallinari P. Predicting information diffusion in social networks using content and user’s profiles. In: Advances in Information Retrieval: 35th European Conference on IR Research, ECIR 2013, Moscow, Russia, March 24-27, 2013. Proceedings 35. Springer; 2013. pp. 74–85.

[53] Guille A, Hacid H, Favre C, Zighed DA. Information diffusion in online social networks. SIGMOD Rec. 2013;42(2):17–28. doi: 10.1145/2503792.2503797

[54] Li M, Wang X, Gao K, Zhang S. A survey on information diffusion in online social networks: models and methods. Information. 2017;8(4):118. doi: 10.3390/info8040118

[55] Dubovi I, Tabak I. Interactions between emotional and cognitive engagement with science on YouTube. Public Underst Sci. 2021;30(6):759–76. doi: 10.1177/0963662521990848 33546572 PMC8314998

[56] Ferrara E, Yang Z. Quantifying the effect of sentiment on information diffusion in social media. PeerJ Computer Science. 2015;1:e26. doi: 10.7717/peerj-cs.26

[57] Munaro AC, Hübner Barcelos R, Francisco Maffezzolli EC, Santos Rodrigues JP, Cabrera Paraiso E. To engage or not engage? The features of video content on YouTube affecting digital consumer engagement. J Consum Behav. 2021;20(5):1336–52. doi: 10.1002/cb.1939

[58] Shoufan A, Mohamed F. YouTube and education: a scoping review. IEEE Access. 2022;10:125576–99. doi: 10.1109/access.2022.3225419

[59] Welbourne DJ, Grant WJ. Science communication on YouTube: Factors that affect channel and video popularity. Public Underst Sci. 2016;25(6):706–18. doi: 10.1177/0963662515572068 25698225

[60] Yang S, Brossard D, Scheufele DA, Xenos MA. The science of YouTube: What factors influence user engagement with online science videos? PLoS One. 2022;17(5):e0267697. doi: 10.1371/journal.pone.0267697 35613095 PMC9132274

[61] Gretry A, Horváth C, Belei N, van Riel AC. Don’t pretend to be my friend! When an informal brand communication style backfires on social media. J Bus Res. 2017;74:77–89.

[62] Munaro AC, Barcelos RH, Francisco Maffezzolli EC, Rodrigues JPS, Paraiso EC. Does your style engage? Linguistic styles of influencers and digital consumer engagement on YouTube. Comput Hum Behav. 2024;156:108217. doi: 10.1016/j.chb.2024.108217

[63] Biddinika MK, Syamsiro M, Novianti S, Nakhshiniev B, Aziz M, Takahashi F. Dissemination of technology information through YouTube: a case of renewable energy technology. TELKOMNIKA (Telecommun Comput Electron Control). 2019;17(3):1526–38.

[64] Li F, Ullah A. Social media discourse as a window into energy transition: analyzing public perception of electric vehicles on YouTube. Energy Res Soc Sci. 2025;129:104402. doi: 10.1016/j.erss.2025.104402

[65] Google. YouTube Data API — Google for Developers — developers.google.com. [cited 2025 Mar 17]. https://developers.google.com/youtube/v3

[66] Google. Search — YouTube Data API — Google for Developers — developers.google.com. [cited 2025 Feb 18]. https://developers.google.com/youtube/v3/docs/search

[67] YouTube. YouTube Analytics and Reporting APIs — Google for Developers — developers.google.com. [cited 2025 May 26]. https://developers.google.com/youtube/analytics

[68] Google. Metrics — YouTube Analytics and Reporting APIs — Google for Developers — developers.google.com. [cited 2025 Jan 20]. https://developers.google.com/youtube/analytics/metrics

[69] Google. Overview — Google Ads API — Google for Developers — developers.google.com. [cited 2025 Feb 18]. https://developers.google.com/google-ads/api/docs/start

[70] Vasiliev Y. Natural language processing with Python and spaCy: A practical introduction. No Starch Press; 2020.

[71] Latorre JP, Amores JJ. Topic modelling of racist and xenophobic YouTube comments. Analyzing hate speech against migrants and refugees spread through YouTube in Spanish. In: Ninth International Conference on Technological Ecosystems for Enhancing Multiculturality (TEEM’21). 2021. pp. 456–60.

[72] Jelodar H, Wang Y, Rabbani M, Ahmadi SBB, Boukela L, Zhao R. A NLP framework based on meaningful latent-topic detection and sentiment analysis via fuzzy lattice reasoning on youtube comments. Multimedia Tools Appl. 2021;80:4155–81.

[73] Stone PJ, Hunt EB. A computer approach to content analysis: studies using the general inquirer system. In: Proceedings of the May 21-23, 1963, spring joint computer conference. 1963. pp. 241–256.

[74] Conneau A, Lample G. Cross-lingual language model pretraining. Adv Neural Inform Process Syst. 2019;32.

[75] Li C, Wang W, Balducci B, Hu L, Gordon M, Marinova D, et al. Deep formality: sentence formality prediction with deep learning. In: 2022 IEEE 23rd International Conference on Information Reuse and Integration for Data Science (IRI). IEEE; 2022. pp. 1–5.

[76] YouTube. eur2j — youtube.com. [cited 2025 Feb 18]. https://www.youtube.com/channel/UC4THWv0lD6jUptqADUetQow

[77] YouTube. Euro al Joule — youtube.com. [cited 2025 Feb 18]. https://www.youtube.com/channel/UCVWCo-QPq9R-aKzDU5UeIZQ

[78] Biel JI, Gatica-Perez D. VlogSense: Conversational behavior and social attention in YouTube. ACM Transactions on Multimedia Computing, Communications, and Applications (TOMM). 2011;7(1):1–21.

[79] Burgess J, Green J. YouTube: Online video and participatory culture. John Wiley & Sons; 2018.

[80] Google. Advice & Support from YouTube Shorts Community - YouTube Creators — youtube.com. [cited 2025 Mar 17]. https://www.youtube.com/intl/en_us/creators/shorts/

[81] McKnight PE, Najab J. Mann-Whitney U Test. The Corsini encyclopedia of psychology. 2010. pp. 1–1.

[82] Arfaoui N, Roubaud D, Naeem MA. Energy transition metals, clean and dirty energy markets: A quantile-on-quantile risk transmission analysis of market dynamics. Energy Econ. 2025;143:108250. doi: 10.1016/j.eneco.2025.108250

[83] León JGS. Spain-Portugal blackouts: what actually happened, and what can Iberia and Europe learn from it? — theconversation.com. [cited 2025 May 22]. https://theconversation.com/spain-portugal-blackouts-what-actually-happened-and-what-can-iberia-and-europe-learn-from-it-255666

[84] Żuk P, Szulecki K. Unpacking the right-populist threat to climate action: Poland’s pro-governmental media on energy transition and climate change. Energy Res Soc Sci. 2020;66:101485.

[85] Valquaresma A, Batel S, Afonso AI, Guerra R, Silva L. The renewable energy transition and “the People”–exploring the intersections of right-wing populism and the renewable energy transition in Portuguese Media Discourses. Environ Commun. 2024;18(7):847–61.

[86] Burgess MG, Van Boven L, Wagner G, Wong-Parodi G, Baker K, Boykoff M. Supply, demand and polarization challenges facing US climate policies. Nat Clim Change. 2024;14(2):134–42.

[87] Dam R, Elving WJ, van Veen R. Engaging millennials in the energy transition. In: Big Ideas in Public Relations Research and Practice. Emerald Publishing Limited; 2019. pp. 57–68.

[88] Robertson CE, Pröllochs N, Schwarzenegger K, Pärnamets P, Van Bavel JJ, Feuerriegel S. Negativity drives online news consumption. Nat Hum Behav. 2023;7(5):812–22. doi: 10.1038/s41562-023-01538-4 36928780 PMC10202797

[89] Rennekamp KM, Witz PD. Linguistic formality and audience engagement: investors’ reactions to characteristics of social media disclosures*. Contemporary Accting Res. 2021;38(3):1748–81. doi: 10.1111/1911-3846.12661

[90] Wu L, Li J, Qi J, Shi N, Zhu H. How to Promote Public Engagement and Enhance Sentiment Through Government Social Media During the COVID-19 Crisis. J Organ End User Comput. 2022;34(6):1–24. doi: 10.4018/joeuc.308819

[91] Desai T, Shariff A, Dhingra V, Minhas D, Eure M, Kats M. Is content really king? An objective analysis of the public’s response to medical videos on YouTube. PLoS One. 2013;8(12):e82469. doi: 10.1371/journal.pone.0082469 24367517 PMC3867348

