# Supplementary material for: An analytical and experimental study of the energy transition discourse on YouTube
Source: PLoS One. 2026 Jul 15;21(7):e0352691. doi: 10.1371/journal.pone.0352691 (PMC13372142; doi:10.1371/journal.pone.0352691)
Supplement: S1 File — (PDF) [file pone.0352691.s001.pdf]

Supplementary Material
An analytical and experimental approach to the discourse of the energy transition in YouTube

Aleix Bassolas, Piero Birello, and Julian Vicens

S1 Analysis of concepts related to energy

General statistical analysis

We analyse the number of videos per year in Fig. S1, where most of them are recent, with a steeper increase after 2020. Fig. S2 has the number of videos per concept after filtering the videos in Spanish and those including terms related to energy or electricity. The concepts with more videos are the *energy transition* and the *electric market*. The overall distribution of the engagement metrics (views, likes, and comments) reveals that views are higher, followed by likes and comments (Fig. S3). Despite the distributions having a clear peak, all of them are heavy-tailed, with a few videos featuring large values. In Fig. S4, we show the number of views, likes, comments, and the comment-to-view ratio. We also report two general characteristics of the content: the duration and the characters per second. There are disparities in concept engagement, with the *renewable energy* having more views, likes, and comments. Other concepts that gather more attention are the *energy supply and demand* and the *energy economy*. We also observe that the content has a large duration variability, ranging between 500 and 2000 seconds. Instead, the content speed, measured as characters per second, is very stable. The content interaction, measured as the ratio of comments and views, provides a complementary perspective to the results. In this case, concepts related to the economy, such as *electric market*, *energy supply and demand*, and the *energy economy*, are more salient.

Language analysis of energy content in YouTube

In Fig. S5, we provide the statistical tests between concepts for positive sentiments. The concept of *energy efficiency* has significantly higher values than the rest. Instead, the *electric market* has significantly lower positive scores. The other concept with significant differences in positive scores is *energy resources*. Regarding the negative sentiments (Fig. S6), multiple concepts have significantly higher values. The most notable ones are the *geopolitics of energy*, the *energy transition*, and the *energy supply and demand*.

The General Inquirer

We report here the chosen General Inquirer categories separated into four larger classes, with their corresponding definition:

- Broad or moral categories:
  - *Positiv*: 1,915 words of positive outlook (It does not contain words for yes, which has been made a separate category of 20 entries.)
  - *Negativ*: 2,291 words of negative outlook (not including the separate category no in the sense of refusal).
  - *Active*: 2045 words implying an active orientation.
  - *Passive*: 911 words indicating a passive orientation.
  - *Virtue*: 719 words indicating an assessment of moral approval or good fortune, especially from the perspective of middle-class society.

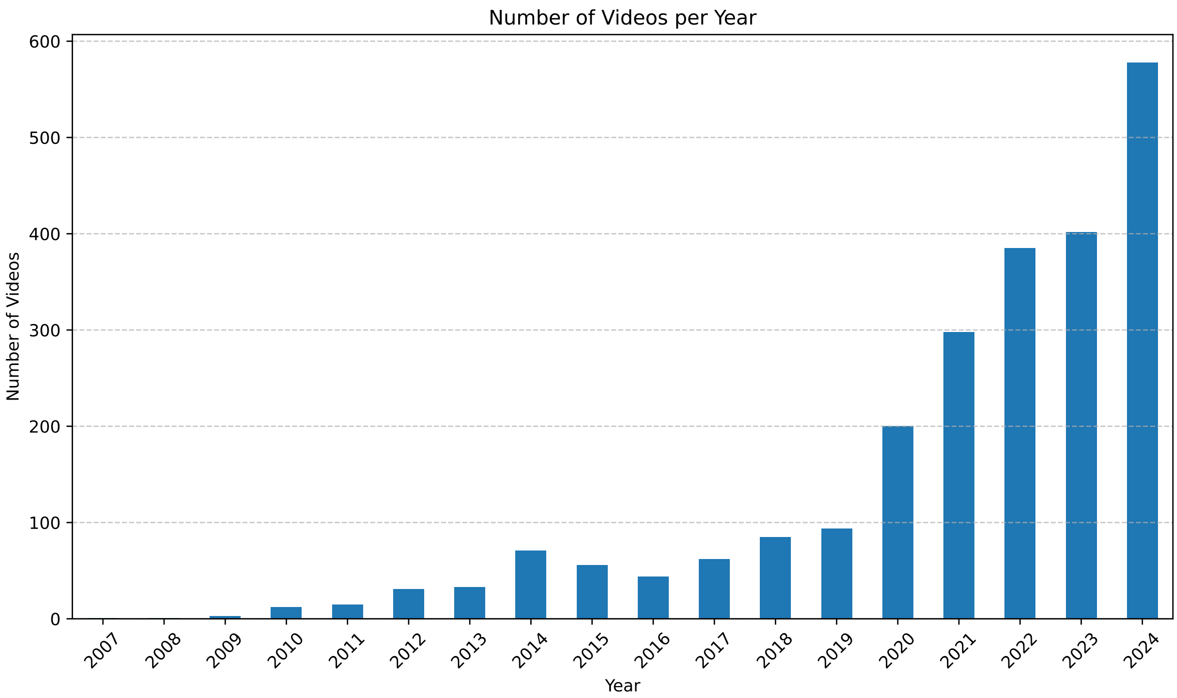

Figure S1: **Number of videos per year.** Number of videos per year in the dataset extracted from YouTube of videos related to energy.

- *Vice*: 685 words indicating an assessment of moral disapproval or misfortune.
- *Quan*: 314 words indicating the assessment of quantity, including the use of numbers.
- *PowTot*: = 1,266 words for the whole domain of power.
- *WltTot*: = 378 words in the wealth domain, where wealth is the valuing of having it.

- Knowledge, rigor or social domains:
  - *Academ* 153 words relating to academic, intellectual, or educational matters, including the names of major fields of study.
  - *Doctrin*: 217 words referring to organized systems of belief or knowledge, including those of applied knowledge, mystical beliefs, and arts that academics study.
  - *Econ@*: 510 words of an economic, commercial, industrial, or business orientation, including roles, collectivities, acts, abstract ideas, and symbols, including references to money. Includes names of common commodities in business.
  - *Exch*: 60 words concerned with buying, selling, and trading.
  - *Legal*: 192 words relating to legal, judicial, or police matters.
  - *Polit@*: 263 words having a clear political character, including political roles, collectivities, acts, ideas, ideologies, and symbols.
  - *Causal*: 112 words denoting presumption that the occurrence of one phenomenon is necessarily preceded, accompanied, or followed by the occurrence of another.
  - *Ought*: 26 words indicating moral imperative.
  - *Abs@*: 185 words reflecting tendency to use abstract vocabulary. There is also an ABS category (276 words) used as a marker.
  - *Negate*: has 217 words that refer to reversal or negation, including about 20 "dis" words, 40 "in" words, and 100 "un" words, as well as several senses of the word "no" itself; generally signals a downside view.

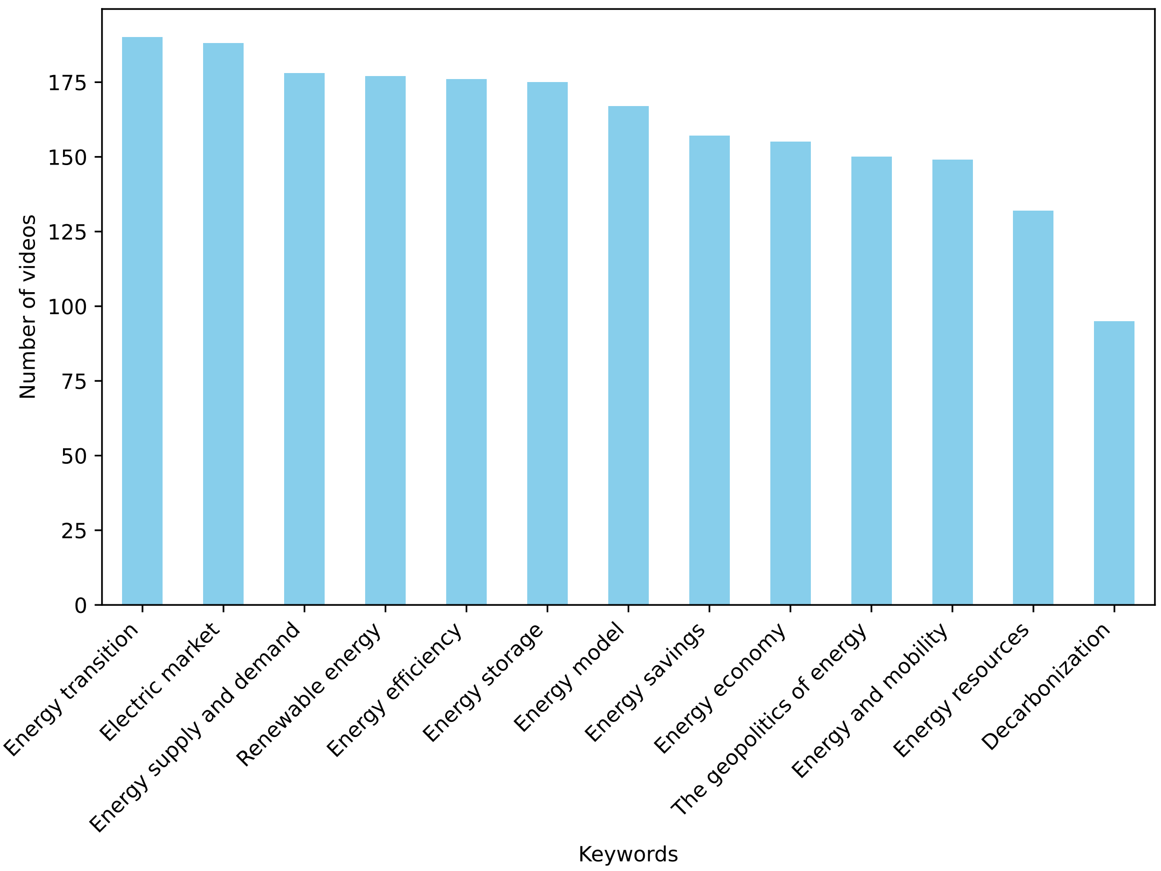

Figure S2: **Distribution of videos per concept.** Number of videos in the dataset assigned to each concept.

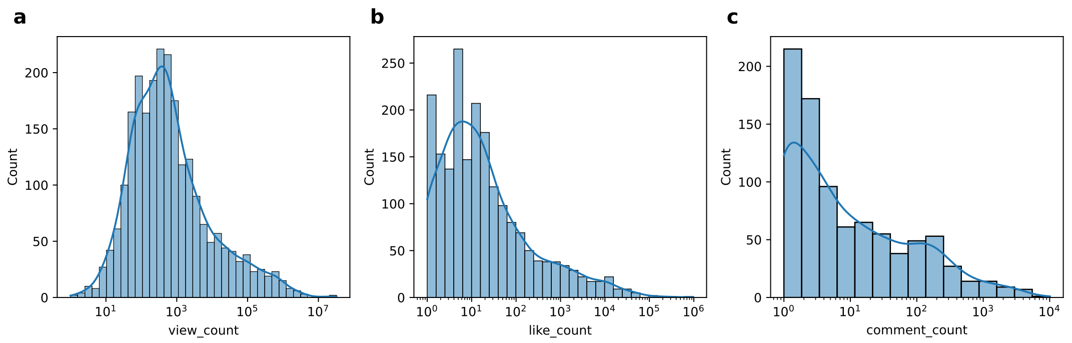

Figure S3: **Distribution of engagement metrics.** Distribution of **a** views, **b** likes and **c** comments.

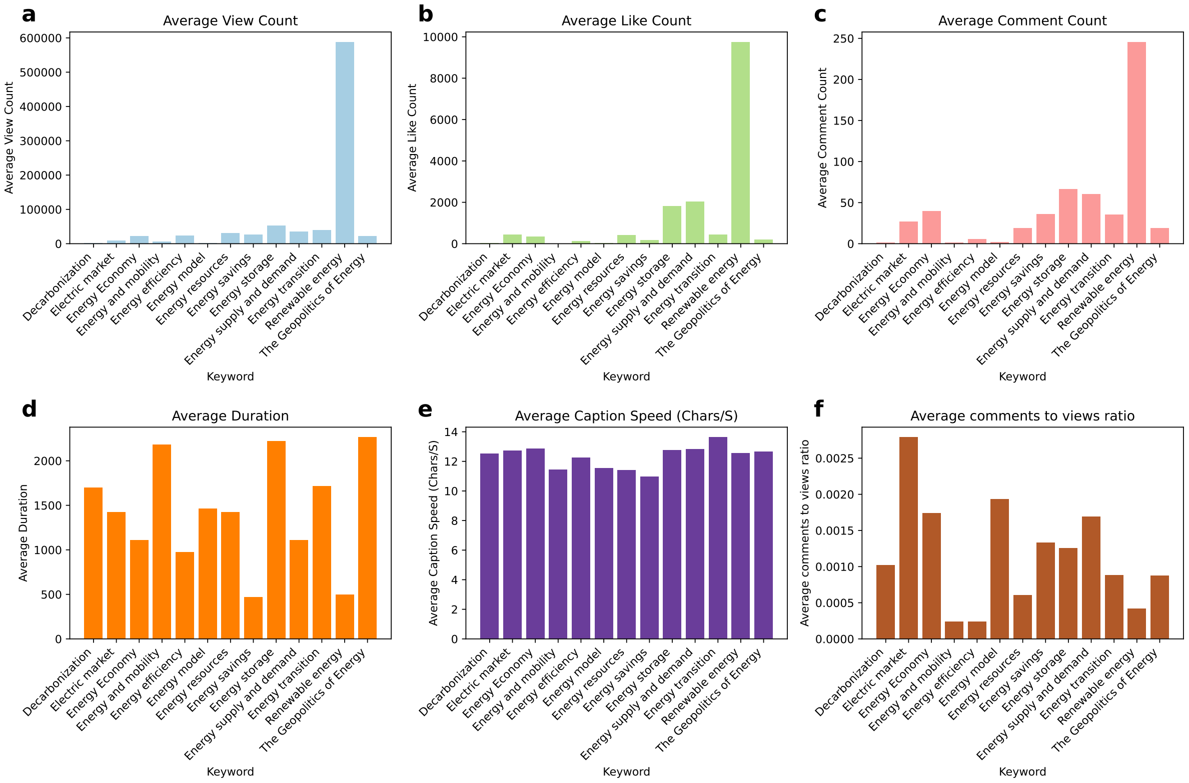

Figure S4: **Statistics by concept for Spanish content.** Average separated by concept of the main statistics: (**a**) views, (**b**) likes, (**c**) comments, (**d**) duration, (**e**) speed of the text, and (**f**) ratio between comments and views.

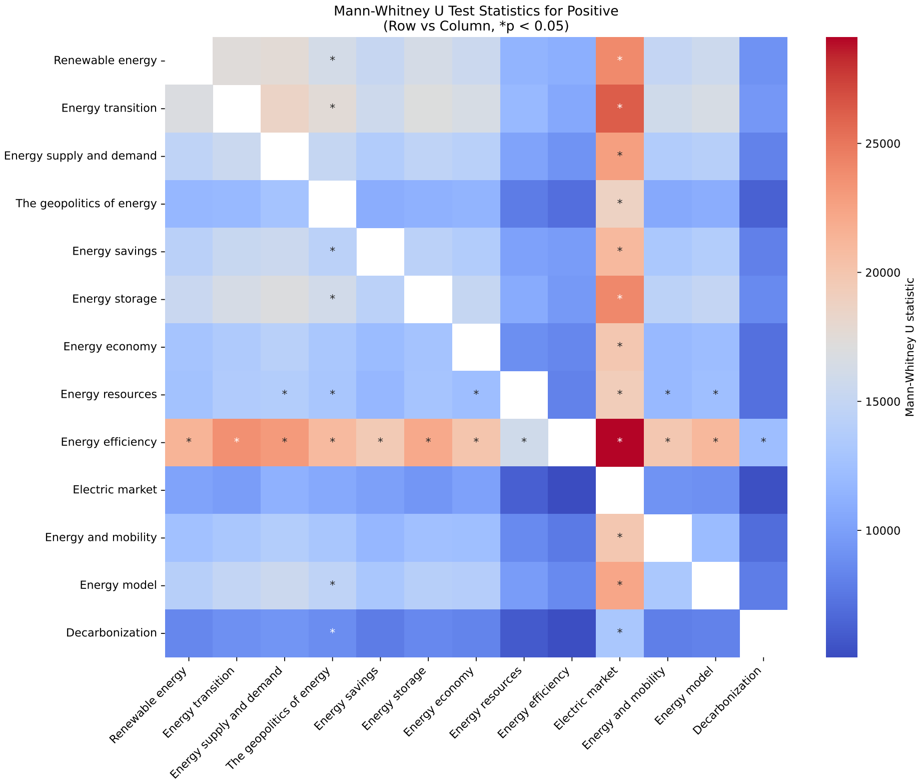

Figure S5: **Statistical tests between positive polarity across concepts.** Mann-Whitney U test between the positive polarity of concepts. The comparison is performed to assess whether concepts on the vertical axis are greater than those on the horizontal axis. Asterisks indicate that differences are significant.

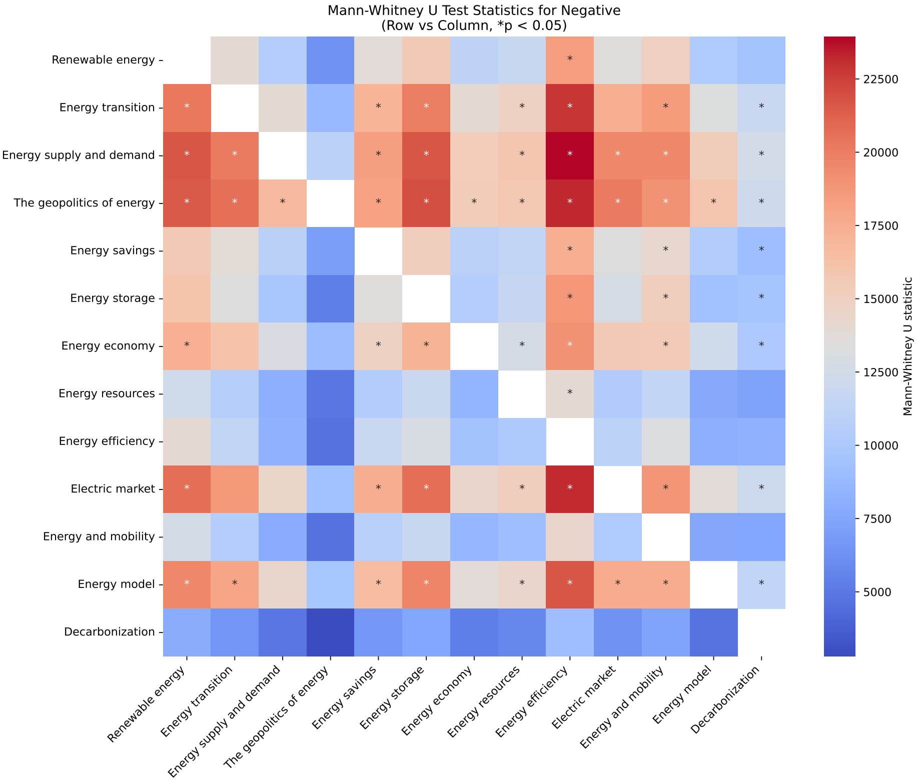

Figure S6: **Statistical tests between negative polarity across concepts.** Mann-Whitney U test between the negative polarity of concepts. The comparison is performed to assess whether concepts on the vertical axis are greater than those on the horizontal axis. Asterisks indicate that differences are significant.

- *Intrj*: has 42 words and includes exclamations as well as casual and slang references, words categorized "yes" and "no" such as "amen" or "nope", as well as other words like "damn" and "farewell".
  - *NUM*: 51 words indicating numbers.
- The self and the others:
  - *Self*: 7 pronouns referring to the singular self
  - *Our*: 6 pronouns referring to the inclusive self ("we", etc.)
  - *You*: 9 pronouns indicating another person is being addressed directly.
- Specific power categories:
  - *PowPt*: Power ordinary participants, 81 words for non-authoritative actors (such as followers) in the power process.
  - *PowCon*: Power conflict, 228 words for ways of conflicting.
  - *PowCoop*: Power cooperation, 118 words for ways of cooperating.
  - *PowAuPt*: Power authoritative participants, 134 words for individual and collective actors in the power process.
  - *PowGain*: Power Gain, 65 words about power increasing.
  - *PowLoss*: Power Loss, 109 words of power decreasing.
  - *PowEnds*: Power Ends, 30 words about the goals of the power process.
  - *PowAren*: Power Arenas, 53 words referring to political places and environments except nation-states.

In Fig. S7, we provide the detailed scores in each of the semantic areas analysed by concept. The *energy efficiency* stands out in the values of virtue and positive domains. The *electric market* has higher values in the quantitative domain and the *geopolitics of energy* in the power domain. In relation to the self categories, the *energy transition* has higher values in the self and the *energy savings* in the you. The power domain shows a wide heterogeneity across concepts. For example, the *decarbonisation* has high values in the power gain and power cooperation, the *energy supply and demand* in the power ends, and the *geopolitics of energy* in the power control.

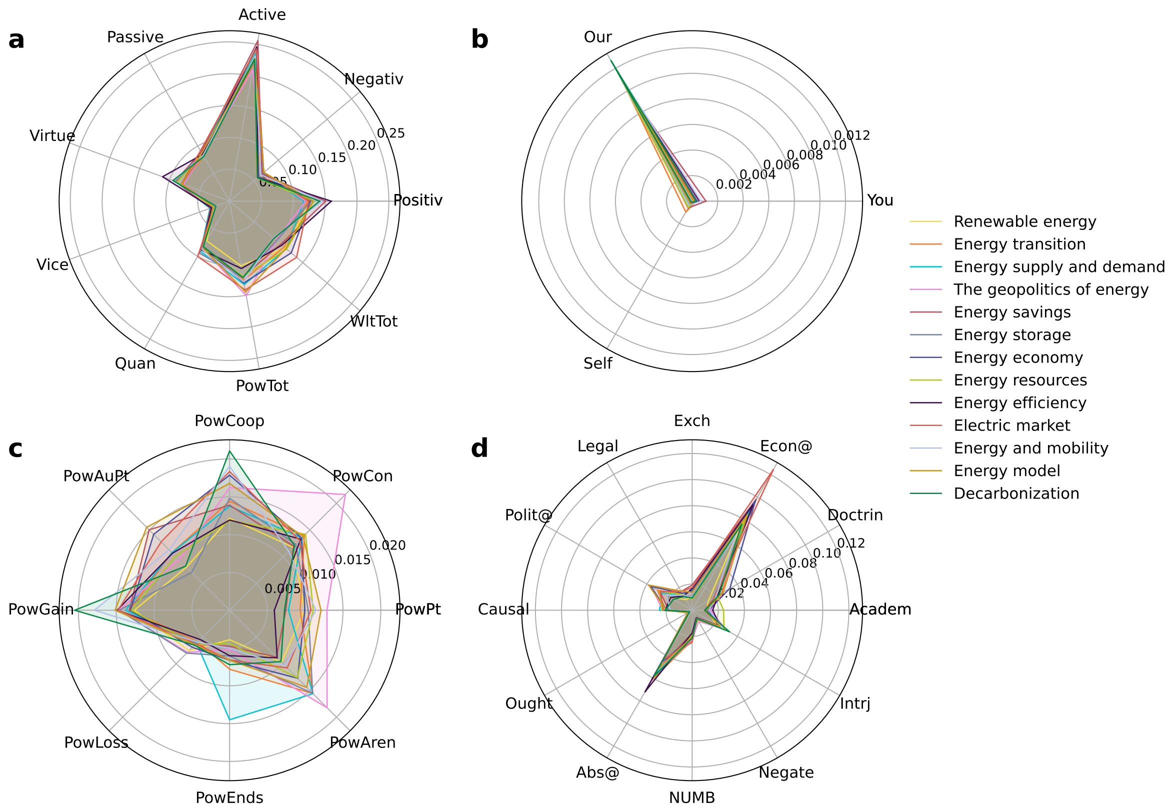

Figure S7: **Polar plots by concept.** Scoring of the concepts by category, depending on the concept to which the content belongs. (**a**) Moral, (**b**) self and others, (**c**) power, and (**d**) knowledge categories.

S2 Diffusion of original content on YouTube

Experimental design

Description of the challenges addressed by each video

The 20 challenges we selected to create the YouTube videos are the following:

- **Challenge 1.** Determine the level of rigour of information sources in the energy field.
- **Challenge 2.** Assess energy efficiency associated with technological improvements.
- **Challenge 3.** Assess energy efficiency associated with behavioural changes.
- **Challenge 4.** Identify the energy resources and technologies that govern decarbonisation strategies on a global scale.
- **Challenge 5.** Differentiate between energy and power requirements.
- **Challenge 6.** Distinguish the nature of the limitations associated with the use of different energy resources.
- **Challenge 7.** Understand the statistical nature of the different future energy scenarios.
- **Challenge 8.** Understand the implications of adopting a distributed energy model.
- **Challenge 9.** Identify the energy needs of the different mobility models.
- **Challenge 10.** Understand the need for the electrification of the current energy model.
- **Challenge 11.** Understand the need for control and regulation of supply and demand in distributed energy models.
- **Challenge 12.** Characterise the main energy storage strategies and technologies.
- **Challenge 13.** Identify the main materials and critical flows associated with the implementation of new energy models.
- **Challenge 14.** Critically evaluate the time scales (natural and anthropic) associated with the use of resources.
- **Challenge 15.** Identify the implications and conflicts at the territorial management level associated with each energy model.
- **Challenge 16.** Identify the conflicts at the level of demographic and economic management associated with each energy model.
- **Challenge 17.** Identify the geopolitical implications of the adoption of certain energy utilisation technologies.
- **Challenge 18.** Identify the ethical and social implications of each energy model.
- **Challenge 19.** Assess the role of research and technological innovation in the transformation of the energy model.
- **Challenge 20.** Understand the concepts of balance and complexity applied to the context of energy management.

In [1] and [2] the academic and conversational channels are available online respectively. Their aesthetics and design are very aligned, as well as the video thumbnails differing mainly in the speaker.

Promotion campaign

The parameters used in the promotion campaign through Google Ads are the following:

- **Campaign objective**. Video campaign with the view objective to achieve the maximum number of views during the promotion.
- **Multi-format ad.** 3 versions of each video are used: long, brief, and brief in short format.
- **Diffusion platform.** YouTube and the linked channels of the Display network. The Display Network consists of Google-owned websites such as YouTube, Google Finance, Gmail, and other addresses that serve Display advertising. It also includes a network of millions of websites and mobile apps from Google partners.
- **User device languages.** The content is displayed for those users whose device language is set to Catalan or Spanish.
- **Locations.** The geographical locations where the campaign has been shown are: Barcelona, Girona, Lleida, Tarragona, the Balearic Islands, and the Valencian Community.
- **Broadcast times.** Two different times have been used during which the content is shown.
  From Monday to Friday: from 8:30 am to 11:45 pm.
  Saturday and Sunday: from 12:30 pm to 11:45 pm.
- **Device-Specific Targeting.** The campaign is displayed on desktop computers, tablets and mobile phones. Campaign not showing on Smart TV devices.

For each channel and challenge, 4 types of video have been created that vary in length, format, and promotion. These typologies are the following:

- **Complete not promoted video (Complete not promoted).** This content includes the full video explaining each of the energy challenges.
- **Complete promoted video (Complete promoted).** The full promoted video is identical to the non-promoted one except for the opening static image.
- **Brief promoted.** This video summarises the full video, where no supporting images are shown but only the narrator.
- **Short video in short promoted format (Brief - short promoted).** This video has similar content to the short video, but in a short format where the image is framed vertically.

Brief videos, either in short format or not, have a very similar duration across channels at around 20 seconds (Fig. S8). However, the length of complete videos vary on length depending on the channel. Whereas the video duration in the conversational channel is around one minute, the video duration in the academic channel is around two minutes.

We have analysed the video formality scores by channel and content format using the same methodology as in the YouTube content analysis (Fig- S9). For all content formats, the conversational channel has a lower formality average and a distribution more elongated towards lower values.

Analysis of YouTube data

In Fig. S10, we provide the Mann-Whitney U statistical tests between content formats divided by channel, comparing whether the entries on the vertical axes are larger than those in the horizontal axes. Entries marked in black correspond to statistically significant differences (p-value<0.05). There are no significant differences between channels, but there are between content format and type of promotion. Non-promoted content has significantly lower views than promoted content. Similarly, the complete promoted content has significantly lower views than brief videos. We did not find particular differences between short and standard brief videos.

In Fig. S10, we provide the two-way Mann-Whitney U test of the relative retention performance between channels. The horizontal black indicates a p-value of 0.05. The difference between the channels is significant during the first portion of the videos and in the central part.

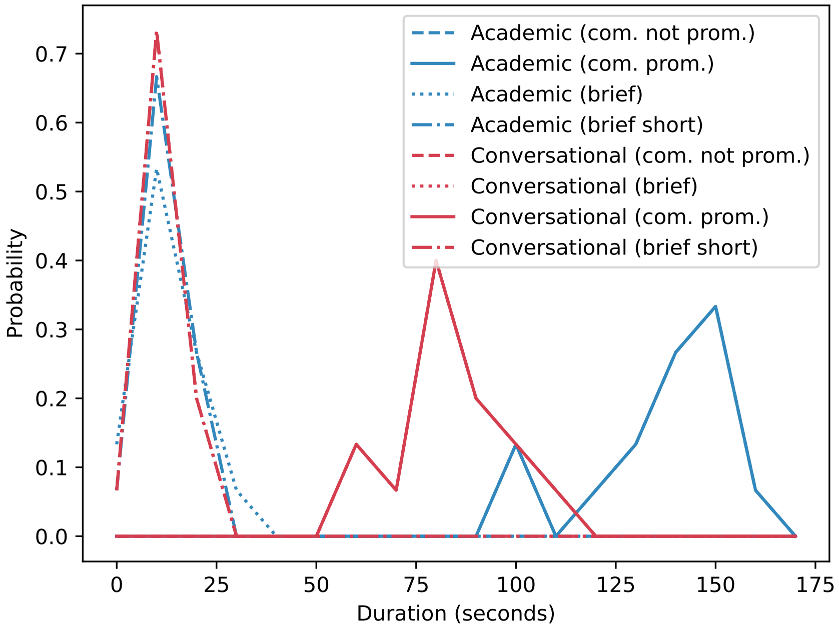

Figure S8: **Distribution of the duration of the videos by channel and video type.** Distribution of the duration in seconds (duration in seconds) of the videos for the Euro al Joule (blue) and eur2j (red) channels. Content has been separated by type: full videos (dashed line), full promoted videos (solid line), short videos (dotted line), and short format short videos (dashed and dotted line).

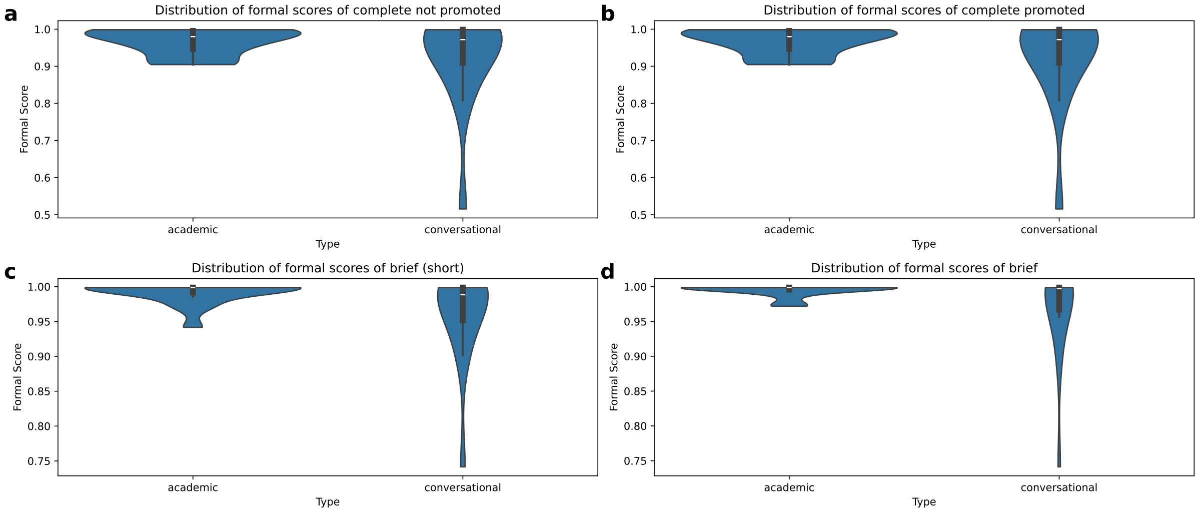

Figure S9: **Distribution of formality scores.** Distribution of formality scores for original videos separated by content (**a**) full non-promoted, (**b**) full promoted, (**c**) short in short format, and (**d**) standard short.

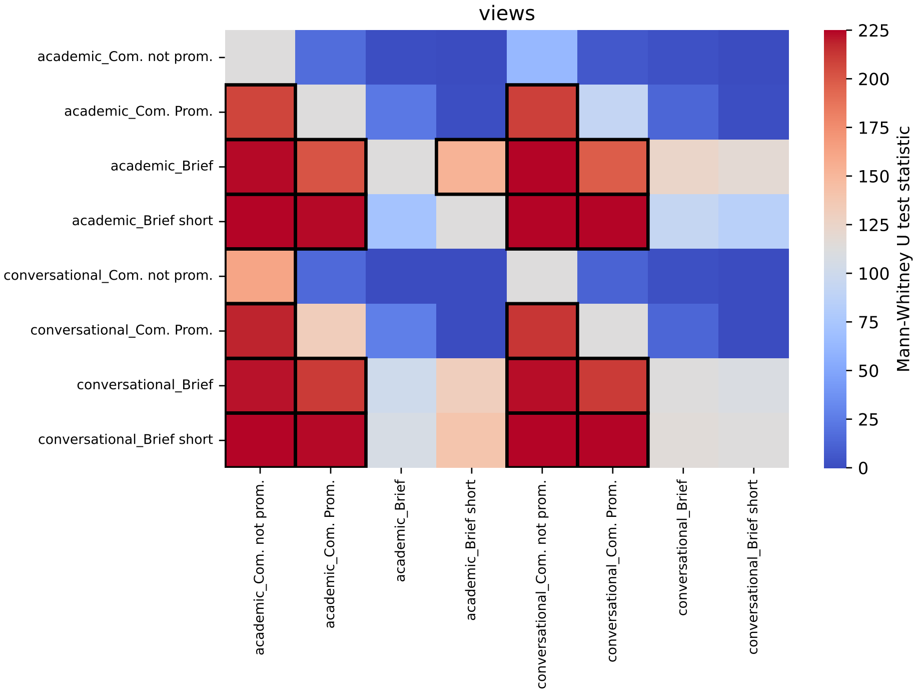

Figure S10: **Mann-Whitney U test between views distributions.** Results of the Mann-Whitney U test of the distributions of views per video by channel and content type. Each box includes the test value. In each statistical test, we evaluated whether the distribution of the content on the vertical axis is greater than that of the content on the horizontal axis. Entries marked in black correspond to statistically significant tests (p-value<0.05).

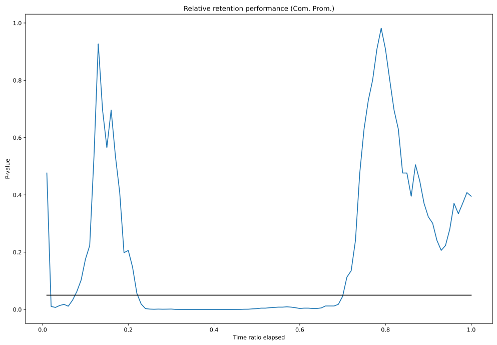

Figure S11: **Significance of retention values for the full promoted content.** Significance (p-value) between the relative retention performance values of each channel in the promoted long videos by elapsed video time. The black line stands for the significance value (p-value=0.05).

Analysis of Google Ads data

We have analysed the audience profile, revealing a slight over-representation of men and users older than 54 years (Fig. S12). Phones are the most common devices, although the visualisations from personal computers are not negligible.

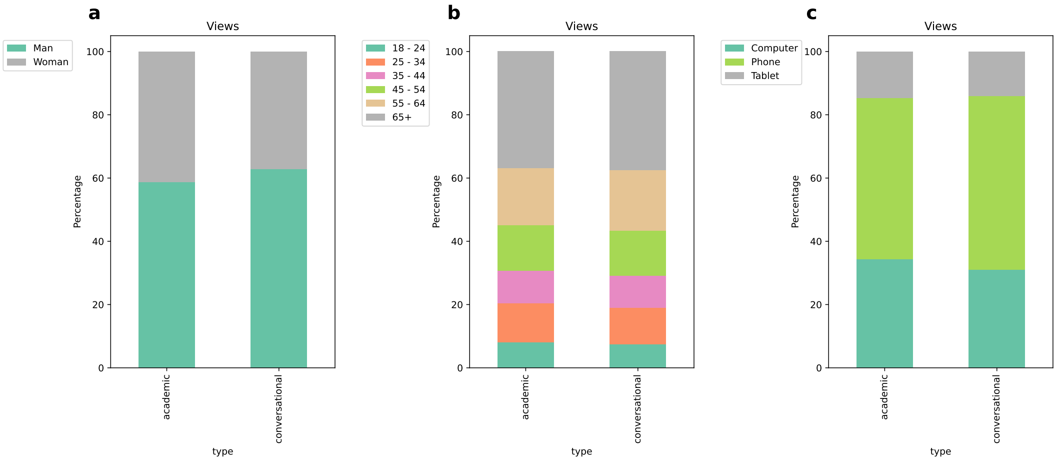

Figure S12: **Audience distribution** Distribution of the audience by (**a**) gender , (**b**) age (**c**) device.

We have performed Mann-Whitney U statistical tests of view rates by typology of audience (Fig. S13). The high view rates from women, young users, and computers are significant in most cases.

In Fig. S14, we show the distribution of the rate of interactions by gender profile, age, device, and combinations of age and gender. The results are similar to the view rates, with the views, women, and young individuals having higher interaction rates compared to men and older persons. The statistical tests reinforce the results (Fig. S15), with the interaction rate of men for the academic channel being significantly smaller than the rest.

In Fig. S16, we can see what percentage of users have viewed 25%, 50%, 75%, and 100% of the content, divided by profile and channel. In line with previous results, women show greater retention. For example, only 20% of men watch the academic videos in their entirety while 30% of women do. There are also notable differences in age, as 40% of users in the 25-34 age range view the content entirely, while less than 20% do so in the age group over 54. Among young individuals, we also see the smallest differences in retention between informal and academic videos. The retention analysis by device reveals that views on computers tend to last longer than on other devices.

References

[1] YouTube. Euro al Joule — youtube.com. `https://www.youtube.com/channel/UCVWCo-QPq9R-aKzDU5UeIZQ`, [Accessed 18-02-2025].

[2] YouTube. eur2j — youtube.com. `https://www.youtube.com/channel/UC4THWv0lD6jUptqADUetQow`, [Accessed 18-02-2025].

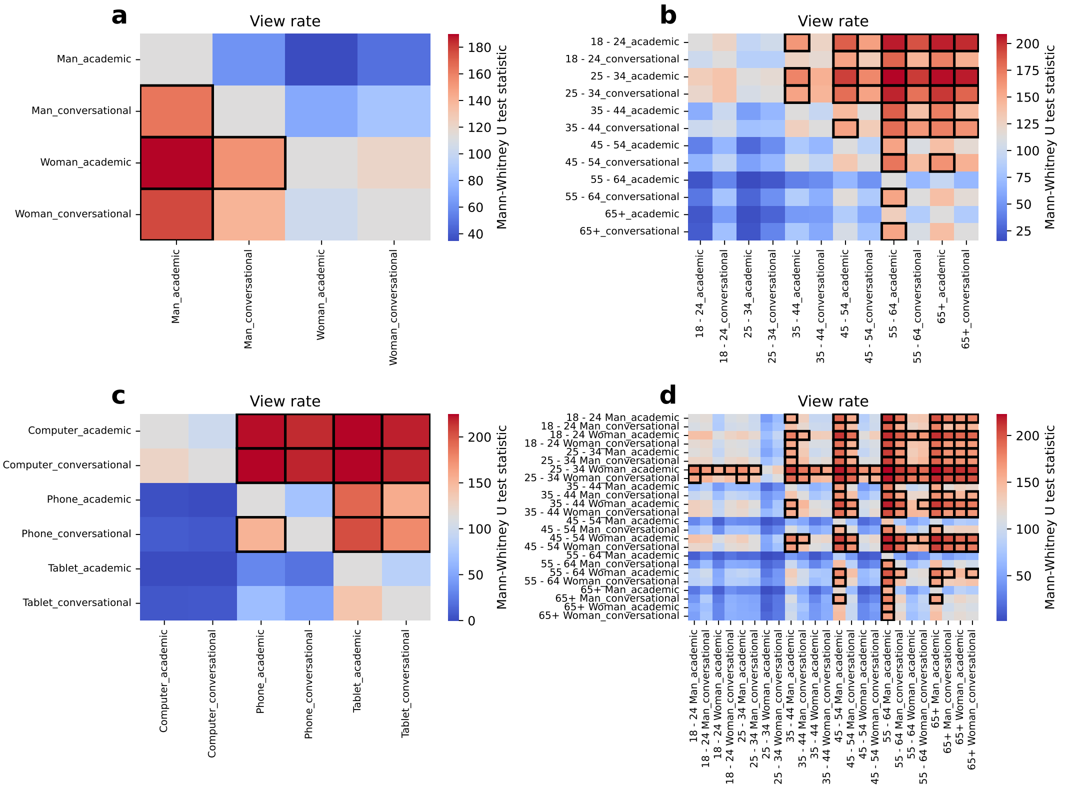

Figure S13: **Statistical tests between the percentage of views by audience profile.** Results of the statistical tests between the distributions of the view rate by (**a**) gender , (**b**) age (**c**) device, and (**d**) the combination of age and gender. In each statistical test, we evaluated whether the distribution of the group on the vertical axis is greater than that of the group on the horizontal axis. Entries marked in black show statistically significant tests (p-value<0.05).

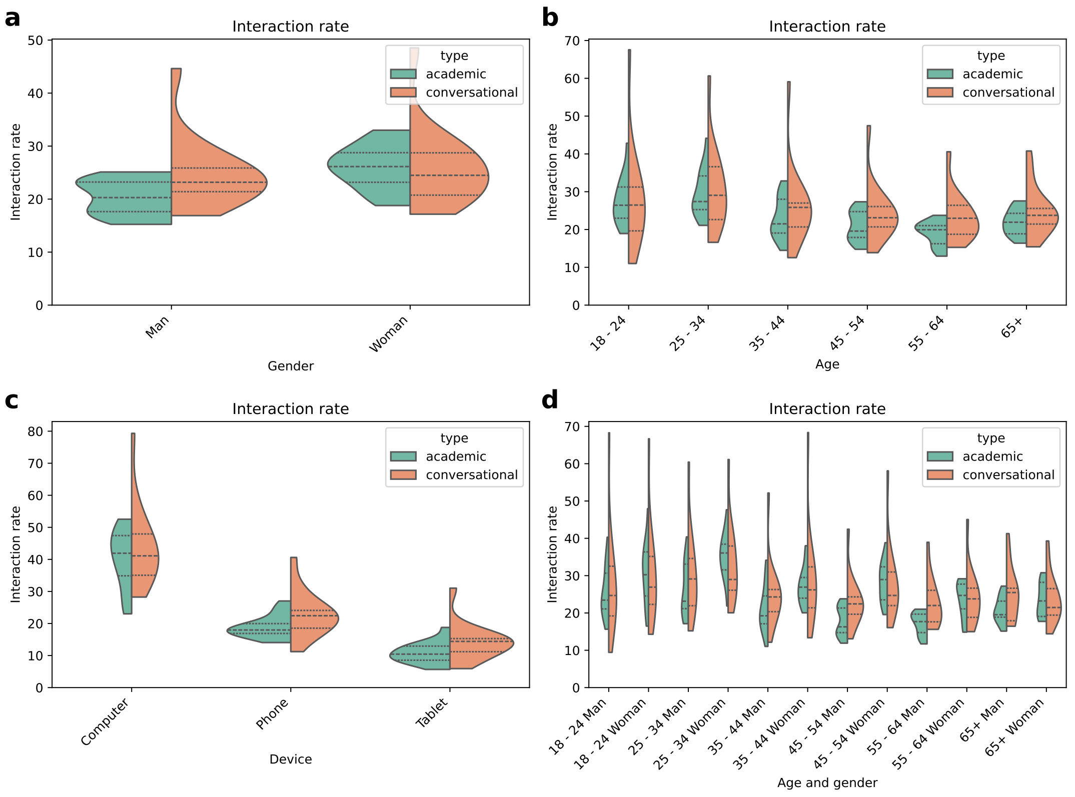

Figure S14: **Interaction rate by audience profile.** Distribution of interaction rate by (**a**) gender , (**b**) age (**c**) device and (**d**) the combination of age and gender.

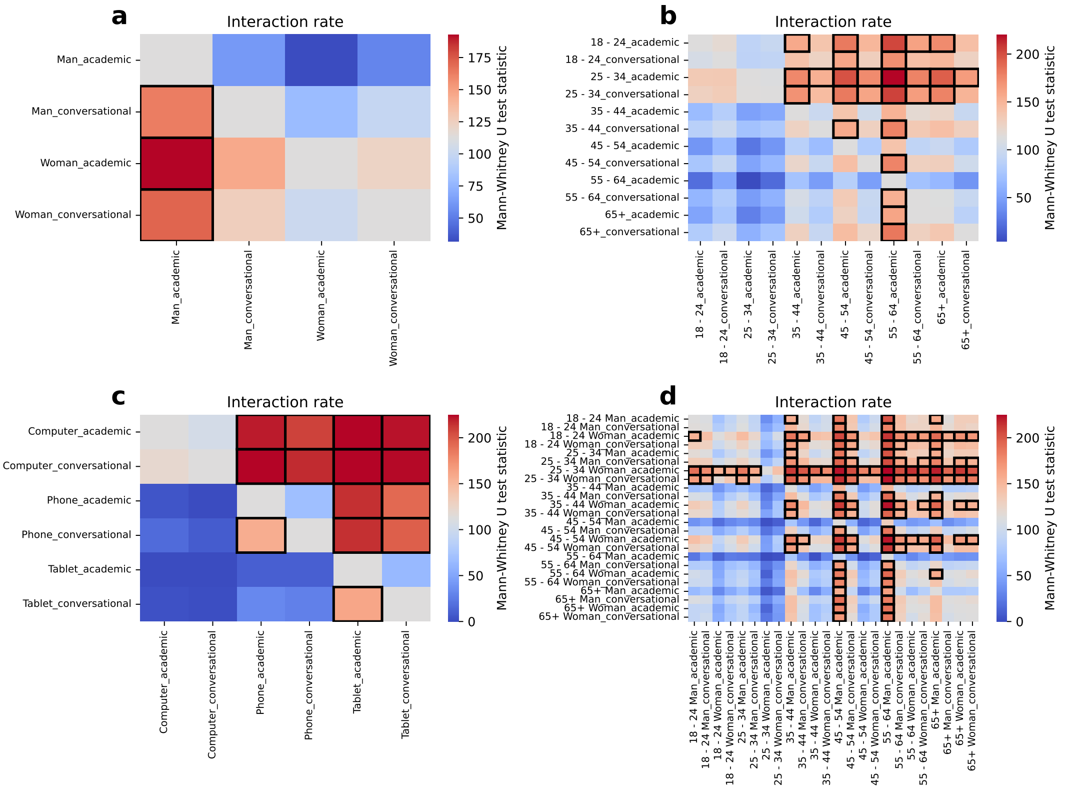

Figure S15: **Statistical tests between the interaction rate by audience profile.** Results of the statistical tests between the distributions of the interaction rate by (**a**) gender , (**b**) age (**c**) device, and (**d**) the combination of age and gender. Entries marked in black show statistically significant tests (p-value<0.05).

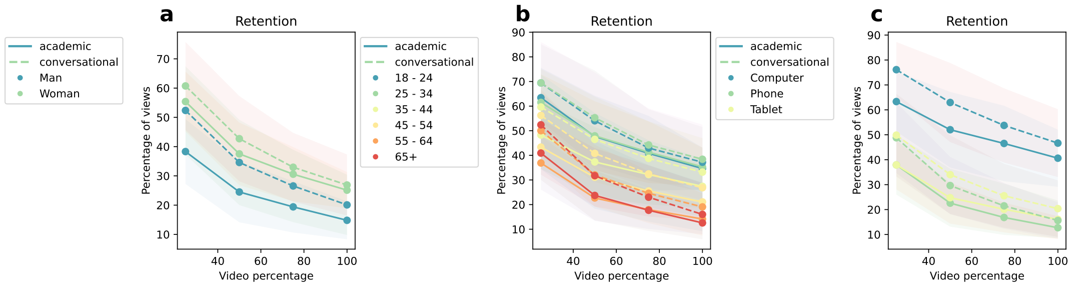

Figure S16: **Video retention based on audience profile.** Percentage of users viewing 25%, 50%, 75% and 100% of content by (**a**) gender , (**b**) age and (**c**) device.
