# Supplementary figures and images for: An analytical and experimental study of the energy transition discourse on YouTube
Source: PLoS One. 2026 Jul 15;21(7):e0352691. doi: 10.1371/journal.pone.0352691 (PMC13372142; doi:10.1371/journal.pone.0352691)

Number of Videos per Year

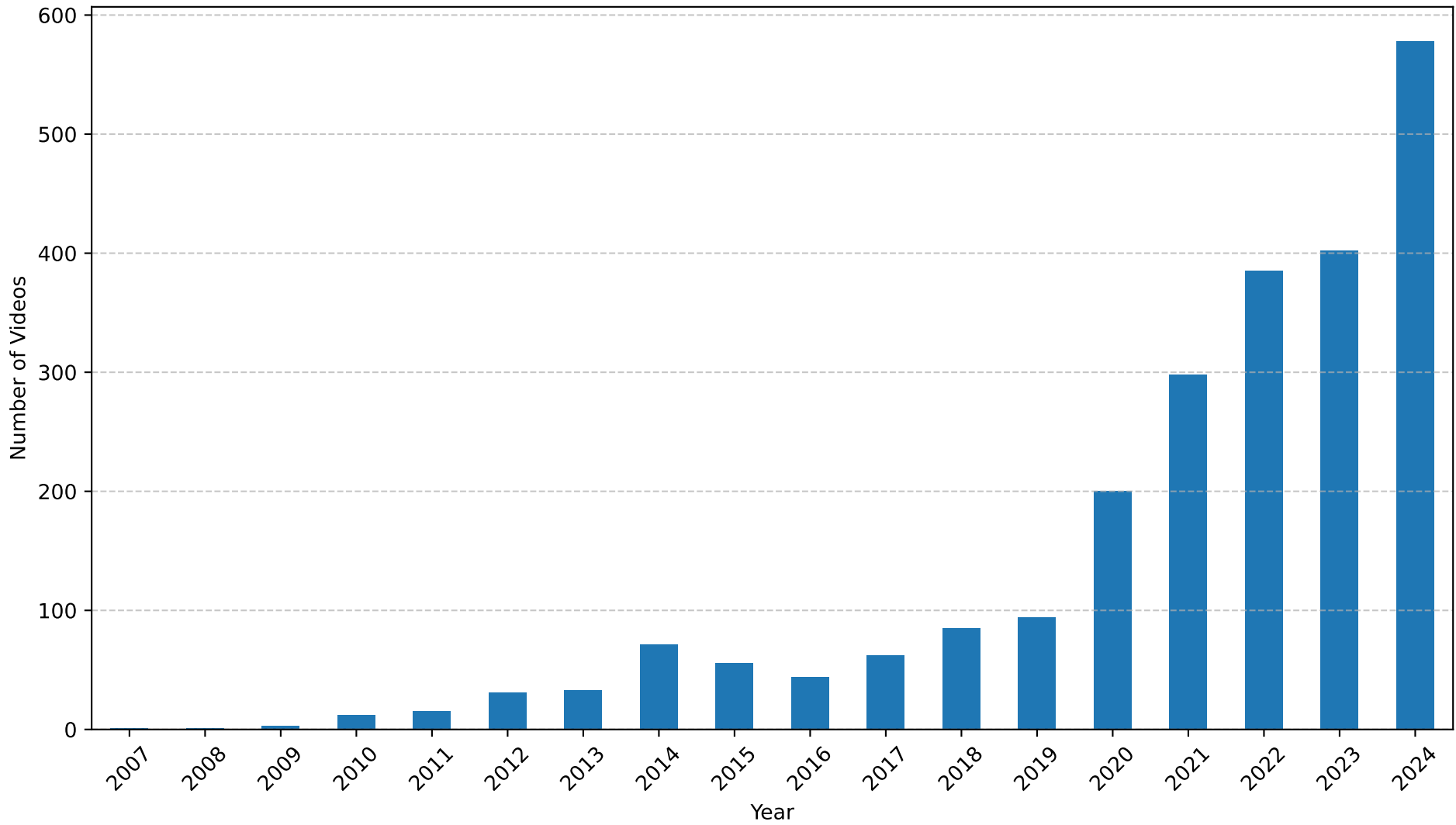

Supplement: S1 Fig — (PDF) [file pone.0352691.s002.pdf]

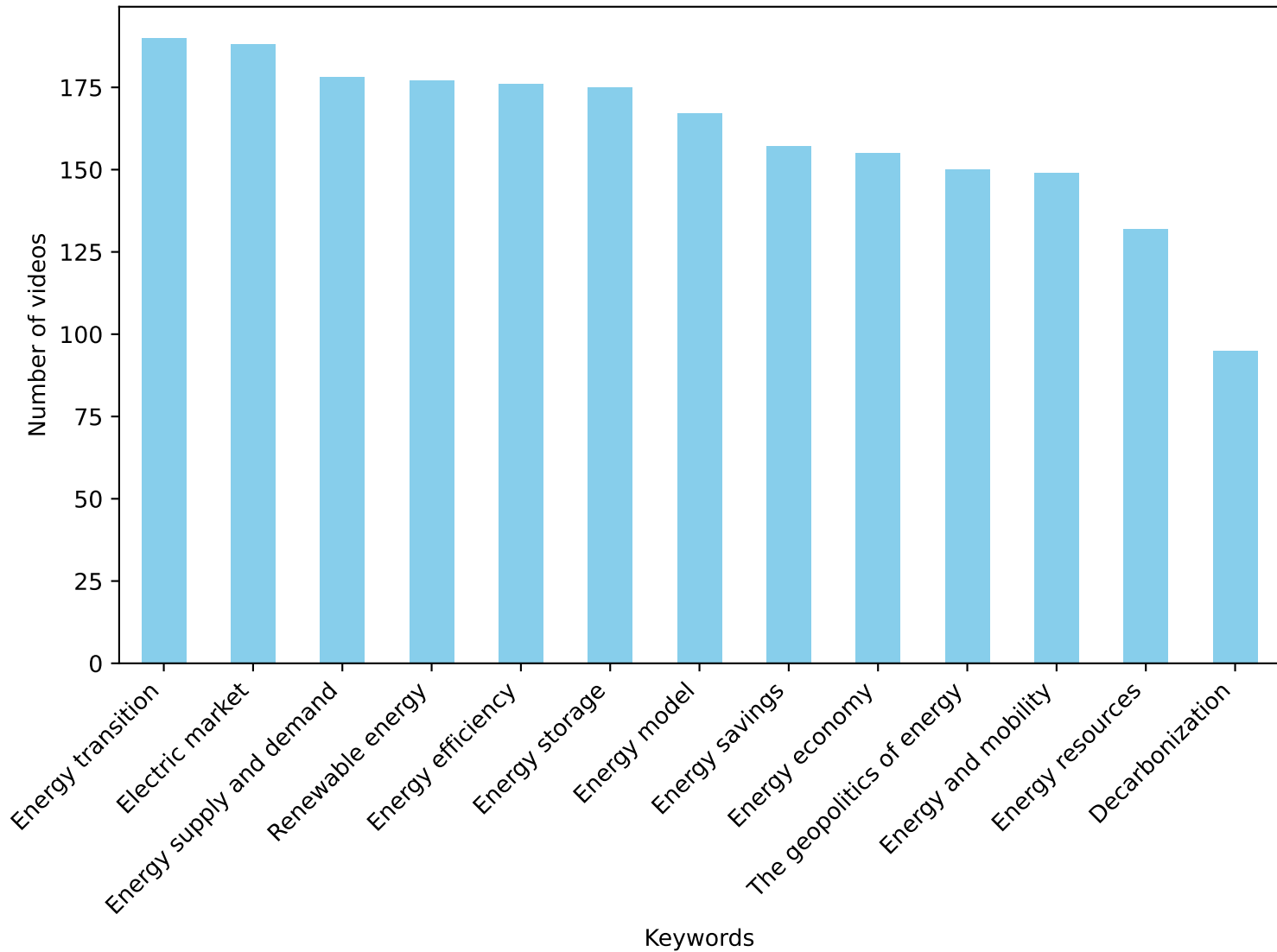

Supplement: S2 Fig — (PDF) [file pone.0352691.s003.pdf]

**a**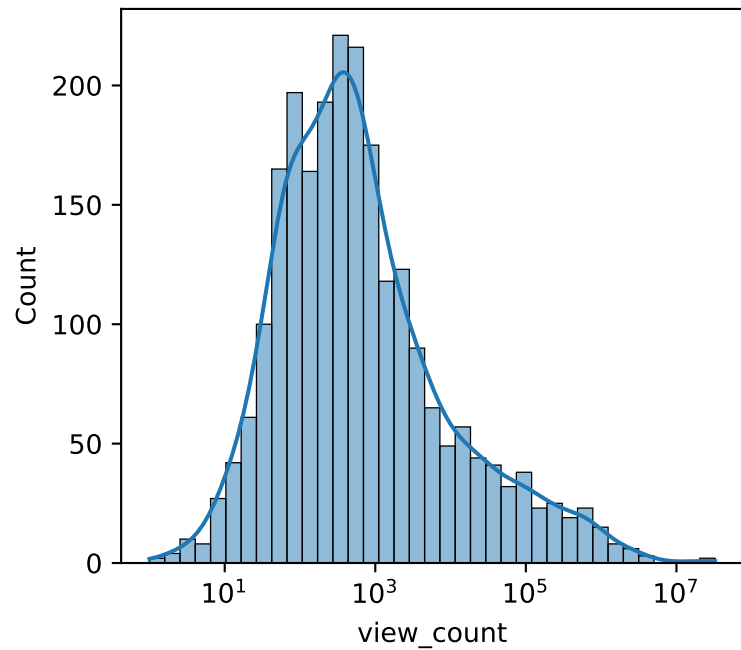**b**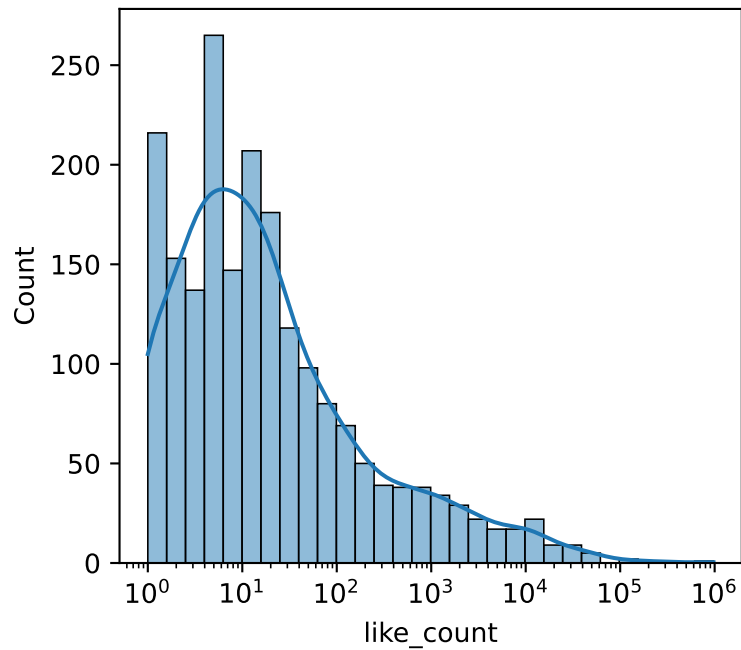**c**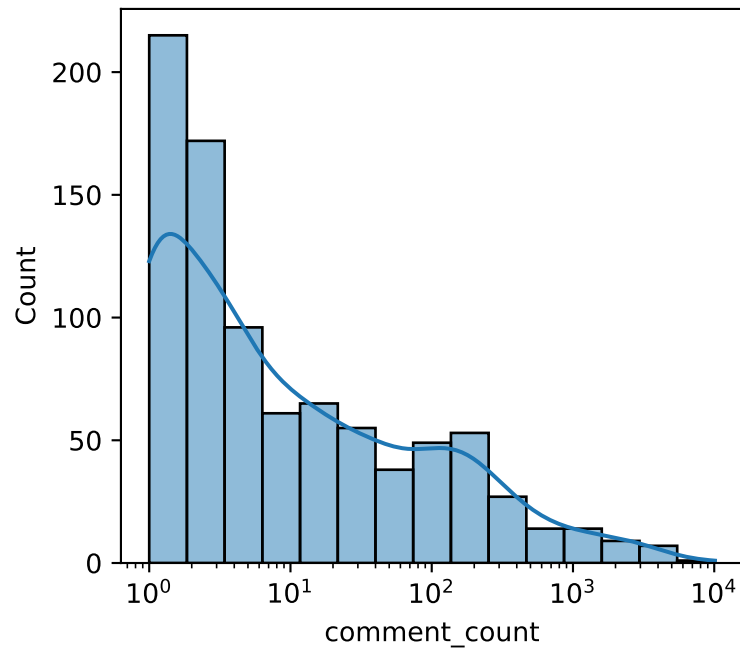

Supplement: S3 Fig — (PDF) [file pone.0352691.s004.pdf]

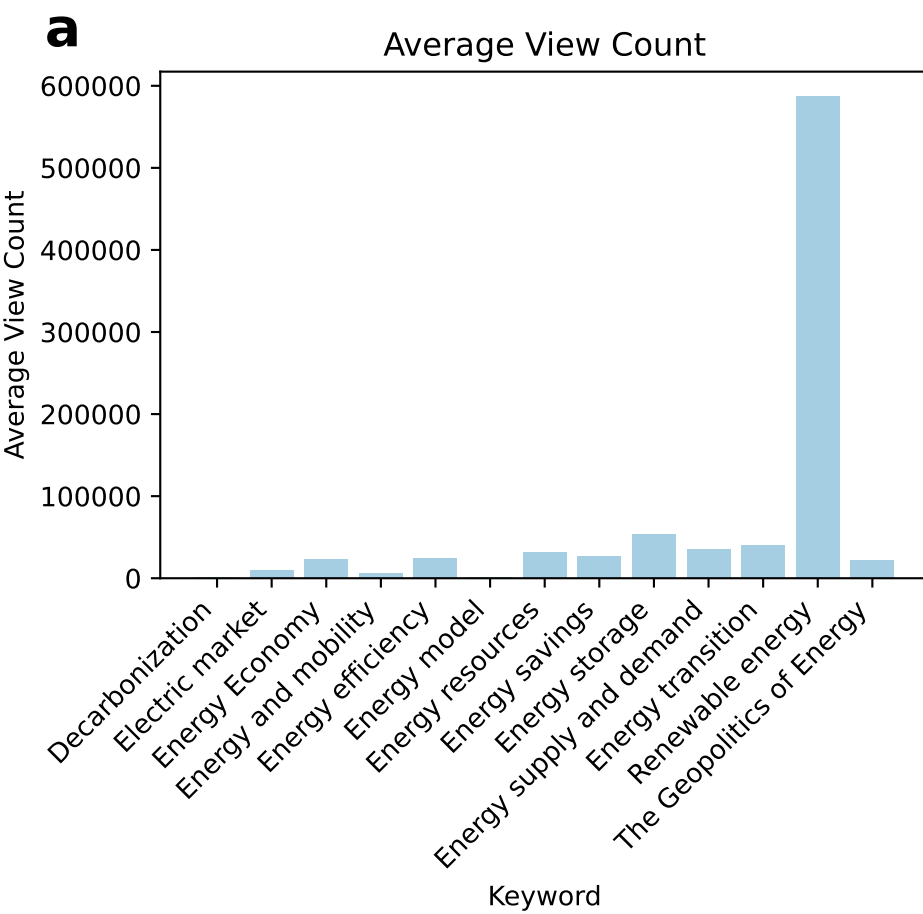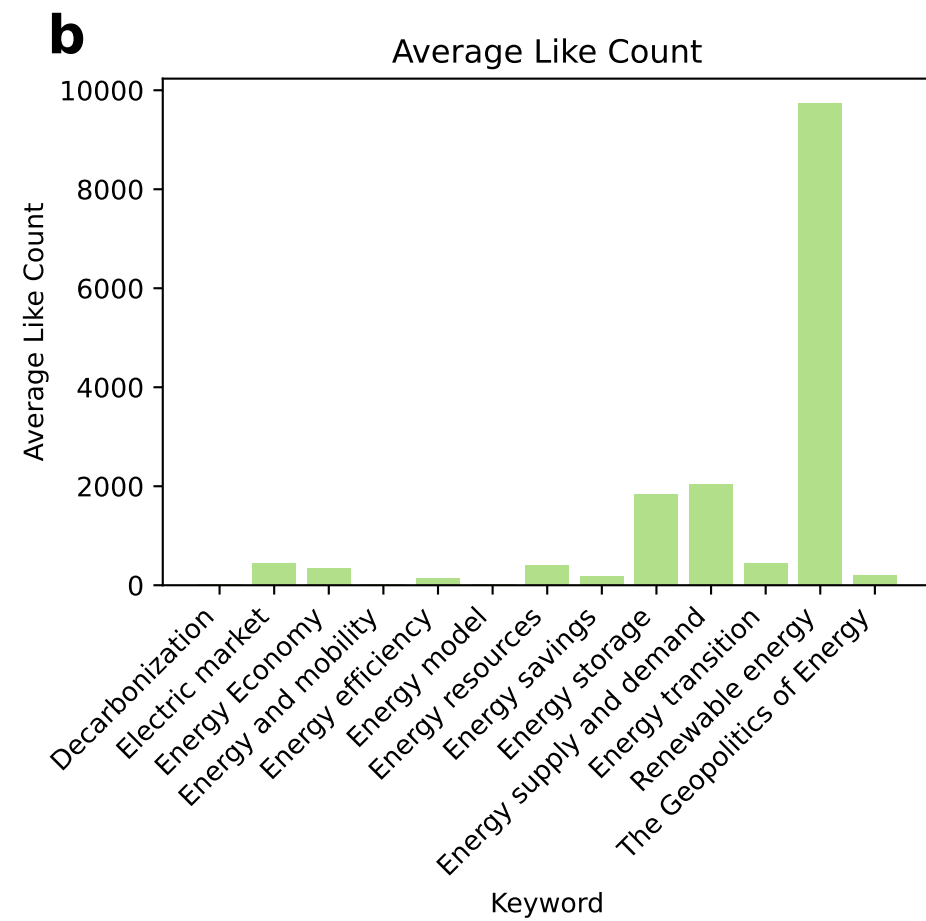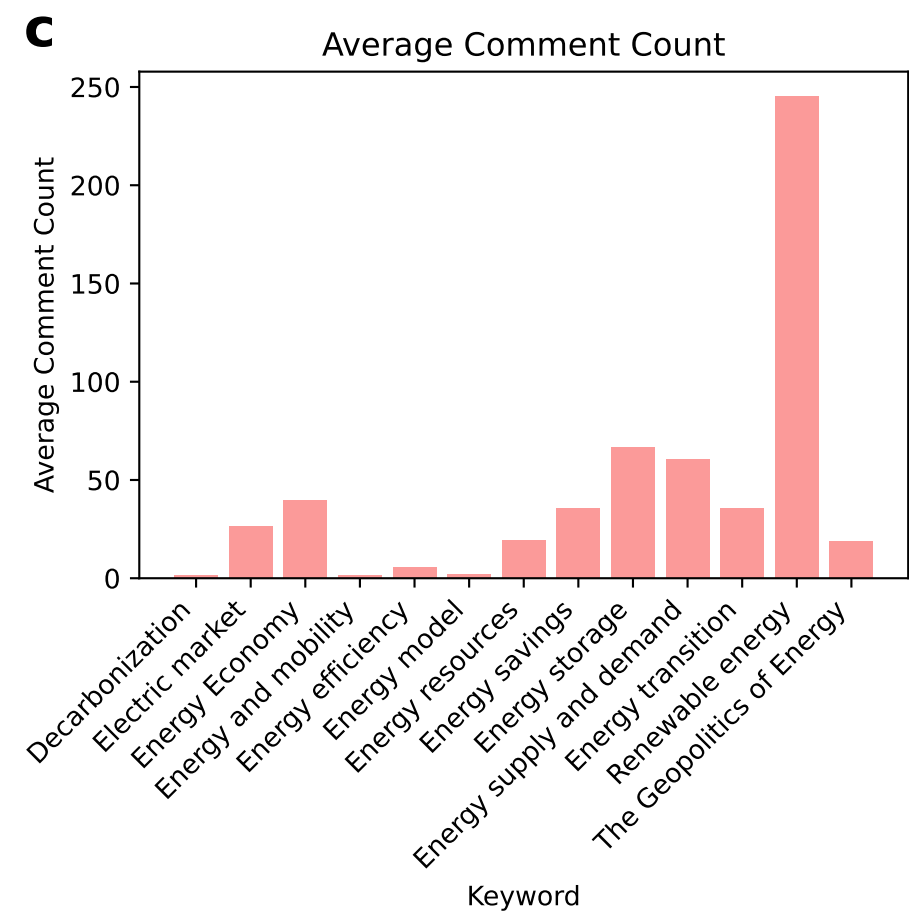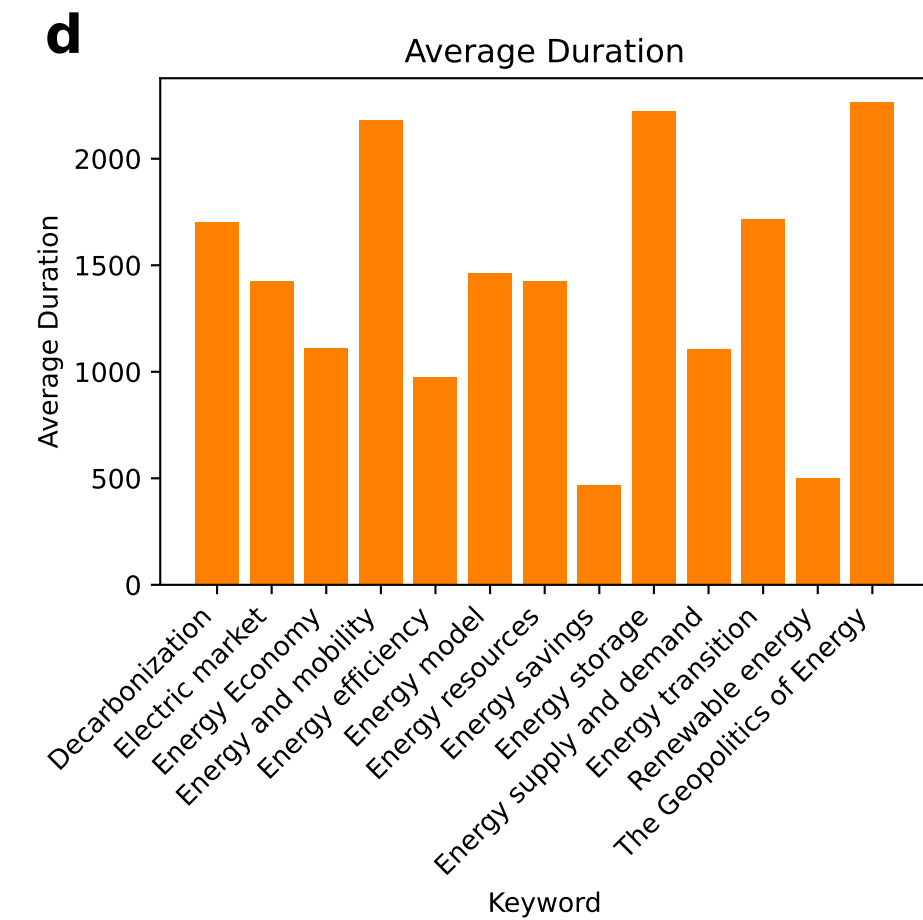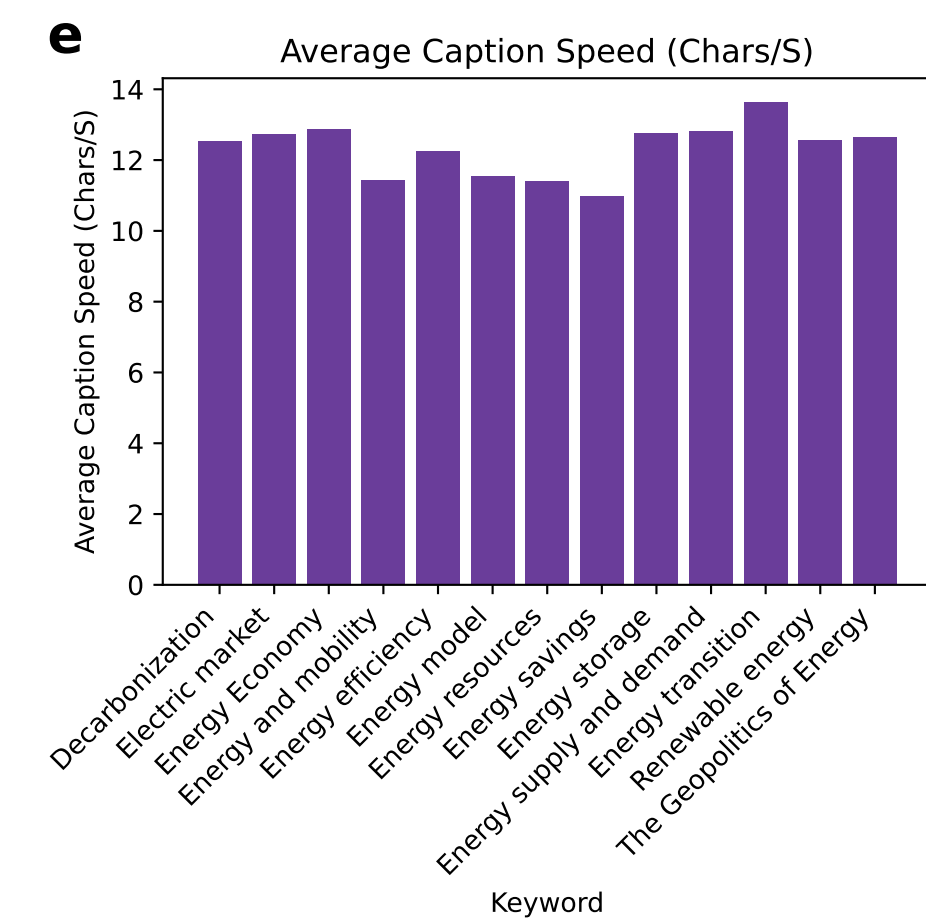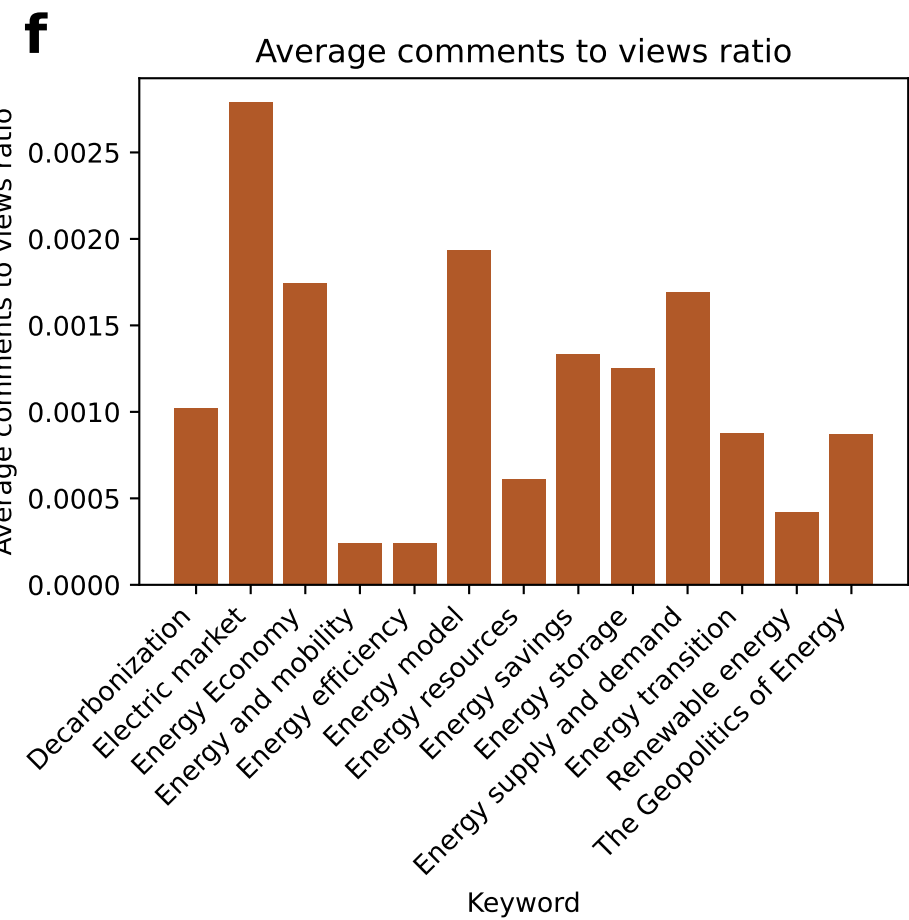

Supplement: S4 Fig — (PDF) [file pone.0352691.s005.pdf]

Mann-Whitney U Test Statistics for Positive  
(Row vs Column, \*p < 0.05)

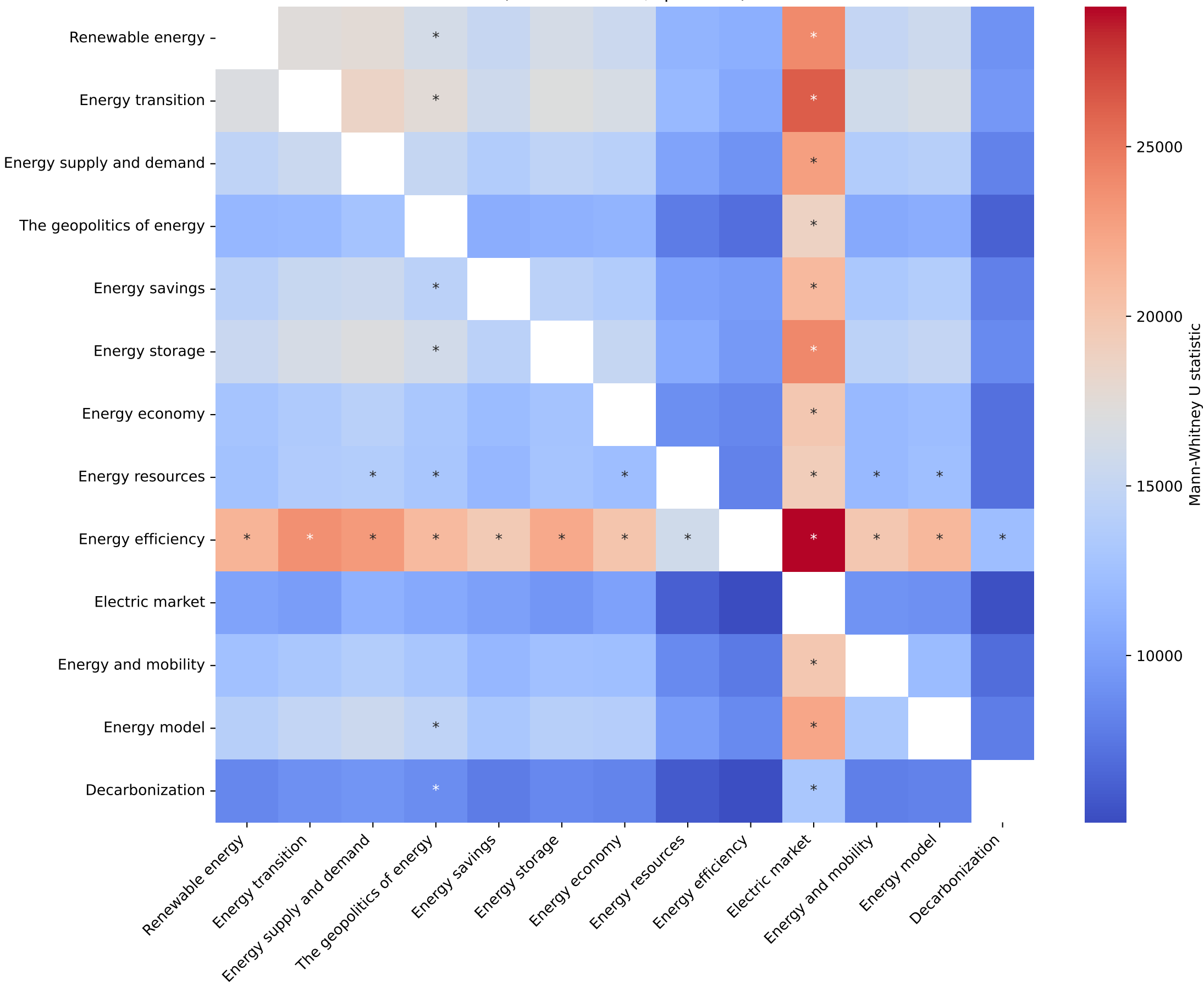

Supplement: S5 Fig — (PDF) [file pone.0352691.s006.pdf]

Mann-Whitney U Test Statistics for Negative  
(Row vs Column, \*p < 0.05)

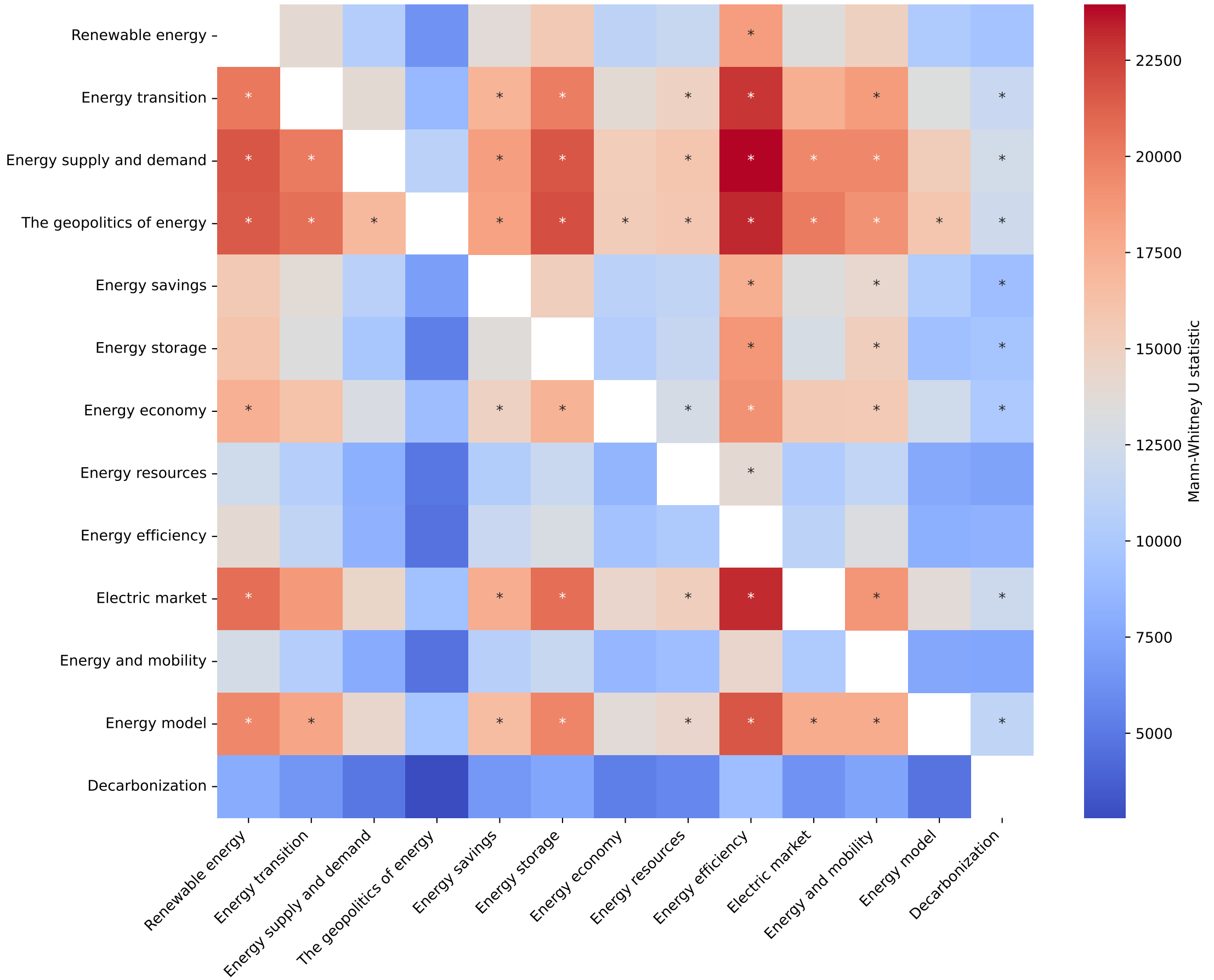

Supplement: S6 Fig — (PDF) [file pone.0352691.s007.pdf]

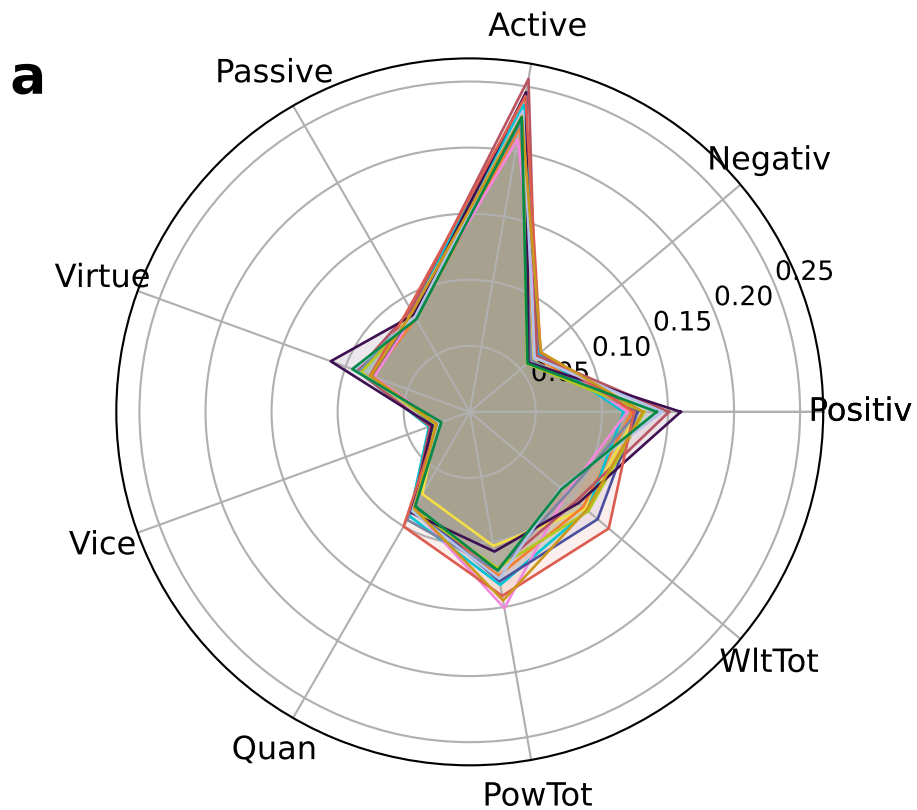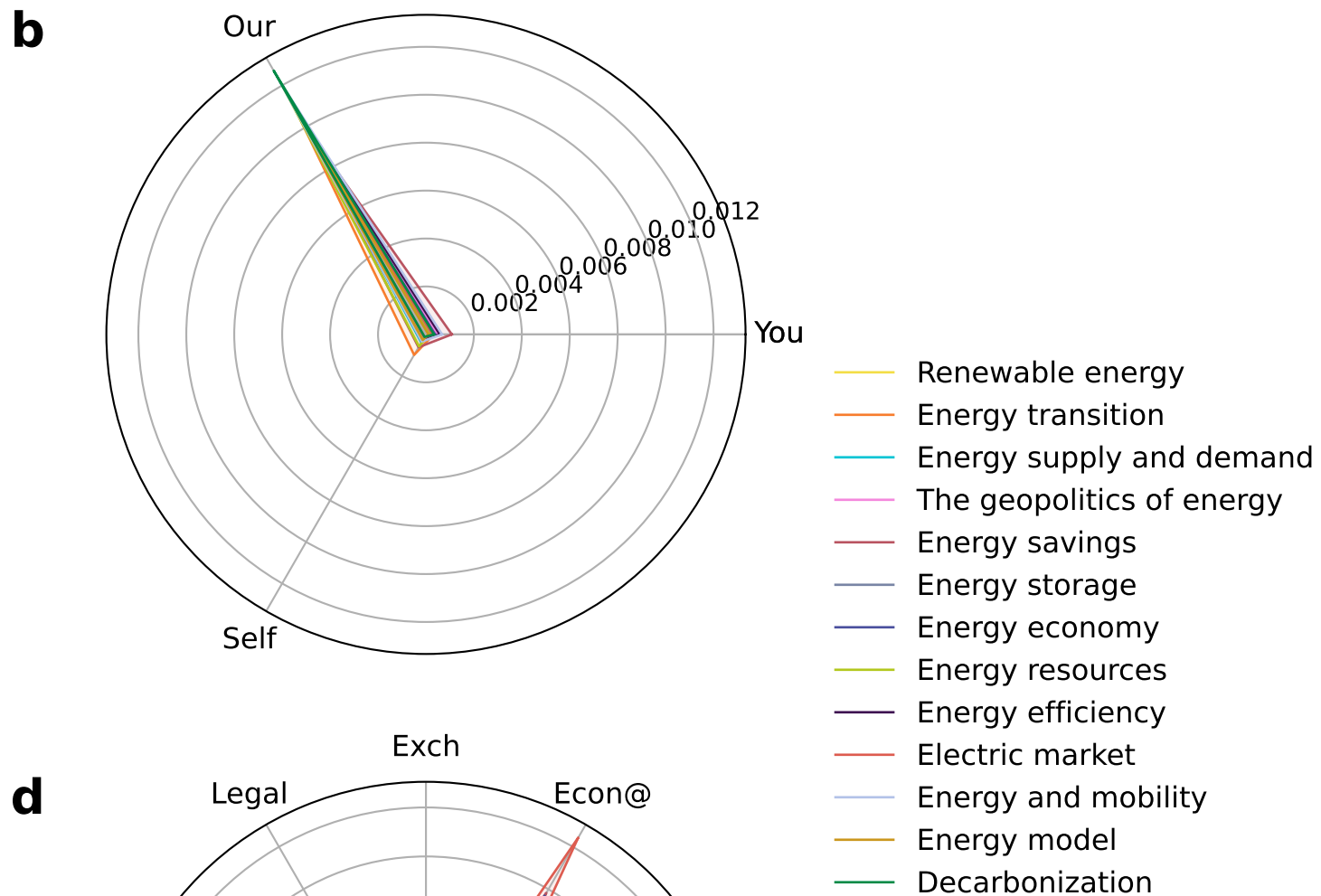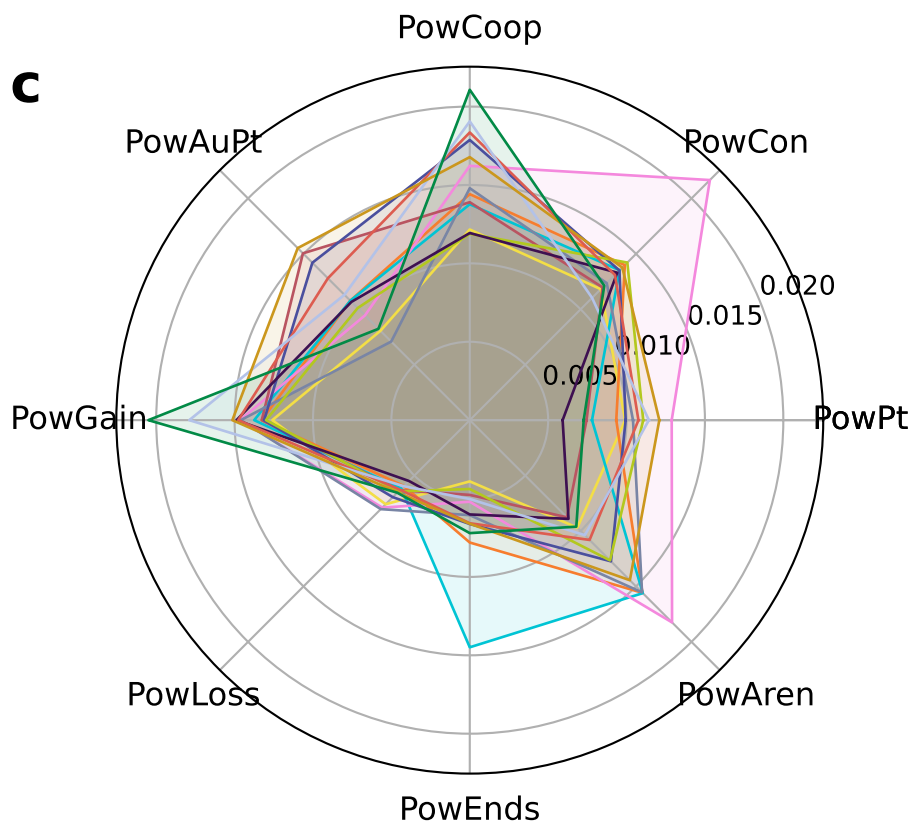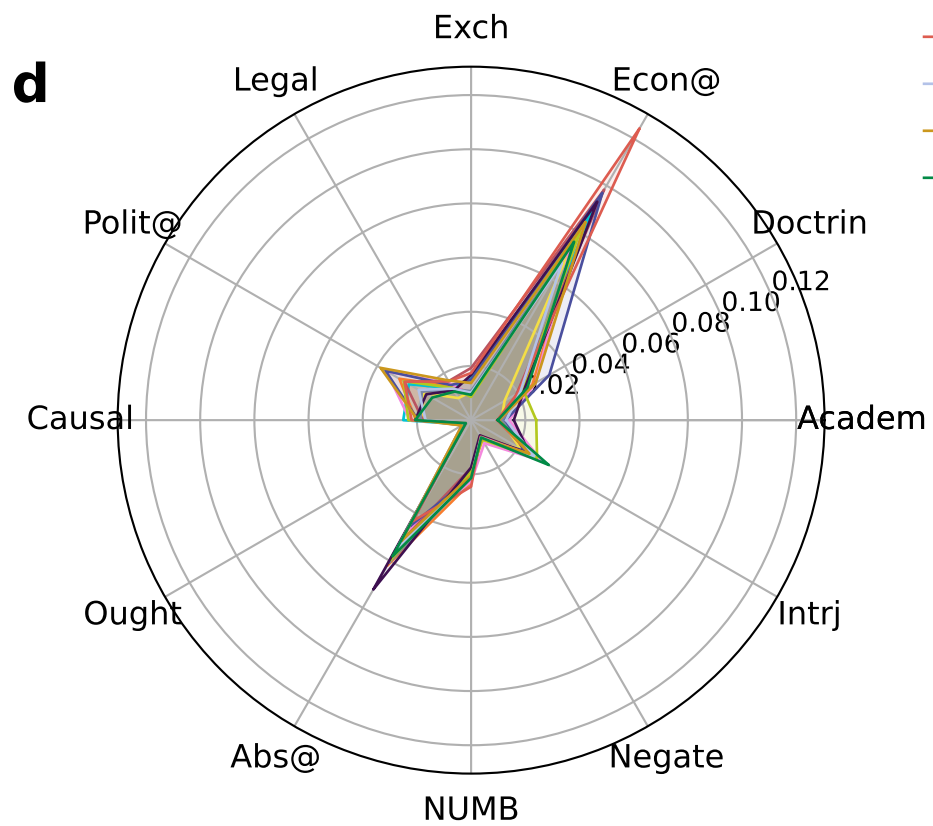

Supplement: S7 Fig — (PDF) [file pone.0352691.s008.pdf]

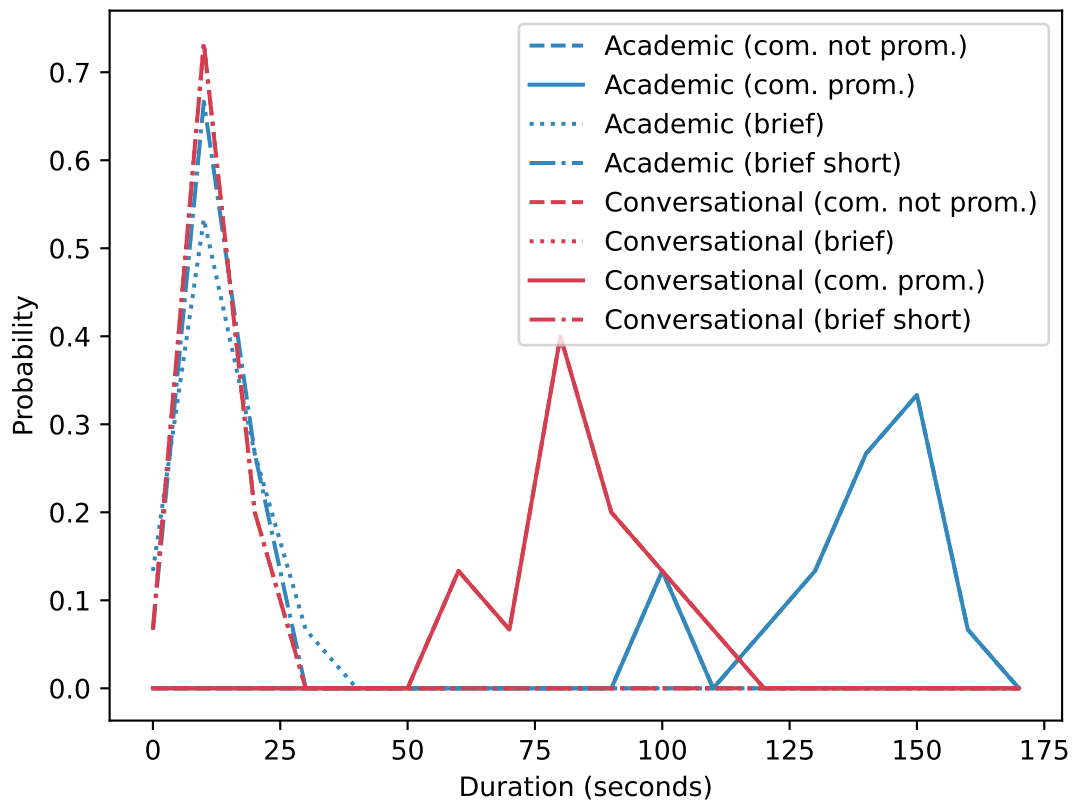

Supplement: S8 Fig — (PDF) [file pone.0352691.s009.pdf]

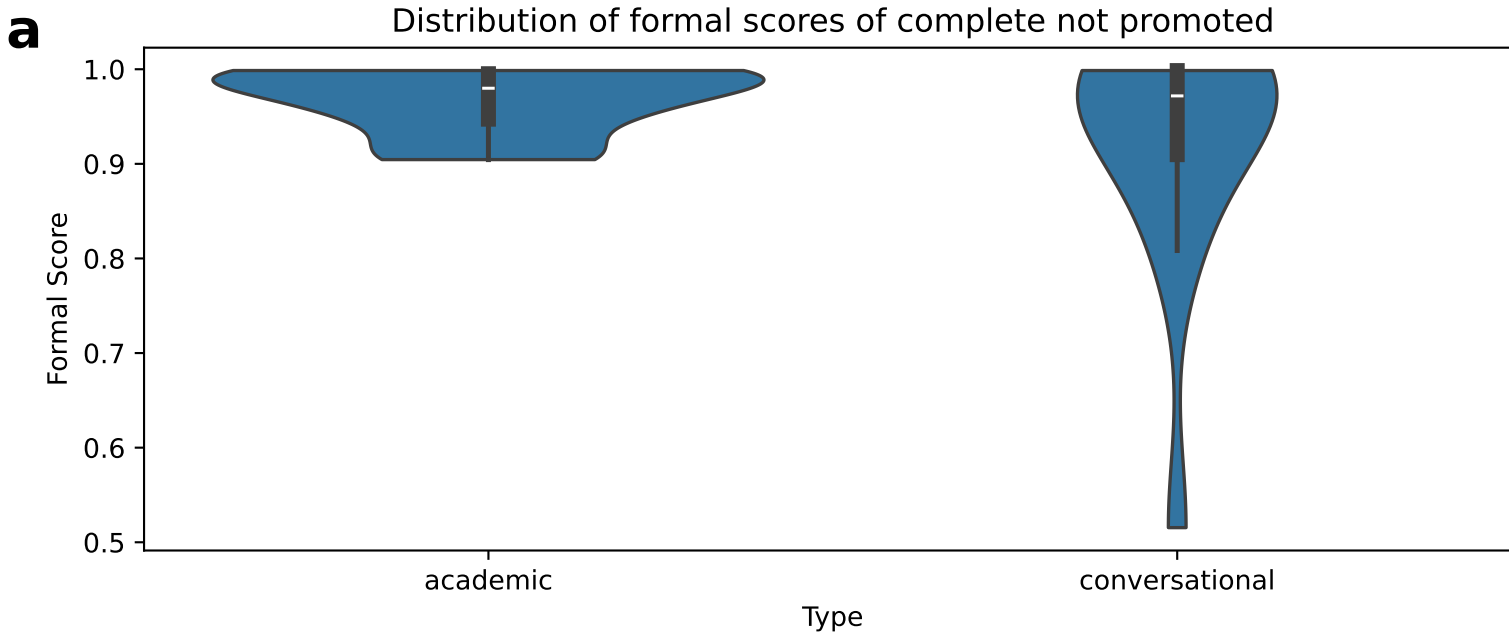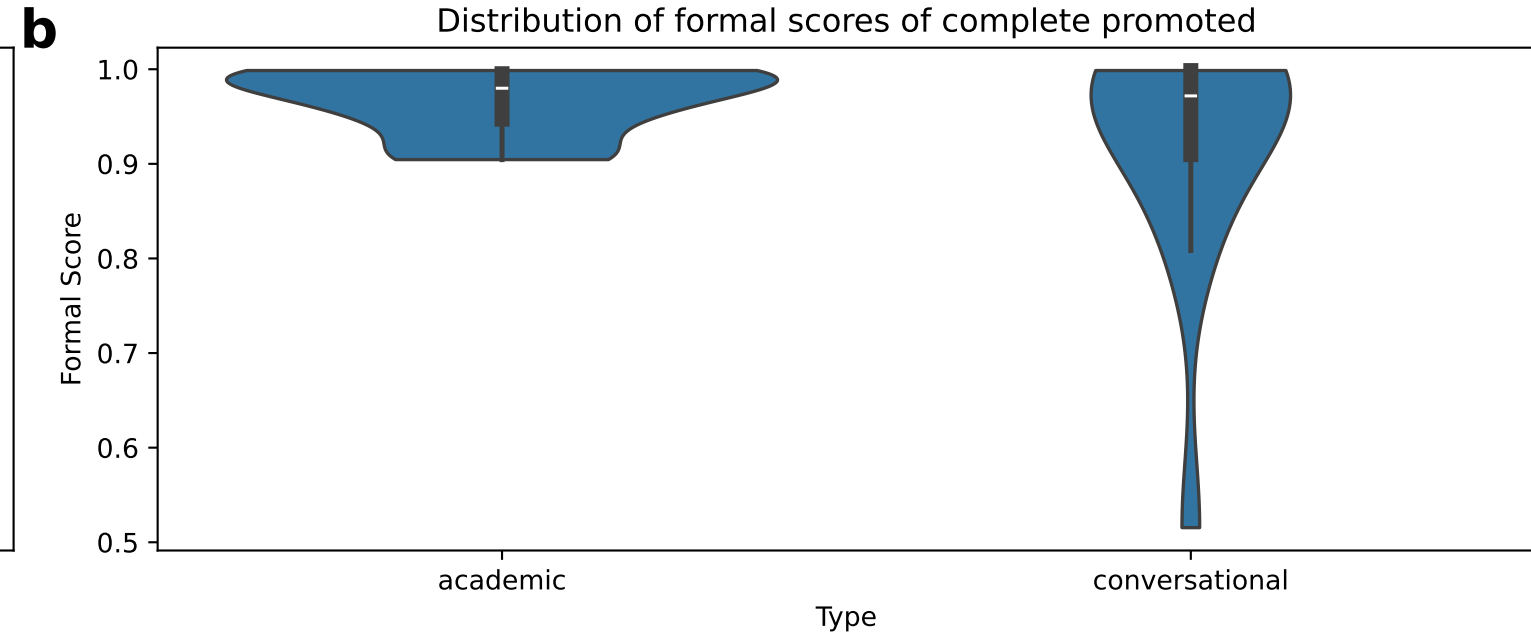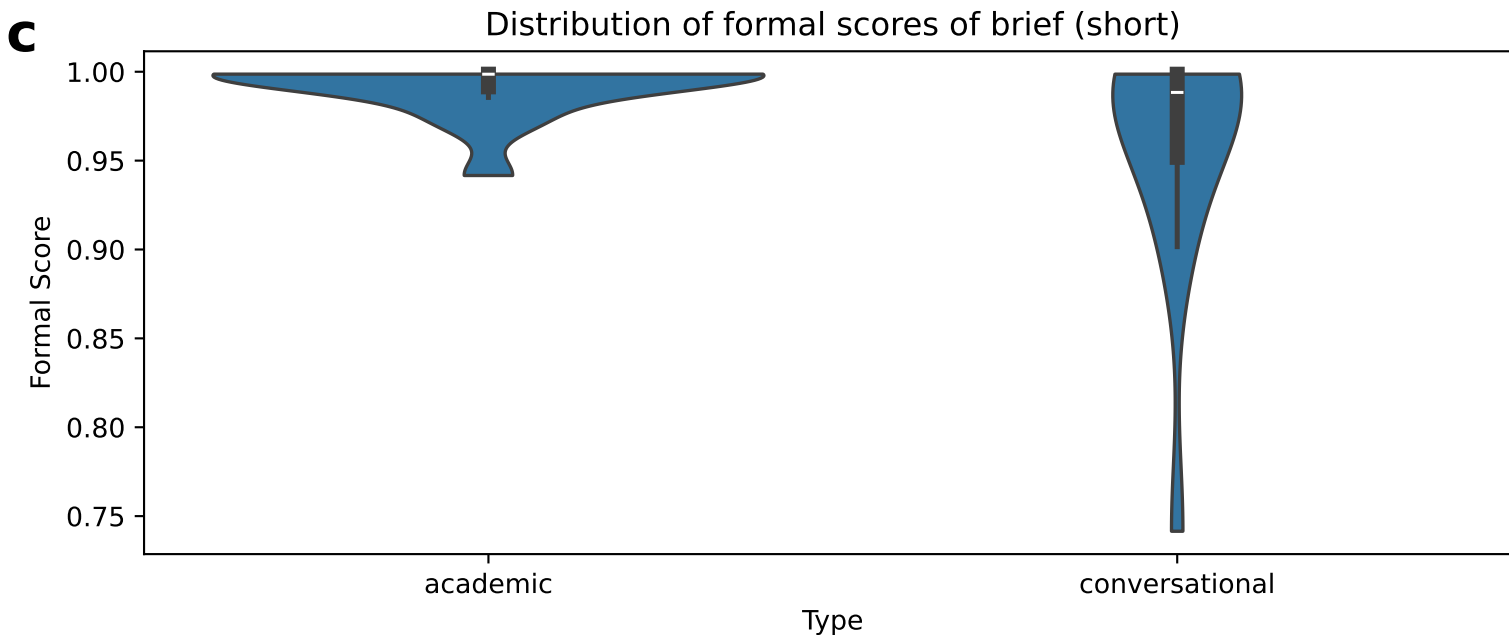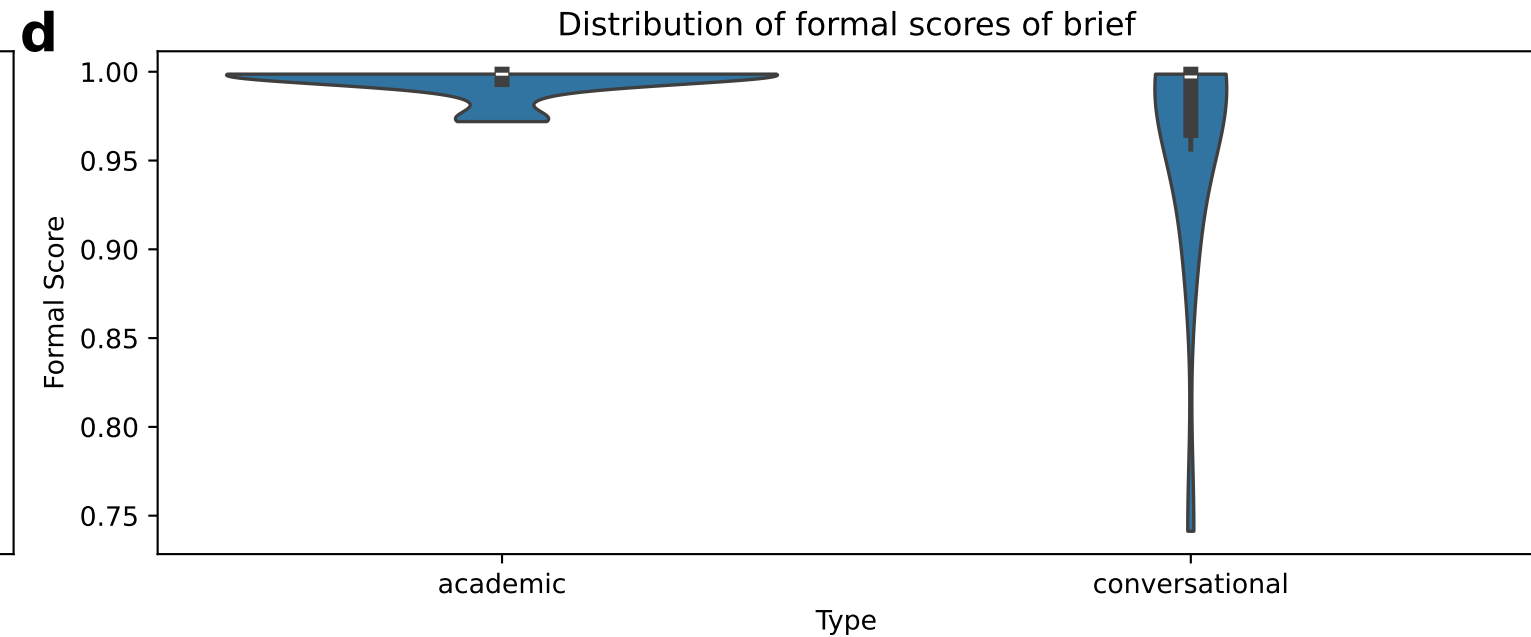

Supplement: S9 Fig — (PDF) [file pone.0352691.s010.pdf]

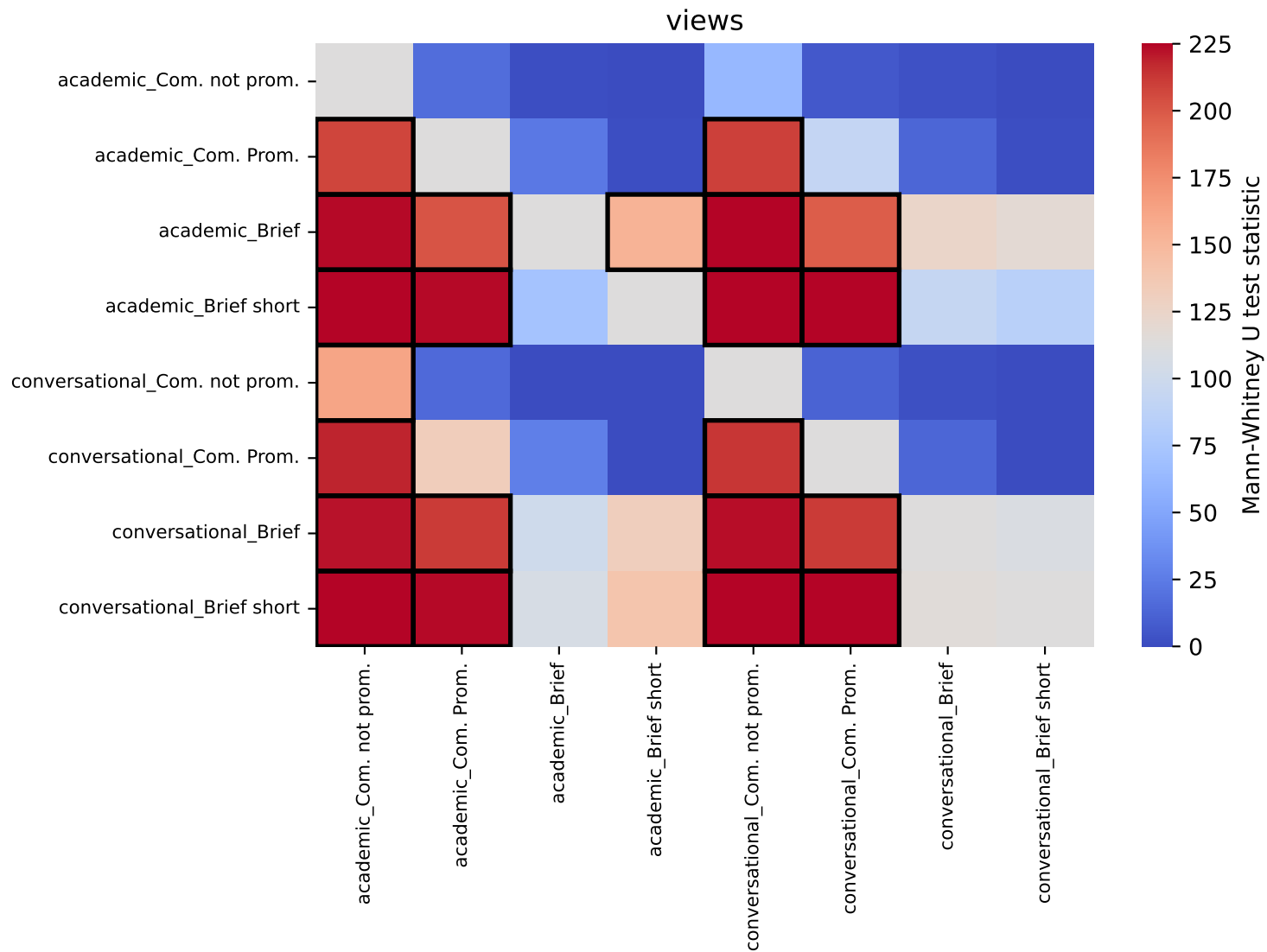

Supplement: S10 Fig — (PDF) [file pone.0352691.s011.pdf]

Relative retention performance (Com. Prom.)

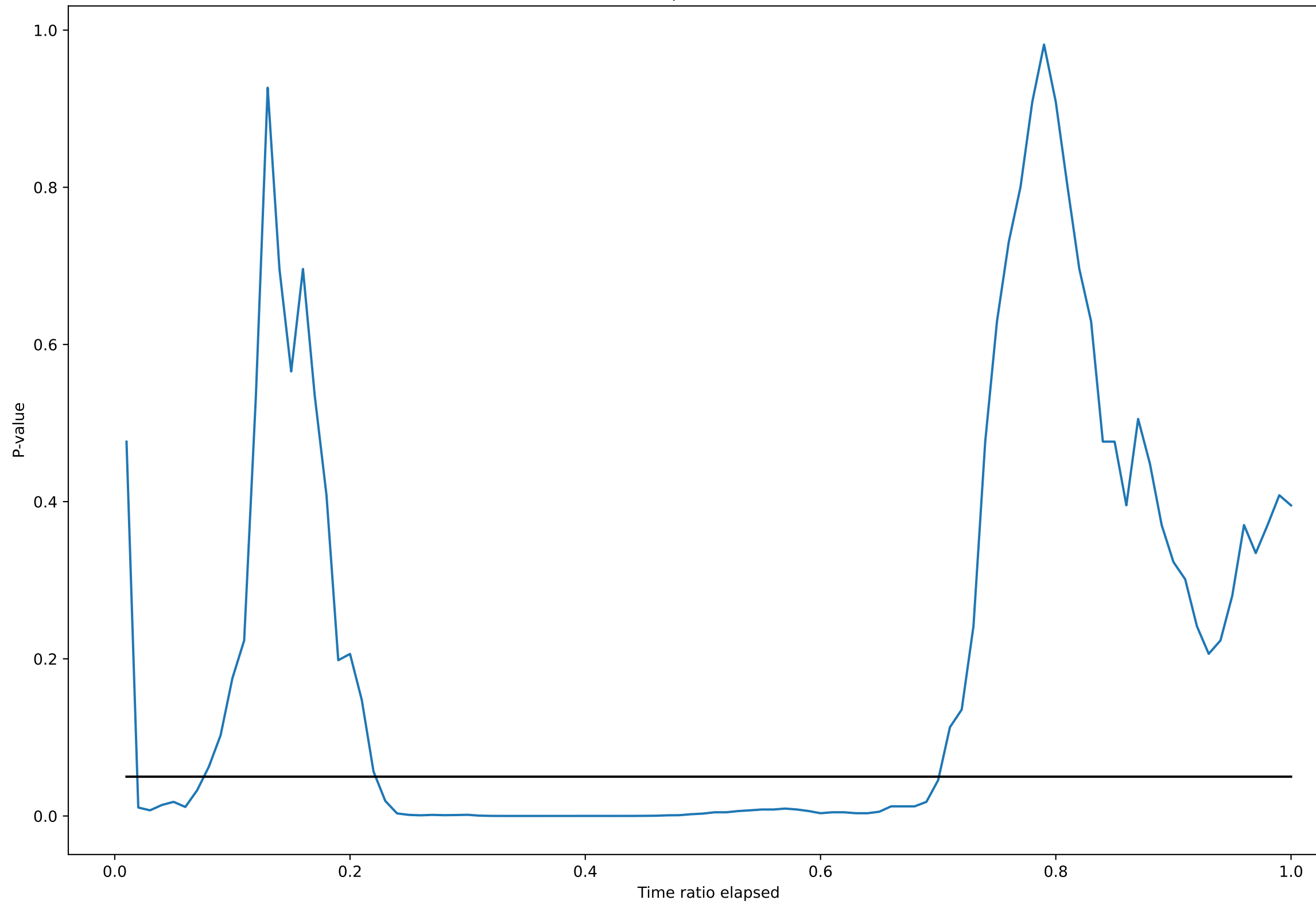

Supplement: S11 Fig — (PDF) [file pone.0352691.s012.pdf]

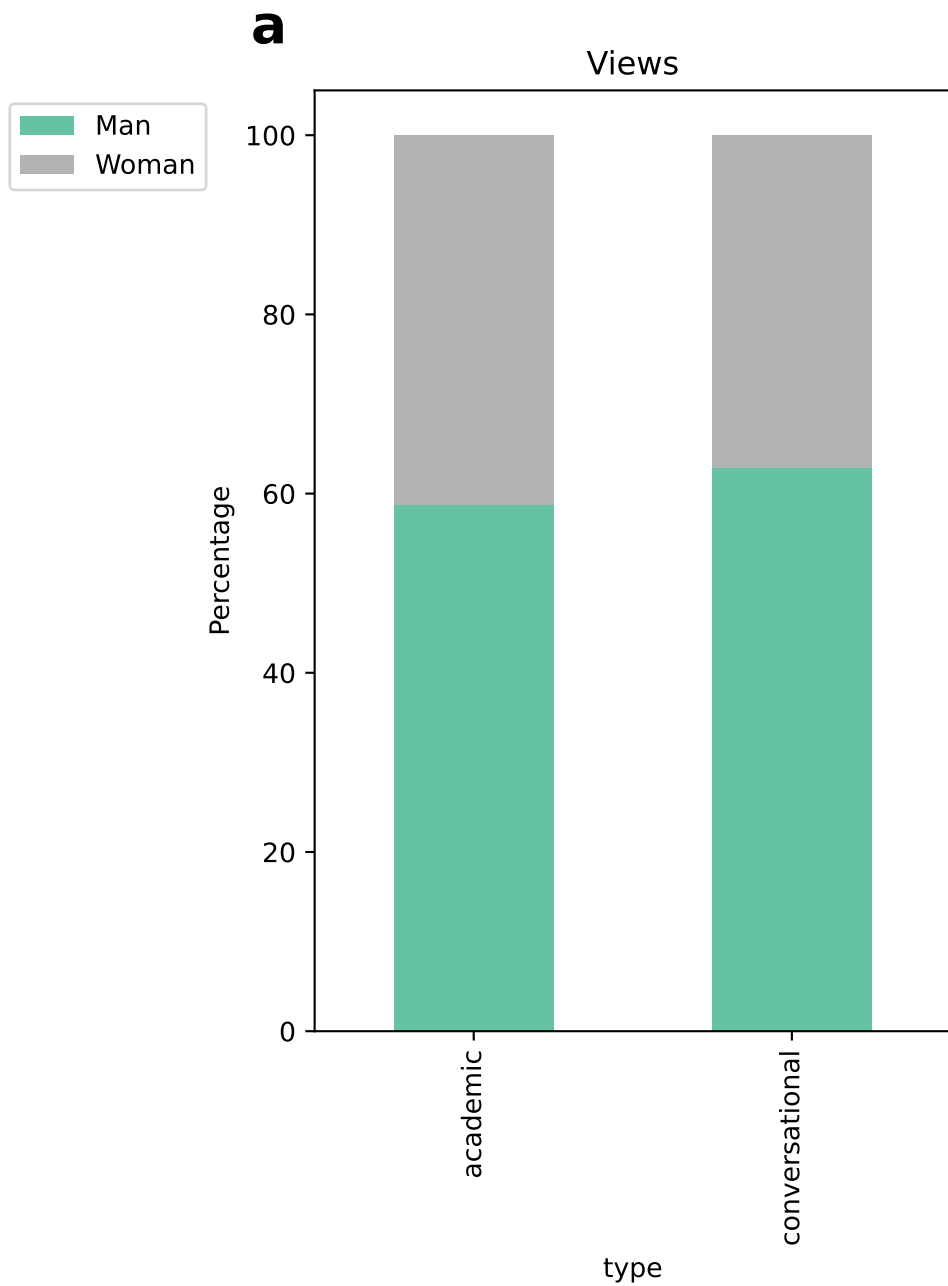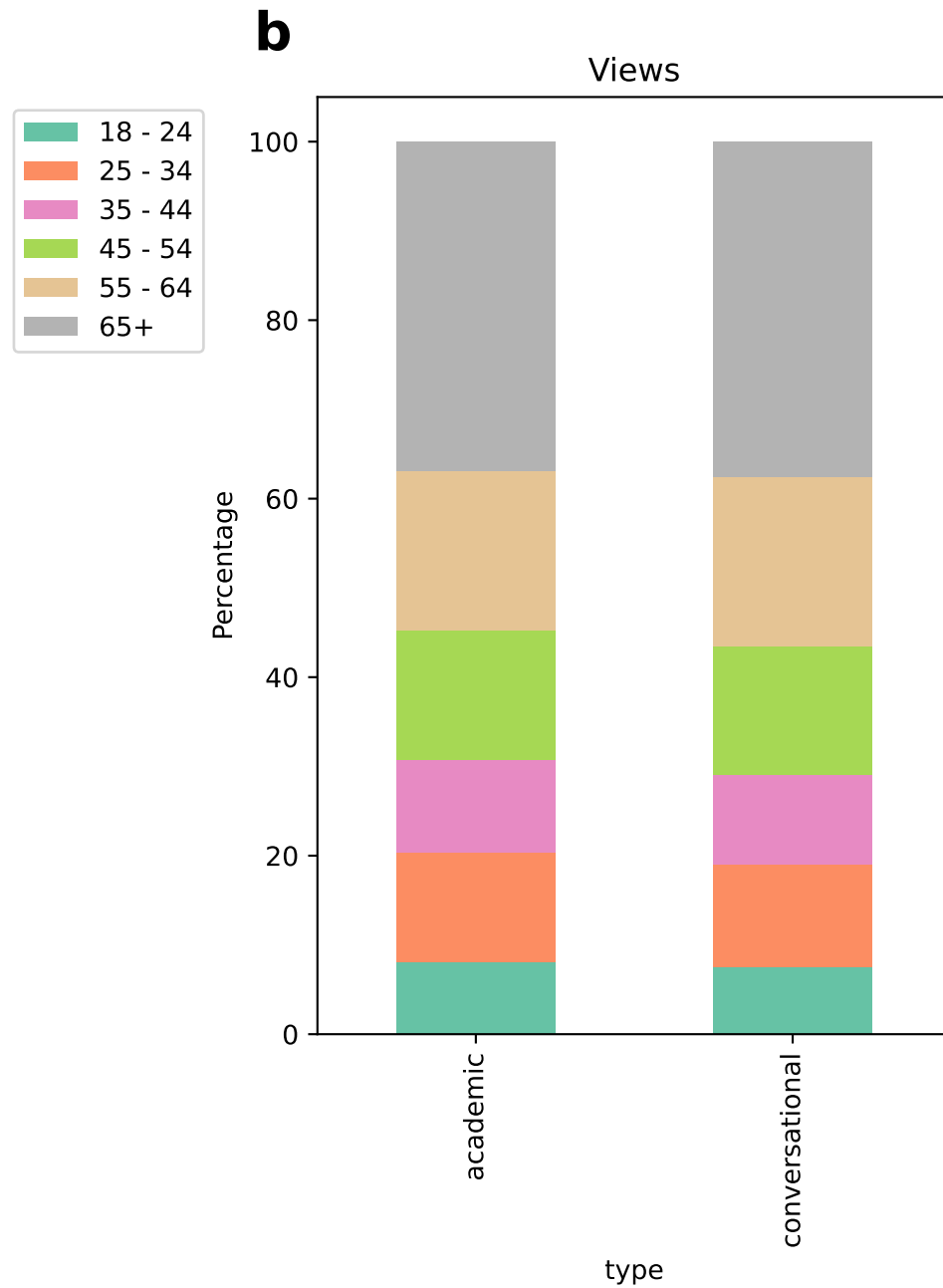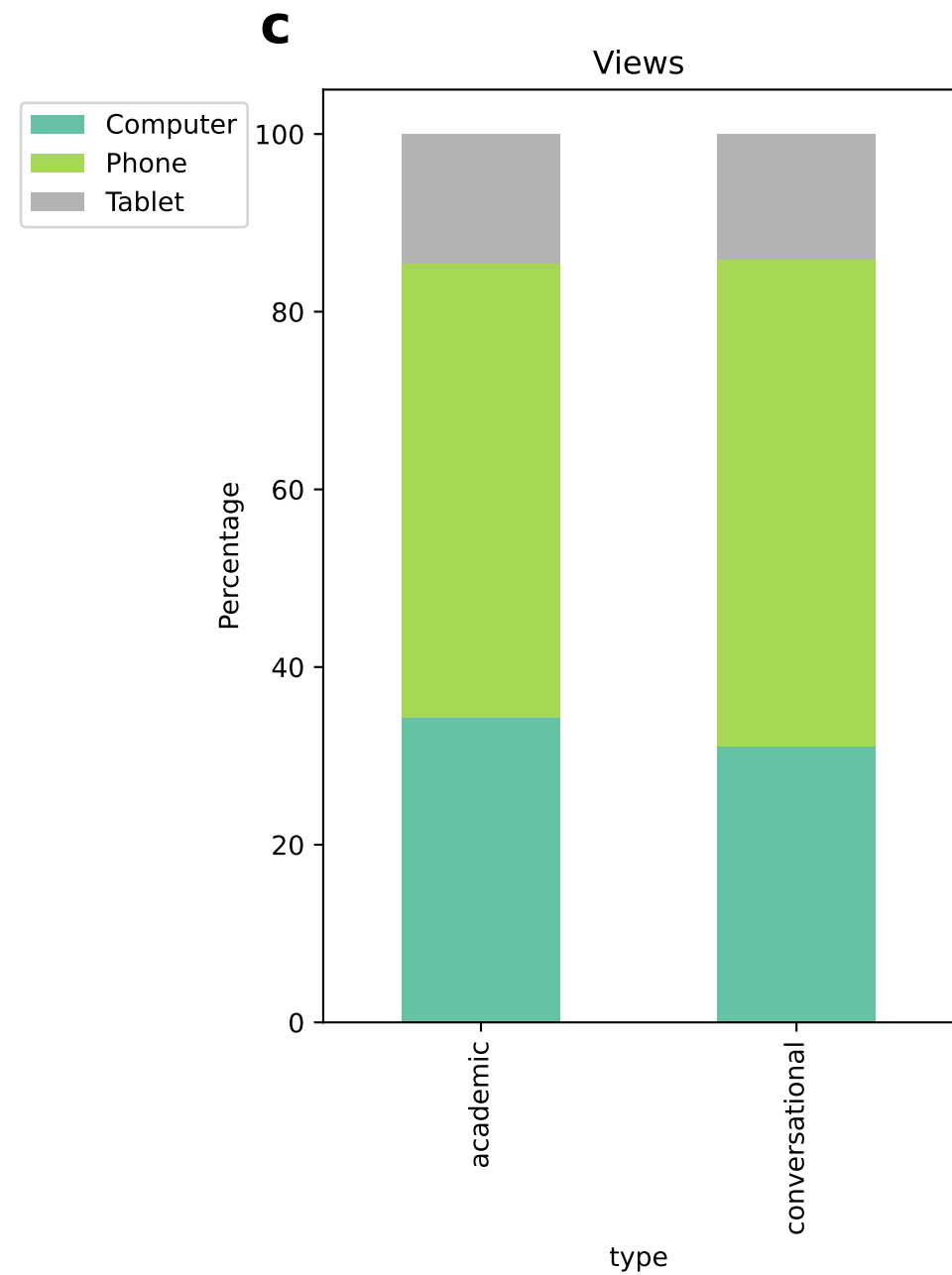

Supplement: S12 Fig — (PDF) [file pone.0352691.s013.pdf]

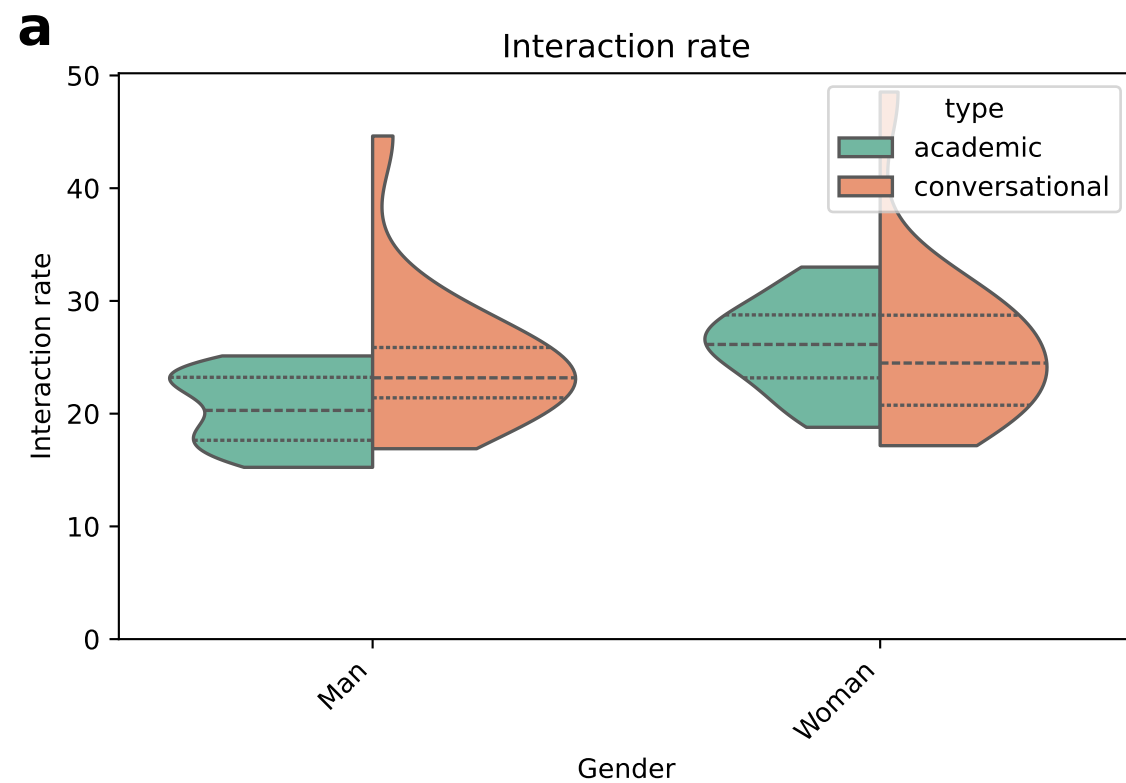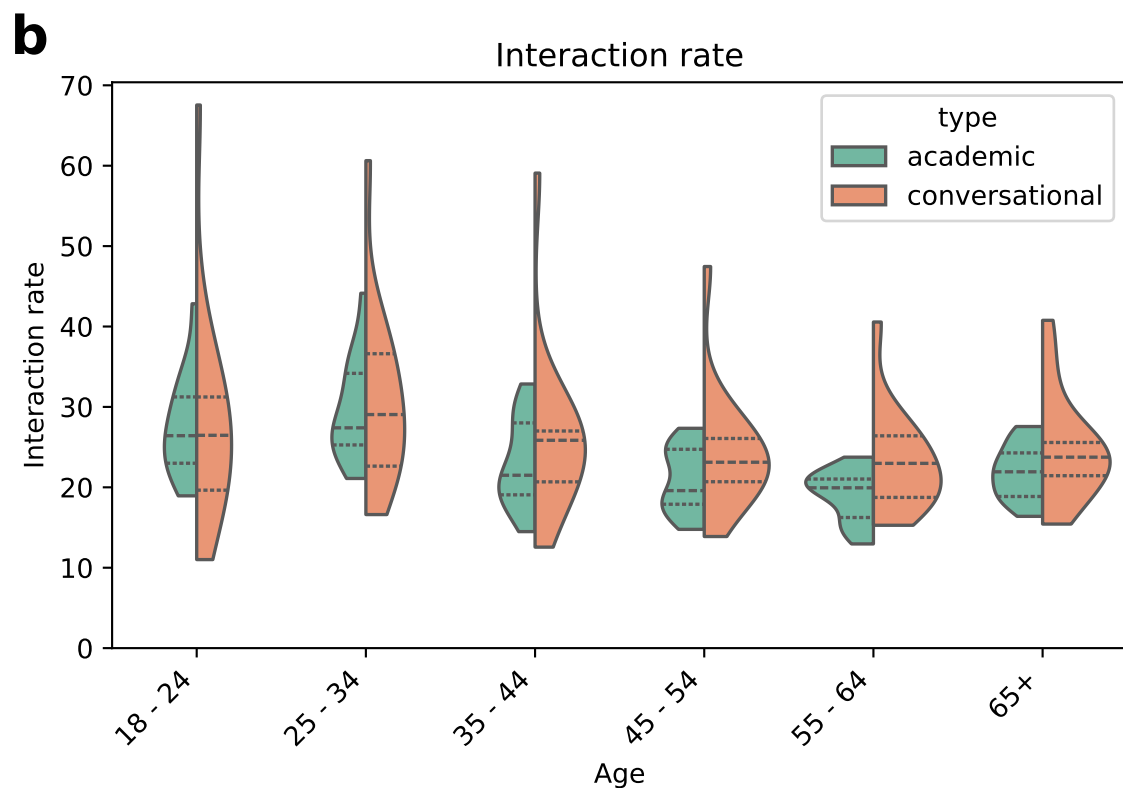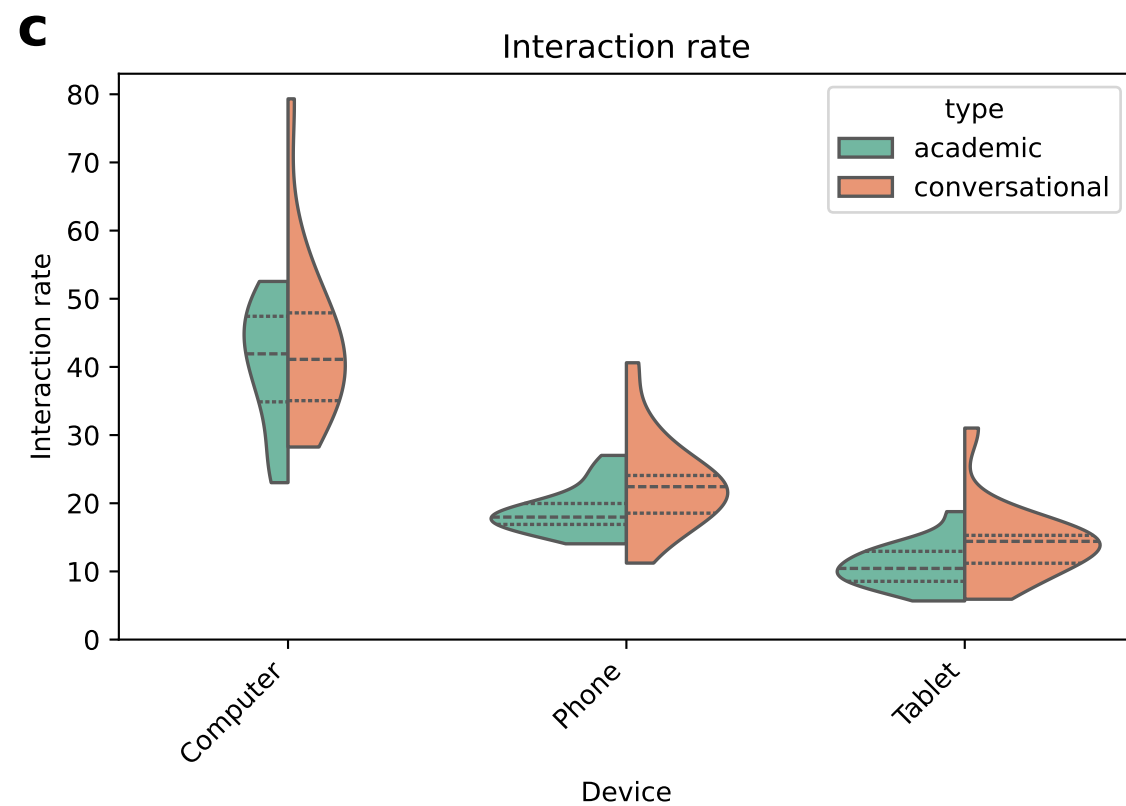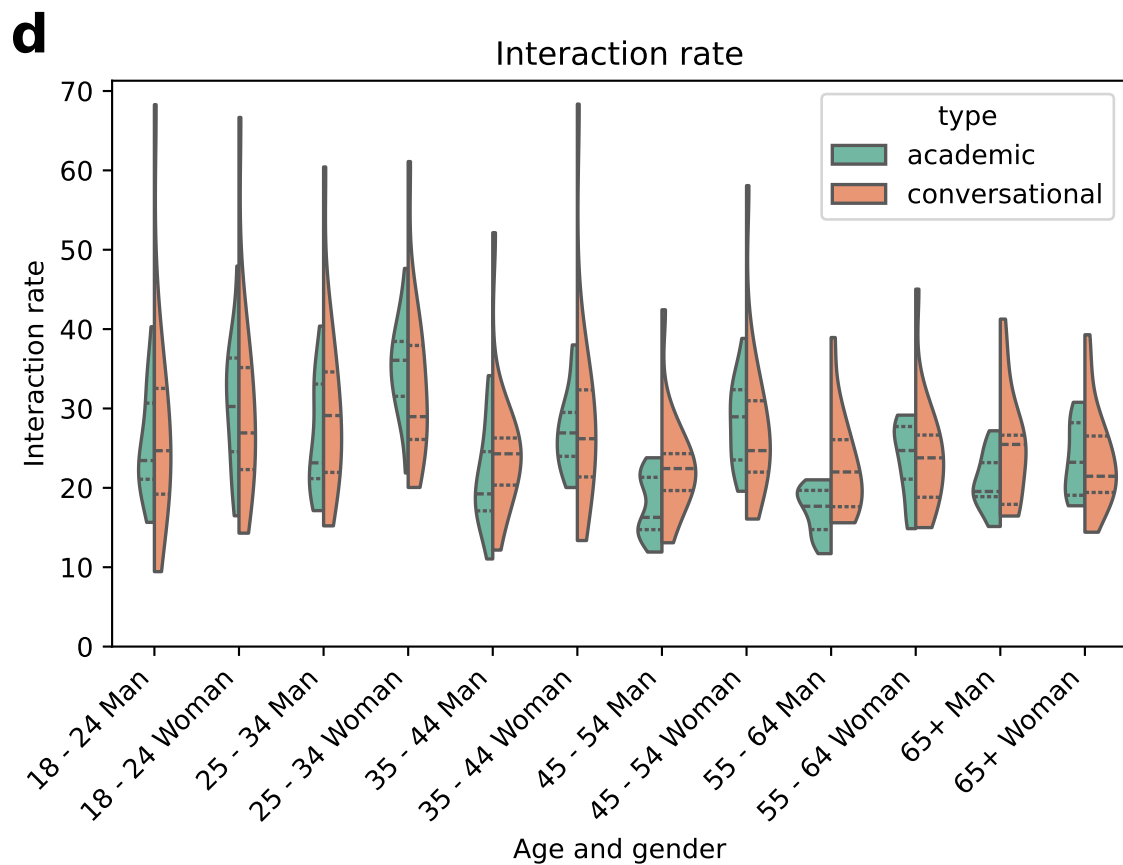

Supplement: S14 Fig — (PDF) [file pone.0352691.s015.pdf]

**a**

Interaction rate

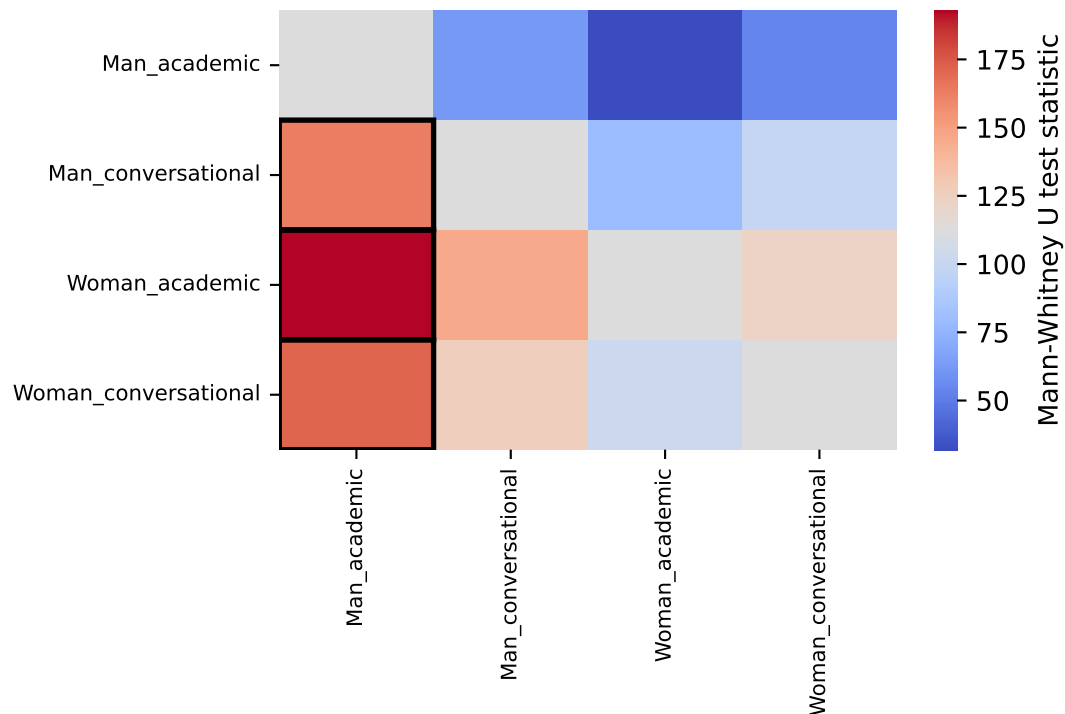**b**

Interaction rate

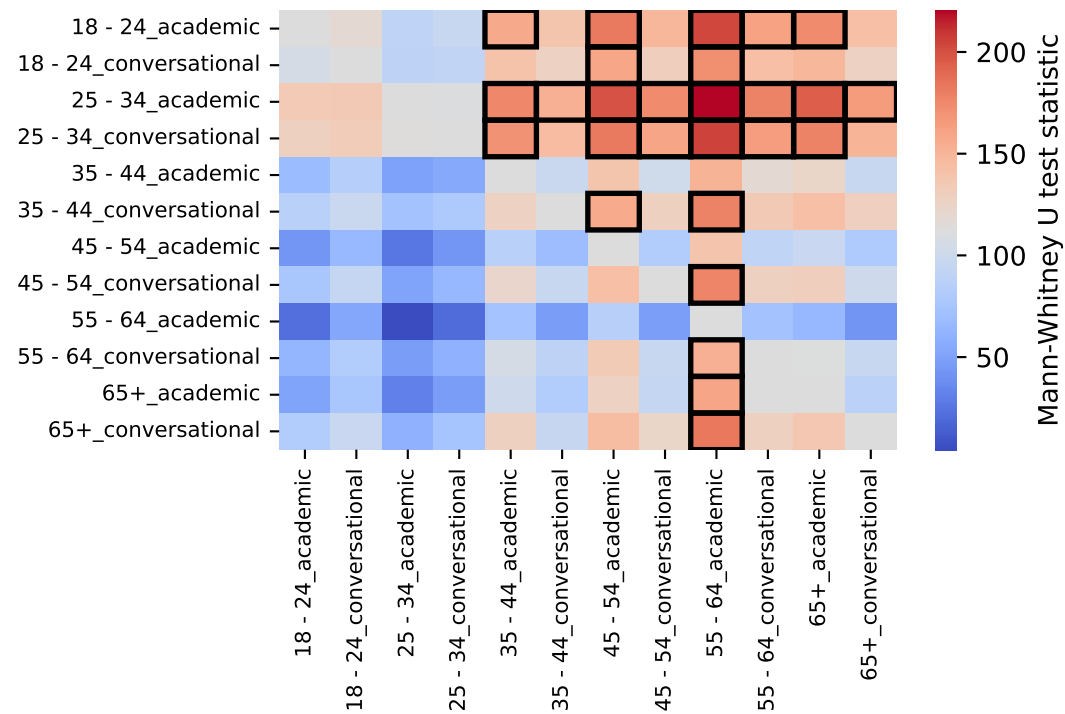**c**

Interaction rate

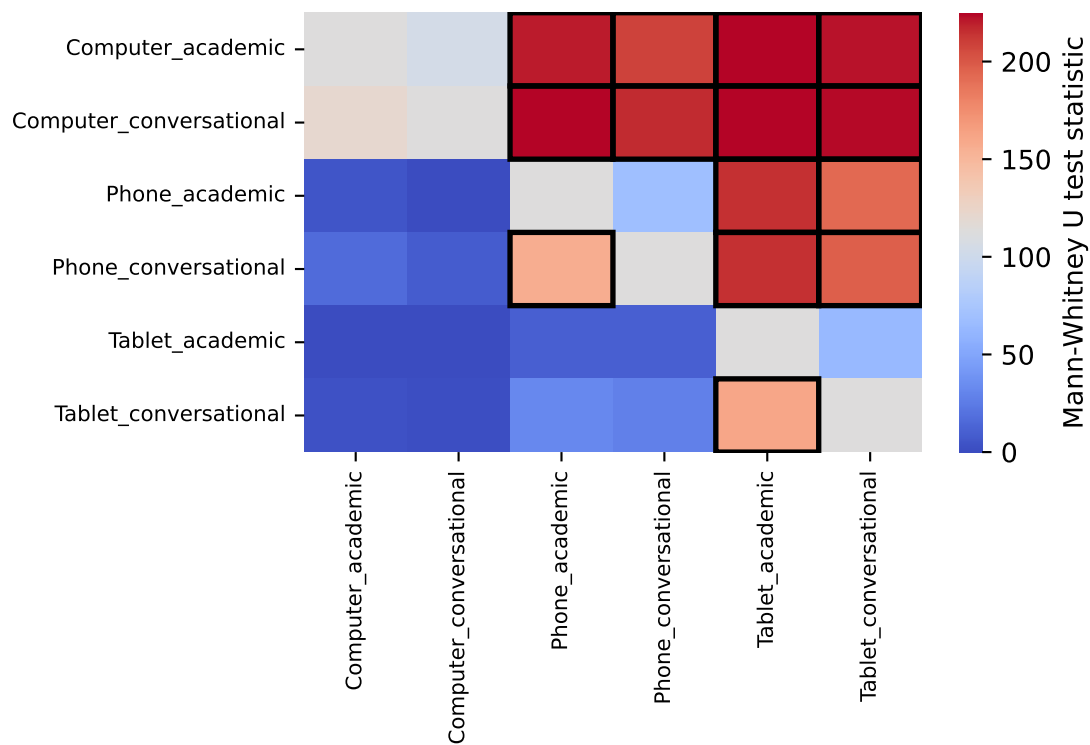**d**

Interaction rate

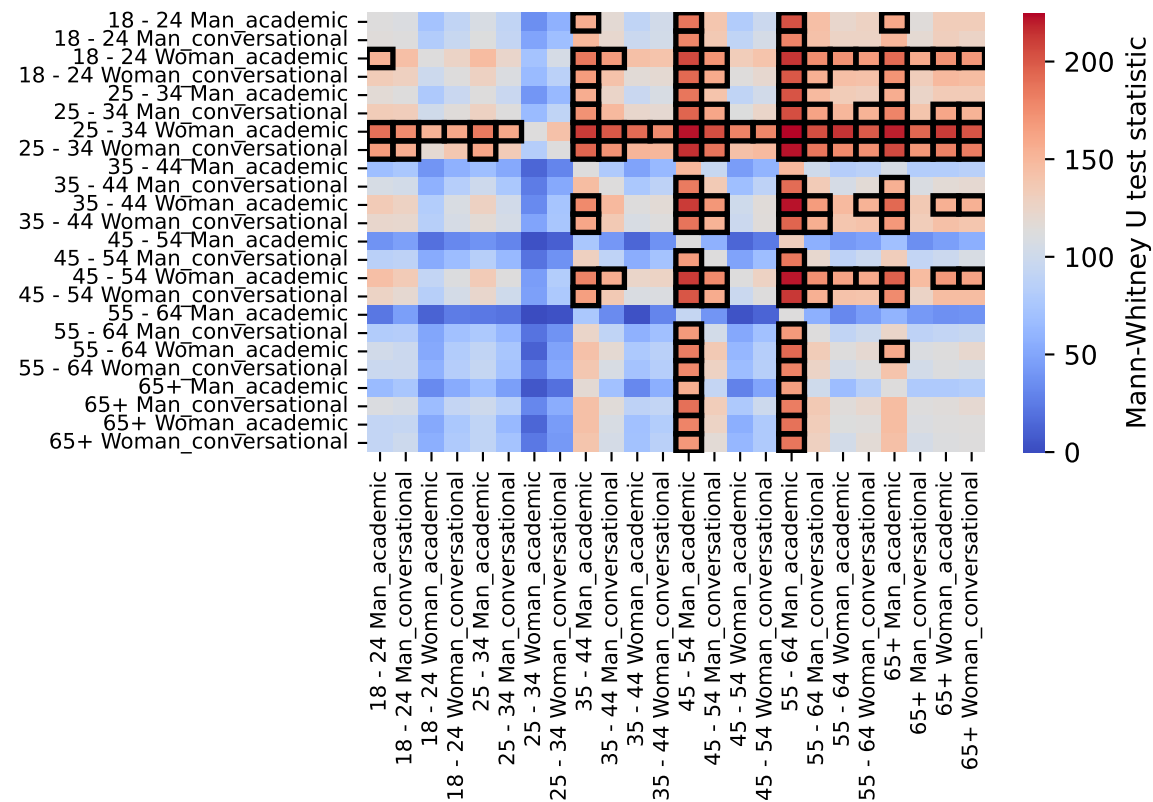

Supplement: S15 Fig — (PDF) [file pone.0352691.s016.pdf]

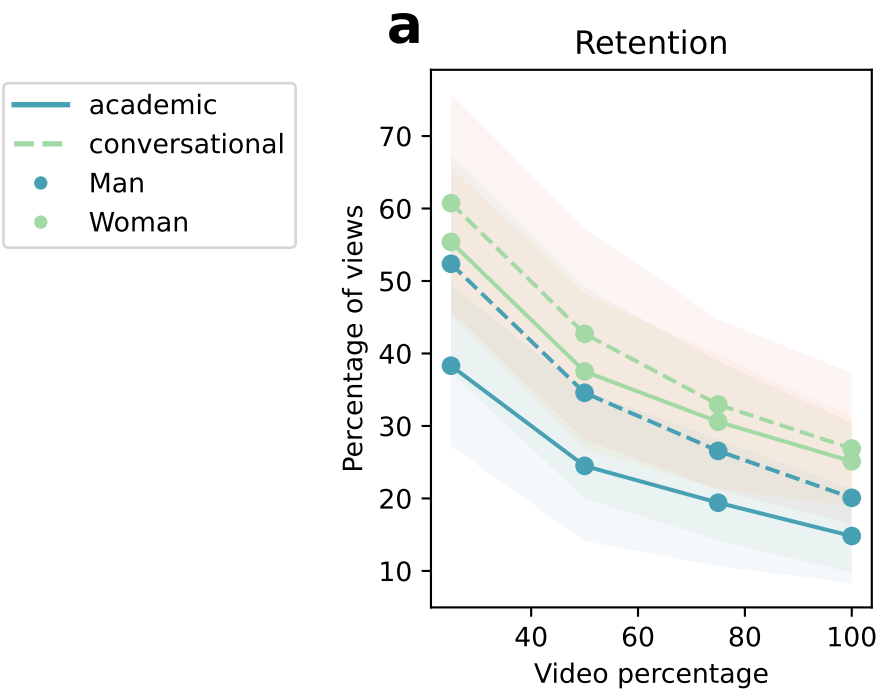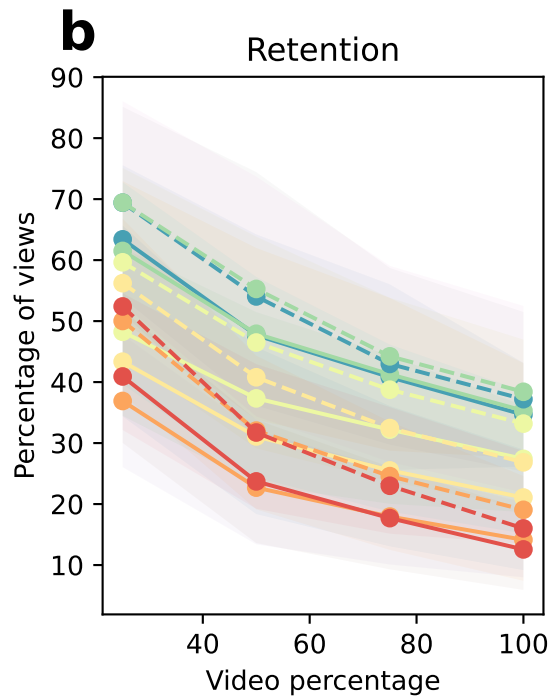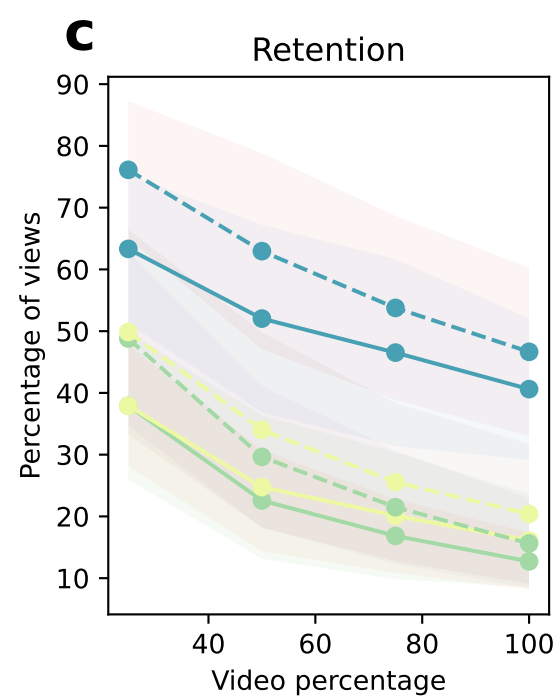

Supplement: S16 Fig — (PDF) [file pone.0352691.s017.pdf]
