# Supplementary material for: An analytical and experimental study of the energy transition discourse on YouTube
Source: PLoS One. 2026 Jul 15;21(7):e0352691. doi: 10.1371/journal.pone.0352691 (PMC13372142; doi:10.1371/journal.pone.0352691)
Supplement: S13 Fig — (PDF) [file pone.0352691.s014.pdf]

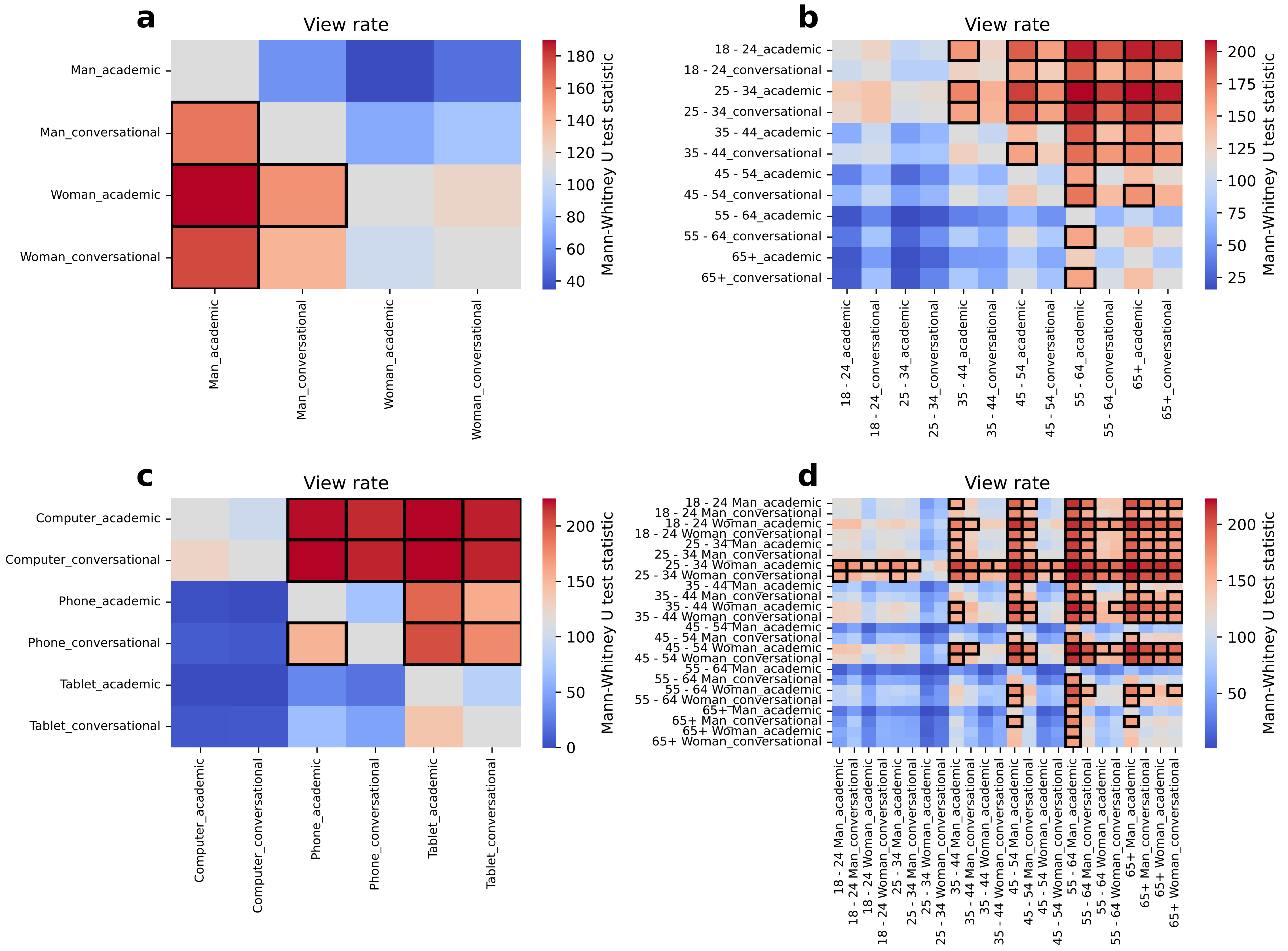

a
View rate
Man_academic
Man_conversational
Woman_academic
Woman_conversational
Mann-Whitney U test statistic
40
60
80
100
120
140
160
180
b
View rate
18 - 24_academic
18 - 24_conversational
25 - 34_academic
25 - 34_conversational
35 - 44_academic
35 - 44_conversational
45 - 54_academic
45 - 54_conversational
55 - 64_academic
55 - 64_conversational
65+_academic
65+_conversational
Mann-Whitney U test statistic
25
50
75
100
125
150
175
200
c
View rate
Computer_academic
Computer_conversational
Phone_academic
Phone_conversational
Tablet_academic
Tablet_conversational
Mann-Whitney U test statistic
0
50
100
150
200
d
View rate
18 - 24 Man_academic
18 - 24 Man_conversational
18 - 24 Woman_academic
18 - 24 Woman_conversational
25 - 34 Man_academic
25 - 34 Man_conversational
25 - 34 Woman_academic
25 - 34 Woman_conversational
35 - 44 Man_academic
35 - 44 Man_conversational
35 - 44 Woman_academic
35 - 44 Woman_conversational
45 - 54 Man_academic
45 - 54 Man_conversational
45 - 54 Woman_academic
45 - 54 Woman_conversational
55 - 64 Man_academic
55 - 64 Man_conversational
55 - 64 Woman_academic
55 - 64 Woman_conversational
65+ Man_academic
65+ Man_conversational
65+ Woman_academic
65+ Woman_conversational
Mann-Whitney U test statistic
50
100
150
200
