# Supplementary material for: An analytical and experimental study of the energy transition discourse on YouTube
Source: PLoS One. 2026 Jul 15;21(7):e0352691. doi: 10.1371/journal.pone.0352691 (PMC13372142; doi:10.1371/journal.pone.0352691)
Supplement: Attachment — Submitted filename: response.pdf [file pone.0352691.s020.pdf]

Response to Reviewers

An analytical and experimental study of the energy transition discourse on YouTube
PLOS One
PONE-D-25-62607

We sincerely thank the reviewers for their careful reading and constructive comments. Please find below the point by point response.

Reviewer 1

Reviewer Comment:
This is an interesting study and a well-written paper. However, there are some aspects that require justifications and amendments. All comments have been embedded in the paper itself.

Response:
We thank the reviewer for their positive assessment and constructive feedback. The main changes we have implemented are as follows:

- Rephrased the second part of the abstract.
- Further clarified the study significance.
- Added a literature review section focused on the methodological aspects of the paper.
- Provided more detailed justifications regarding the data collection process and analysis implemented.

These changes strengthen the clarity of our contributions, improve methodological transparency, and better position the study within the existing literature.

Reviewer Comment:
The sections that need attention are as follows: Abstract—Kindly include more specific results for this study rather than a general statement.

Response:
We have revised the abstract to explicitly report the main empirical findings, including differences in retention patterns and audience segmentation. The updated abstract now is:

"Energy production and management face significant political, economic, and environmental challenges. The rise in information consumption through social media can, under certain con-

ditions, reduce the visibility and uptake of reliable knowledge, particularly in environments characterised by high information overload, algorithmic amplification, and low media literacy. This study examines the ideas discussed in the energy transition content on YouTube, assesses the most effective methods of communicating knowledge and information, and identifies the most engaged audiences. We examine videos related to the subject, analysing the themes discussed, the language used, and the emotions conveyed on YouTube, linking language formality to user engagement. To test the relationship experimentally, original content was uploaded to YouTube through two mirror channels containing the same material but using different levels of language formality. We conducted a systematic statistical analysis of engagement data collected from YouTube and the Google Ads platform. The YouTube engagement suggests that the conversational channel reaches a broader audience, although retention across video segments varies. While user retention was higher in the early segments for the conversational content, a higher retention in the mid to late sections of academic videos was found. Data from the Google Ads platform provided a deeper understanding of engagement across user profiles. Younger individuals and women show greater engagement regardless of language style, although women demonstrate relatively greater interest in the academic content."

Reviewer Comment:
The introduction section should provide an extensive explanation of the study's significance.

Response:
We agree with the reviewer that the significance of our study should be more highlighted in the introduction. We have updated the last paragraph of the introduction with a dedicated discussion of our work significance, which now reads:

"Despite extensive research on content features and engagement, particularly in the context of scientific communication on social media [37, 41], the role of linguistic formality remains insufficiently understood, with existing findings being limited and inconclusive [13, 23].

This study contributes by combining large-scale observational analysis with a controlled experiment with two objectives: to understand how information on the energy transition is conveyed on YouTube and whether language features, specifically formality, influence engagement. As social media platforms become more influential in disseminating information on public policy and science to the general public, understanding the role of language use in engagement has become increasingly important [4, 21]. While most studies focus on observational data [8], there is a limited implementation of ad hoc experiments on online platforms. Such experiments provide a different perspective on the impact of language, communication, and platform design in engagement.

To address this gap, we analyse energy transition content on YouTube by examining its thematic structure and linguistic features, focusing on emotional tone and formality, and conduct an experiment to assess the influence of language on audience engagement and profiles. In the first part of this study, we collected videos related to energy transition keywords and analysed the topics covered, the sentiments expressed, and their correlation with engagement metrics

(views, likes, and comments). In the second part, 20 original science communication videos were produced and published on two identical channels that differed in language formality. We then conducted systematic comparisons and statistical tests to evaluate the effect of language formality on content dissemination, focusing on the engagement metrics (views, likes, and comments) and user retention. Finally, we characterise audience segments based on their differential engagement patterns, providing insights into how communication style interacts with audience heterogeneity in shaping the reach and impact of energy transition content."

Reviewer Comment:
The absence of a literature review or related works section limits its contribution. To strengthen the study's scholarly grounding, the authors should incorporate a comprehensive review of previous research and explicitly highlight how the current work is positioned. The inclusion of this section might be beneficial to novice researchers who want to gain a more intellectual understanding of its technical aspects

Response:
We have carefully considered the suggestion from the reviewer. While the journal does not require a standalone literature review section, we agree with the reviewer on the importance of strengthening the paper relation with the previous literature and contextualising the results and methodology. To avoid redundancy between a separate literature review and the introduction we have added two paragraphs extending the state-of-the-art review within the introduction. The two paragraphs now included are:

"Communication represents a critical bottleneck in the energy transition. Analysing this digital discourse therefore requires the systematic characterisation of language use, emotional tone, and thematic alignment. Language analysis initially relied on manual annotation to assess opinions and themes [12, 20], which progressively evolved into the use of closed-lexicon approaches based on dictionaries created by domain experts to identify specific word categories [9, 27]. These developments gave rise to a wide range of methods addressing distinct dimensions of language analysis, including sentiment evaluation [16], automated text categorisation [34], and the detection of stylistic formality [15]. However, these methods face limitations in scalability and in capturing contextual and semantic nuances, which were addressed through the increase of computing power and the development of probabilistic and machine learning methods [6, 14]. This transition led to significant advances in text indexing through co-occurrence patterns [11] and more robust topic analysis based on word distributions [5, 33].

More recently, transformer-based architectures, such as BERT, have enabled improved contextualised representations of textual meaning by capturing relationships between words beyond simple word frequencies [7, 28, 31]. Nowadays, topic analysis is essential to unveil thematic discussions in social media related to political events [17, 42], advertising strategies [39], public health [35, 40], and scientific topics such as climate change [2]. Sentiment analysis reveals crucial insights related to public perceptions and opinions on political and social subjects [29, 36], as well as global events and crises [3, 22, 24]. Furthermore, the analysis of language formality

allows for the comparison of formality across scientific publications and online platforms such as Reddit, Twitter, and Wikipedia [25, 26]. Together, these approaches enable large-scale examination of online discourse and highlight the need to understand how linguistic features shape information dissemination, informing the present study, which investigates energy transition content on YouTube in terms of topics, sentiment, and language formality."

We have also carefully updated the methodology section to align with the new content of the introduction.

Reviewer Comment:
Missing conclusion section.

Response:
Indeed the conclusion section is missing as we added a vast discussion section. However, we agree with the reviewer that a conclusion section would strengthen the message of the paper. Thus we have added a new conclusion section at the end of the paper that now reads:

"This study provides a combined observational and experimental assessment of how linguistic features relate to engagement with energy transition content on YouTube. By integrating large-scale content analysis with a controlled communication experiment, we examine the role of sentiment, thematic framing, and language formality in shaping audience interaction.

Our findings indicate that language style is a relevant dimension in the dissemination of energy-related content, with more conversational formats tending to be associated with higher levels of user engagement, particularly in terms of interaction rates. At the same time, engagement patterns vary across audience segments, suggesting that communication strategies may not have uniform effects across different demographic groups.

More broadly, these results highlight the importance of aligning scientific communication practices with the dynamics of digital platforms, where accessibility, tone, and framing can influence how information is received and interacted with. However, the relationship between communication style and engagement is mediated by platform-specific factors, including recommendation systems, visibility mechanisms, and audience composition, which should be considered when interpreting these findings.

Overall, this work contributes to a growing body of research on digital communication of complex societal challenges, offering empirical insights into how energy transition narratives circulate and engage audiences in online environments. Further research is needed to explore these dynamics across platforms, languages, and temporal scales, and to better understand how communication strategies can support informed and inclusive public engagement with energy transition processes. "

Reviewer Comment:
Strength of the study This study has performed a comprehensive data extraction and analysis, utilizing all relevant data modeling and formulation techniques. The discussion section provides

an extensive explanation of the results.

Response:
We appreciate the comment from the reviewer, which reinforces the robustness of our work.

Reviewer Comment:
The result is too general in the abstract

Response:
As detailed above, the abstract has been rewritten to provide a more concrete description of our results.

Reviewer Comment:
It is much beneficial to the reader if author could summarize the methods in an informative diagram to show the flow of research conducted in this study.

Response:
We agree with the reviewer that a figure summarising the approach performed by this work would be beneficial. We have added it as a new Figure 1.

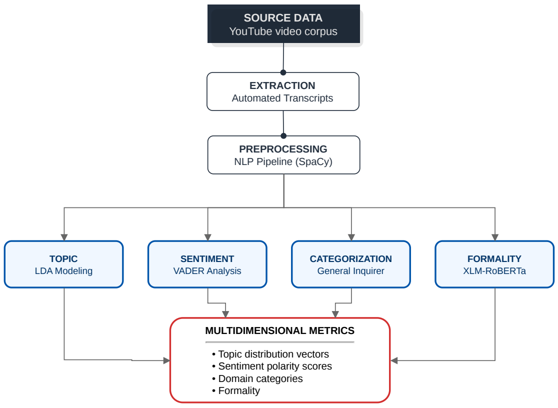

Figure 1: **Computational workflow for YouTube transcript analysis.** This pipeline details the transition from a raw video corpus to structured data. It concludes with the generation of multidimensional metrics, including topic distributions, polarity scores, formality scores and domain categories.

Reviewer Comment:
Some analyses require justification, as commented on in the paper.

Response:
We provide below a point-to-point response for the comments in the paper regarding the justification of analyses. We have provided a justification of the Spanish focus of the study, the

triadic motif analysis and the analysis of the audience distribution

Reviewer Comment:
What is the rationale for limiting the study to Spanish videos?

Response:
We thank the reviewer for this comment. We would like to clarify that the focus on Spanish videos is a deliberate design choice rather than a limitation. The study aims to analyse discourse within a specific geographical and socio-linguistic context, where language plays a central role in shaping communication styles, audience engagement, and the interpretation of energy-related narratives. In this sense, Spanish is not treated as a constrained subset, but as the appropriate linguistic domain for the research question under investigation.

Importantly, the experimental setup was specifically designed and implemented for Spanish audiences, including the creation and deployment of the video content and associated communication strategies.

From a methodological perspective, restricting the analysis to a single language ensures consistency in the application of NLP techniques, particularly for tasks such as sentiment analysis, thematic alignment, and formality detection, which are sensitive to linguistic and cultural variation.

We have clarified this rationale in the revised manuscript: *"Data extraction was restricted to Spanish to ensure contextual consistency in the observational analysis and to maximise the availability of comparable content on the platform."*

Reviewer Comment:
What is the purpose to analyze the audience distribution? Provide justifications.

Response:
The main objective is to assess if interest is homogeneous across groups or there are some significant differences (as found in the manuscript). This is crucial to identify systematic communication gaps and to assess whether engagement patterns are driven by content characteristics or by audience composition. Furthermore, it could happen that one group is overrepresented in the sample producing a bias in the general results. We have clarified this matter at the beginning of the audience analysis section by adding: *"The YouTube metrics report views and retention, yet no sociodemographic information is provided. This disaggregation is essential for gaining a deeper understanding of engagement heterogeneities across the population, allowing for the interpretation of differences between channels and avoiding the overrepresentation of certain segments."*

Reviewer Comment:
Why this study only access using these two relations - keyword pairs and keyword triad?

Response:
We considered that motifs of three keywords were sufficiently representative given the small number of nodes in the network (13) while facilitating the interpretability of the results. Moreover, the network layout provides additional information beyond triadic topic interactions. We have clarified this matter in the topic interaction section by adding: *"We have focused on dyads and triads to facilitate interpretability taking into account that they are already representative given the small network size."*

Reviewer 2

Reviewer Comment:
This is a timely and relevant study that addresses the critical intersection of science communication, social media, and the pressing global issue of the energy transition. The two-part design, combining large-scale observational analysis with a novel field experiment, is a significant strength, lending ecological validity and causal insight that purely observational studies cannot provide. The experimental creation of "mirror" channels is a particularly elegant and powerful methodological choice. The manuscript is well-structured, and the writing is generally clear. However, several methodological limitations, missing details, and minor errors need to be addressed to strengthen the paper's conclusions and ensure its reproducibility.

Response:
We thank the reviewer for highlighting the strengths of our study. Below we detail the key changes made to the manuscript:

- We added a limitation section that discusses the caveats in relation to data collection and experimental design.
- We added a new paragraph in the introduction as suggested by the reviewer that further contextualises the importance of analysing social media in the context of the energy sector.

Reviewer Comment:
The method for constructing the observational dataset is a critical limitation. The authors used the YouTube Data API to search for 13 predefined "concepts" (e.g., "renewable energy," "energy geopolitics") and limited the search to Spanish. While practical, this approach fundamentally biases the dataset towards content that explicitly uses these specific, top-level terms. It may miss nuanced or emerging discussions that use different vernacular, and it inherently excludes the broader, less formal ecosystem of content creators (like influencers or hobbyists) who might discuss energy topics without using such "official" terminology. The dataset, therefore, likely over-represents formal, institutional, or educational content. This bias should be explicitly discussed as a limitation, as it may explain the high formality scores observed in the first part of the study.

Response:
We understand the concern of the reviewer and we agree that the keywords selected could certainly bias the data collection, which is oriented to mainstream communities. While the extent of this bias cannot be fully quantified, we acknowledge this limitation. We acknowledge that the limitation is present and, thus, we have revised the limitations section accordingly:

"This study presents several limitations that should be considered. First, the observational datasets were constructed using the YouTube API based on 13 predefined energy-related keywords. This approach defines the scope of data collection to mainstream energy transition topics, which may lead to an overrepresentation of such content and could partly explain the formality scores observed. At the same time, this strategy may underrepresent niche communities that discuss energy-related issues using different levels of formality."

Reviewer Comment:
The authors correctly note that the API's video retrieval algorithm is not public. However, the potential impact of this "black box" on the dataset's composition (e.g., prioritization of popular channels, suppression of certain content) is underexplored. This uncertainty should be more prominently featured as a caveat.

Response:
The reviewer correctly identified a limitation of our study, as it was mentioned in the text. We have further highlighted this limitation by adding the following text in the limitations section:

"In addition, the video retrieval algorithm of the YouTube API is opaque and not publicly disclosed, which can generate uncertainty over the dataset composition, as we are unaware of hidden prioritisation or suppression mechanisms. Thus, we cannot ascertain how content attributes (e.g., video length, popularity, etc.) influence the type of content retrieved."

Reviewer Comment:
A significant confounder in the experiment is the use of different human narrators for the two channels. The academic channel is narrated by "academics," and the conversational channel by a narrator with "well-developed interpretative and communication abilities." The study attributes all differences in engagement to "language formality." However, perceived engagement is a function of many paralinguistic features: vocal tone, pacing, enthusiasm, perceived charisma, and even the accent or vocal quality of the narrator. It is entirely possible that the conversational channel's narrator was simply a more engaging speaker, which could drive the higher initial retention and view rates, independent of the script's formality. The authors should either reframe their conclusion to be about the combined effect of a "formal/academic presentation style" vs. an "informal/professional presentation style" or discuss this confounding factor in detail as a key limitation.

Response:
We agreee with the reviewer that the experiment design could capture paralinguistic relations.

We understand the concerns and we have added an additional paragraph in the limitations section discussing possible confounders in the experimental design:

"Regarding the experimental design, it is important to note that different narrators were used for each channel, which may introduce additional confounding factors. While the academic channel was narrated by academics, the conversational channel featured a narrator with interpretative and communication skills. Paralinguistic features may also influence engagement; therefore, the observed differences between channels may reflect not only variations in language but also differences in communicative style. These limitations highlight the need for future experimental designs in which a broader range of factors is analysed independently to gain deeper insights into the relationship between communication style and engagement."

Reviewer Comment:
The authors are advised to include the following paragraph in the introduction section: The energy transition is not solely a technological challenge but is deeply intertwined with human behavior, decision-making, and public discourse. As Seran et al. (2025)[https://doi.org/10.1016/j.eesus.2025.1000[illegible] highlight, strategic frameworks for prioritizing renewable energy are essential for achieving sustainable development goals, yet their success ultimately depends on public engagement and acceptance. This engagement is shaped by complex human factors, including how individuals process information and make decisions within energy markets, a process that Xia et al. (2026)[https://doi.org/10.1016/j.rser.2025.116310] characterize as "boundedly rational" and influenced by communication frames. For instance, the adoption of related technologies, such as new energy vehicles, is significantly affected by perceived behavioral control and social influence, extending "beyond technology" to encompass human-centric factors (Ji et al., 2026)[https://doi.org/10.1016/j.jretconser.2025.104552]. In the digital age, social media platforms like YouTube have become critical arenas where these perceptions are formed and contested. Analyzing this online discourse requires advanced computational methods, similar to those used by Yin et al. (2026) [https://doi.org/10.1016/j.ipm.2025.104413] to detect ideological positions in social media text, underscoring the value of NLP techniques for understanding public sentiment on complex policy issues like the energy transition.

Response:
We appreciate the suggestion of the reviewer as the suggested text and references further contextualise the work. We have reformulated the text to adjust the flow and it is now the second paragraph of the introduction that now reads:

"Furthermore, the energy transition is deeply intertwined with human behaviour, decision-making, and public discourse [19, 32]. Strategic frameworks for prioritising renewable energy are essential for achieving sustainable development goals, yet their success ultimately depends on public engagement and acceptance [30]. This engagement is shaped by complex human factors, including how individuals process information and make decisions within energy markets, a process influenced by cognitive mechanism and communication frames [38]. For instance, the adoption of related technologies, such as new energy vehicles, is significantly affected by perceived

behavioural control and social influence [18]. In the digital age, social media platforms, particularly YouTube as a major source of long-form informational content, have become critical arenas where these perceptions are formed and contested, highlighting the need for effective and reliable scientific communication. However, the increasing consumption of information driven by social media has made it more challenging to gain visibility and actively engage citizens [1,10]. In this context, researchers have explored how content features shape user interest and engagement."

References

[1] Scott L Althaus and David Tewksbury. Patterns of internet and traditional news media use in a networked community. *Political communication*, 17(1):21–45, 2000.

[2] Aleix Bassolas, Joan Massachs, Emanuele Cozzo, and Julian Vicens. Multifaceted polarization and information reliability in climate change discussions on social media platforms. *Royal Society Open Science*, 12(11), 2025.

[3] Muzafar Bhat, Monisa Qadri, Majid Kundroo, Naffi Ahanger, Basant Agarwal, et al. Sentiment analysis of social media response on the covid19 outbreak. *Brain, behavior, and immunity*, 87:136, 2020.

[4] Muhammad Kunta Biddinika, Mochamad Syamsiro, Srikandi Novianti, Bakhtiyor Nakhshiniev, Muhammad Aziz, and Fumitake Takahashi. Dissemination of technology information through youtube: a case of renewable energy technology. *TELKOMNIKA (Telecommunication Computing Electronics and Control)*, 17(3):1526–1538, 2019.

[5] David M Blei, Andrew Y Ng, and Michael I Jordan. Latent dirichlet allocation. *Journal of machine Learning research*, 3(Jan):993–1022, 2003.

[6] Cagatay Catal and Mehmet Nangir. A sentiment classification model based on multiple classifiers. *Applied Soft Computing*, 50:135–141, 2017.

[7] Rosario Catelli, Serena Pelosi, and Massimo Esposito. Lexicon-based vs. bert-based sentiment analysis: A comparative study in italian. *Electronics*, 11(3):374, 2022.

[8] Ilana Dubovi and Iris Tabak. Interactions between emotional and cognitive engagement with science on youtube. *Public understanding of science*, 30(6):759–776, 2021.

[9] Johannes C Eichstaedt, Margaret L Kern, David B Yaden, H Andrew Schwartz, Salvatore Giorgi, Gregory Park, Courtney A Hagan, Victoria A Tobolsky, Laura K Smith, Anneke Buffone, et al. Closed-and open-vocabulary approaches to text analysis: A review, quantitative comparison, and recommendations. *Psychological Methods*, 26(4):398, 2021.

[10] Richard Fletcher and Sora Park. The impact of trust in the news media on online news consumption and participation. *Digital journalism*, 5(10):1281–1299, 2017.

[11] Peter W Foltz. Using latent semantic indexing for information filtering. *ACM sigois bulletin*, 11(2-3):40–47, 1990.

[12] Alexander L George. *Propaganda analysis: A study of inferences made from Nazi propaganda in World War II.* The University of Chicago, 1959.

[13] Anaïs Gretry, Csilla Horváth, Nina Belei, and Allard CR van Riel. "don't pretend to be my friend!" when an informal brand communication style backfires on social media. *Journal of Business Research*, 74:77–89, 2017.

[14] Thomas L Griffiths, Mark Steyvers, and Joshua B Tenenbaum. Topics in semantic representation. *Psychological review*, 114(2):211, 2007.

[15] Francis Heylighen and Jean-Marc Dewaele. Formality of language: definition, measurement and behavioral determinants. *Interner Bericht, Center "Leo Apostel", Vrije Universiteit Brüssel*, 4(1):1–38, 1999.

[16] Clayton Hutto and Eric Gilbert. Vader: A parsimonious rule-based model for sentiment analysis of social media text. In *Proceedings of the international AAAI conference on web and social media*, volume 8, pages 216–225, 2014.

[17] Sardar Haider Waseem Ilyas, Zainab Tariq Soomro, Ahmed Anwar, Hamza Shahzad, and Ussama Yaqub. Analyzing brexit's impact using sentiment analysis and topic modeling on twitter discussion. In *Proceedings of the 21st Annual International Conference on Digital Government Research*, pages 1–6, 2020.

[18] Ting Ji, Shaofeng Wang, Jianyuan Wang, and Jie Zhang. Beyond technology: Role of technology-organization-environment-human factors in autonomous driving adoption for new energy vehicles. *Journal of Retailing and Consumer Services*, 88:104552, 2026.

[19] Nadejda Komendantova and Sonata Neumueller. Discourses about energy transition in austrian climate and energy model regions: Turning awareness into action. *Energy & Environment*, 31(8):1473–1497, 2020.

[20] Klaus Krippendorff. *Content analysis: An introduction to its methodology.* Sage publications, 2018.

[21] Feiqin Li and Asad Ullah. Social media discourse as a window into energy transition: Analyzing public perception of electric vehicles on youtube. *Energy Research & Social Science*, 129:104402, 2025.

[22] Siru Liu and Jialin Liu. Public attitudes toward covid-19 vaccines on english-language twitter: A sentiment analysis. *Vaccine*, 39(39):5499–5505, 2021.

[23] Ana Cristina Munaro, Renato Hübner Barcelos, Eliane Cristine Francisco Maffezzolli, João Pedro Santos Rodrigues, and Emerson Cabrera Paraiso. Does your style engage? linguistic styles of influencers and digital consumer engagement on youtube. *Computers in Human Behavior*, 156:108217, 2024.

[24] Meghan Nagpal, Niloofar Jalali, Diana Sherifali, Plinio Morita, and Joseph A Cafazzo. Investigating reddit data on type 2 diabetes management during the covid-19 pandemic using latent dirichlet allocation topic modeling and valence aware dictionary for sentiment reasoning analysis: Content analysis. *JMIR Formative Research*, 9:e51154, 2025.

[25] Thin Nguyen, Svetha Venkatesh, and Dinh Phung. Large-scale stylistic analysis of formality in academia and social media. In *International Conference on Web Information Systems Engineering*, pages 137–145. Springer, 2016.

[26] Thin Nguyen, Svetha Venkatesh, and Dinh Phung. Academia versus social media: A psycho-linguistic analysis. *Journal of Computational Science*, 25:228–237, 2018.

[27] James W Pennebaker, Martha E Francis, Roger J Booth, et al. Linguistic inquiry and word count: Liwc 2001. *Mahway: Lawrence Erlbaum Associates*, 71(2001):2001, 2001.

[28] Arik Reuter, Anton Thielmann, Christoph Weisser, Benjamin Säfken, and Thomas Kneib. Probabilistic topic modeling with transformer representations. *IEEE Transactions on Neural Networks and Learning Systems*, 2025.

[29] Rodrigo Sandoval-Almazan and David Valle-Cruz. Facebook impact and sentiment analysis on political campaigns. In *Proceedings of the 19th annual international conference on digital government research: governance in the data age*, pages 1–7, 2018.

[30] Karmila Seran, James Rotimi, and An Le. Decision making support tool for renewable energy prioritization to achieve sustainable development goals (sdgs): Conceptual framework. *Energy & Environmental Sustainability*, 1(4):100044, 2025.

[31] Mrityunjay Singh, Amit Kumar Jakhar, and Shivam Pandey. Sentiment analysis on the impact of coronavirus in social life using the bert model. *Social Network Analysis and Mining*, 11(1):33, 2021.

[32] Linda Steg, Goda Perlaviciute, and Ellen Van der Werff. Understanding the human dimensions of a sustainable energy transition. *Frontiers in psychology*, 6:805, 2015.

[33] Mark Steyvers and Tom Griffiths. Probabilistic topic models. In *Handbook of latent semantic analysis*, pages 439–460. Psychology Press, 2007.

[34] Philip J. Stone, Dexter C. Dunphy, Marshall S. Smith, and Daniel M. Ogilvie. *The General Inquirer: A Computer Approach to Content Analysis*. MIT Press, Cambridge, MA, 1966.

[35] Mike Donald Tapi Nzali, Sandra Bringay, Christian Lavergne, Caroline Mollevi, and Thomas Opitz. What patients can tell us: topic analysis for social media on breast cancer. *JMIR medical informatics*, 5(3):e23, 2017.

[36] Hao Wang, Doğan Can, Abe Kazemzadeh, François Bar, and Shrikanth Narayanan. A system for real-time twitter sentiment analysis of 2012 us presidential election cycle. In *Proceedings of the ACL 2012 system demonstrations*, pages 115–120, 2012.

[37] Dustin J Welbourne and Will J Grant. Science communication on youtube: Factors that affect channel and video popularity. *Public understanding of science*, 25(6):706–718, 2016.

[38] Yuanxing Xia, Ke Wang, Yu Huang, Tinjun Lin, Linjun Shi, and Feng Wu. Bounded rational decision-making modeling and analysis in local energy markets: A state-of-the-art review. *Renewable and Sustainable Energy Reviews*, 226:116310, 2026.

[39] Sifan Xu and Ying Xiong. Setting socially mediated engagement parameters: A topic modeling and text analytic approach to examining polarized discourses on gillette's campaign. *Public Relations Review*, 46(5):101959, 2020.

[40] Jia Xue, Junxiang Chen, Chen Chen, Chengda Zheng, Sijia Li, and Tingshao Zhu. Public discourse and sentiment during the covid 19 pandemic: Using latent dirichlet allocation for topic modeling on twitter. *PloS one*, 15(9):e0239441, 2020.

[41] Shiyu Yang, Dominique Brossard, Dietram A Scheufele, and Michael A Xenos. The science of youtube: What factors influence user engagement with online science videos? *Plos one*, 17(5):e0267697, 2022.

[42] Maggie Mengqing Zhang, Xiao Wang, and Yang Hu. Strategic framing matters but varies: A structural topic modeling approach to analyzing china's foreign propaganda about the 2019 hong kong protests on twitter. *Social Science Computer Review*, 41(1):265–285, 2023.
