# Supplementary material for: An analytical and experimental study of the energy transition discourse on YouTube
Source: PLoS One. 2026 Jul 15;21(7):e0352691. doi: 10.1371/journal.pone.0352691 (PMC13372142; doi:10.1371/journal.pone.0352691)
Supplement: Peer review history

# Peer review: Decision Letter 0

**DOI:** 10.1371/journal.pone.0352691.r001

**Authors:** Cheong Kim

4 Mar 2026

PONE-D-25-62607

An analytical and experimental study of the energy transition discourse on YouTube

PLOS One

Dear Dr. Bassolas,

Thank you for submitting your manuscript to PLOS ONE. After careful consideration, we feel that it has merit but does not fully meet PLOS ONE’s publication criteria as it currently stands. Therefore, we invite you to submit a revised version of the manuscript that addresses the points raised during the review process.

Please submit your revised manuscript by Apr 18 2026 11:59PM. If you will need more time than this to complete your revisions, please reply to this message or contact the journal office at plosone@plos.org. When you're ready to submit your revision, log on to https://www.editorialmanager.com/pone/ and select the 'Submissions Needing Revision' folder to locate your manuscript file.

Please include the following items when submitting your revised manuscript:

- A letter that responds to each point raised by the academic editor and reviewer(s). You should upload this letter as a separate file labeled 'Response to Reviewers'.
- A marked-up copy of your manuscript that highlights changes made to the original version. You should upload this as a separate file labeled 'Revised Manuscript with Track Changes'.
- An unmarked version of your revised paper without tracked changes. You should upload this as a separate file labeled 'Manuscript'.

If you would like to make changes to your financial disclosure, please include your updated statement in your cover letter. Guidelines for resubmitting your figure files are available below the reviewer comments at the end of this letter.

If applicable, we recommend that you deposit your laboratory protocols in protocols.io to enhance the reproducibility of your results. Protocols.io assigns your protocol its own identifier (DOI) so that it can be cited independently in the future. For instructions see: https://journals.plos.org/plosone/s/submission-guidelines#loc-laboratory-protocols. Additionally, PLOS ONE offers an option for publishing peer-reviewed Lab Protocol articles, which describe protocols hosted on protocols.io. Read more information on sharing protocols at https://plos.org/protocols?utm_medium=editorial-email&utm_source=authorletters&utm_campaign=protocols.

We look forward to receiving your revised manuscript.

Kind regards,

Cheong Kim

Academic Editor

PLOS One

**Journal Requirements:**

1. When submitting your revision, we need you to address these additional requirements.

Please ensure that your manuscript meets PLOS ONE's style requirements, including those for file naming. The PLOS ONE style templates can be found at

https://journals.plos.org/plosone/s/file?id=wjVg/PLOSOne_formatting_sample_main_body.pdf and

https://journals.plos.org/plosone/s/file?id=ba62/PLOSOne_formatting_sample_title_authors_affiliations.pdf

2. Please note that PLOS One has specific guidelines on code sharing for submissions in which author-generated code underpins the findings in the manuscript. In these cases, we expect all author-generated code to be made available without restrictions upon publication of the work. Please review our guidelines at https://journals.plos.org/plosone/s/materials-and-software-sharing#loc-sharing-code and ensure that your code is shared in a way that follows best practice and facilitates reproducibility and reuse.

3. We note that the grant information you provided in the ‘Funding Information’ and ‘Financial Disclosure’ sections do not match.

When you resubmit, please ensure that you provide the correct grant numbers for the awards you received for your study in the ‘Funding Information’ section.

4. Thank you for stating in your Funding Statement:

This work was supported by project 23S06035-001, funded by the 2023 Research and Innovation Grant Program of the Barcelona City Council in collaboration with the “La Caixa” Foundation.

Please provide an amended statement that declares *all* the funding or sources of support (whether external or internal to your organization) received during this study, as detailed online in our guide for authors at http://journals.plos.org/plosone/s/submit-now.  Please also include the statement “There was no additional external funding received for this study.” in your updated Funding Statement.

Please include your amended Funding Statement within your cover letter. We will change the online submission form on your behalf.

5. Thank you for stating the following financial disclosure:

This work was supported by project 23S06035-001, funded by the 2023 Research and Innovation Grant Program of the Barcelona City Council in collaboration with the “La Caixa” Foundation.

Please state what role the funders took in the study.  If the funders had no role, please state: "The funders had no role in study design, data collection and analysis, decision to publish, or preparation of the manuscript."

If this statement is not correct you must amend it as needed.

Please include this amended Role of Funder statement in your cover letter; we will change the online submission form on your behalf.

6. Please note that your Data Availability Statement is currently missing the repository name and/or the DOI/accession number of each dataset and a direct link to access each database. If your manuscript is accepted for publication, you will be asked to provide these details on a very short timeline. We therefore suggest that you provide this information now, though we will not hold up the peer review process if you are unable.

7. Please upload a new copy of Figures S3, S4, S5, S6, S7, S12, S13, S14 and S16, as the detail is not clear. Please follow the link for more information:  https://journals.plos.org/plosone/s/figures

8. We note that Figure S8 in your submission contain copyrighted images. All PLOS content is published under the Creative Commons Attribution License (CC BY 4.0), which means that the manuscript, images, and Supporting Information files will be freely available online, and any third party is permitted to access, download, copy, distribute, and use these materials in any way, even commercially, with proper attribution. For more information, see our copyright guidelines: http://journals.plos.org/plosone/s/licenses-and-copyright.

We require you to either present written permission from the copyright holder to publish these figures specifically under the CC BY 4.0 license, or remove the figures from your submission:

a. You may seek permission from the original copyright holder of Figure S8 to publish the content specifically under the CC BY 4.0 license.

We recommend that you contact the original copyright holder with the Content Permission Form (http://journals.plos.org/plosone/s/file?id=7c09/content-permission-form.pdf) and the following text:

“I request permission for the open-access journal PLOS ONE to publish XXX under the Creative Commons Attribution License (CCAL) CC BY 4.0 (http://creativecommons.org/licenses/by/4.0/). Please be aware that this license allows unrestricted use and distribution, even commercially, by third parties. Please reply and provide explicit written permission to publish XXX under a CC BY license and complete the attached form.”

Please upload the completed Content Permission Form or other proof of granted permissions as an "Other" file with your submission.

In the figure caption of the copyrighted figure, please include the following text: “Reprinted from [ref] under a CC BY license, with permission from [name of publisher], original copyright [original copyright year].”

b. If you are unable to obtain permission from the original copyright holder to publish these figures under the CC BY 4.0 license or if the copyright holder’s requirements are incompatible with the CC BY 4.0 license, please either i) remove the figure or ii) supply a replacement figure that complies with the CC BY 4.0 license. Please check copyright information on all replacement figures and update the figure caption with source information. If applicable, please specify in the figure caption text when a figure is similar but not identical to the original image and is therefore for illustrative purposes only.

9. We note that Figure S8 includes an image of a participant in the study.

As per the PLOS ONE policy (http://journals.plos.org/plosone/s/submission-guidelines#loc-human-subjects-research) on papers that include identifying, or potentially identifying, information, the individual(s) or parent(s)/guardian(s) must be informed of the terms of the PLOS open-access (CC-BY) license and provide specific permission for publication of these details under the terms of this license. Please download the Consent Form for Publication in a PLOS Journal (http://journals.plos.org/plosone/s/file?id=8ce6/plos-consent-form-english.pdf). The signed consent form should not be submitted with the manuscript, but should be securely filed in the individual's case notes. Please amend the methods section and ethics statement of the manuscript to explicitly state that the patient/participant has provided consent for publication: “The individual in this manuscript has given written informed consent (as outlined in PLOS consent form) to publish these case details”.

If you are unable to obtain consent from the subject of the photograph, you will need to remove the figure and any other textual identifying information or case descriptions for this individual.

10. If the reviewer comments include a recommendation to cite specific previously published works, please review and evaluate these publications to determine whether they are relevant and should be cited. There is no requirement to cite these works unless the editor has indicated otherwise.

[Note: HTML markup is below. Please do not edit.]

Reviewers' comments:

Reviewer's Responses to Questions

**Comments to the Author**

1. Is the manuscript technically sound, and do the data support the conclusions?

The manuscript must describe a technically sound piece of scientific research with data that supports the conclusions. Experiments must have been conducted rigorously, with appropriate controls, replication, and sample sizes. The conclusions must be drawn appropriately based on the data presented.

Reviewer #1: Yes

Reviewer #2: Yes

**********

2. Has the statistical analysis been performed appropriately and rigorously?

Reviewer #1: Yes

Reviewer #2: I Don't Know

**********

3. Have the authors made all data underlying the findings in their manuscript fully available?

The PLOS Data policy requires authors to make all data underlying the findings described in their manuscript fully available without restriction, with rare exception (please refer to the Data Availability Statement in the manuscript PDF file). The data should be provided as part of the manuscript or its supporting information, or deposited to a public repository. For example, in addition to summary statistics, the data points behind means, medians and variance measures should be available. If there are restrictions on publicly sharing data—e.g. participant privacy or use of data from a third party—those must be specified.

Reviewer #1: No

Reviewer #2: Yes

**********

4. Is the manuscript presented in an intelligible fashion and written in standard English?

PLOS ONE does not copyedit accepted manuscripts, so the language in submitted articles must be clear, correct, and unambiguous. Any typographical or grammatical errors should be corrected at revision, so please note any specific errors here.

Reviewer #1: Yes

Reviewer #2: Yes

**********

5. Review Comments to the Author

Please use the space provided to explain your answers to the questions above. You may also include additional comments for the author, including concerns about dual publication, research ethics, or publication ethics. (Please upload your review as an attachment if it exceeds 20,000 characters)

**Reviewer #1:**  This is an interesting study and a well-written paper. However, there are some aspects that require justifications and amendments. All comments have been embedded in the paper itself. The sections that need attention are as follows:

1. Abstract—Kindly include more specific results for this study rather than a general statement.

2. The introduction section should provide an extensive explanation of the study's significance.

3. The absence of a literature review or related works section limits its contribution. To strengthen the study’s scholarly grounding, the authors should incorporate a comprehensive review of previous research and explicitly highlight how the current work is positioned. The inclusion of this section might be beneficial to novice researchers who want to gain a more intellectual understanding of its technical aspects.

4. Some analyses require justification, as commented on in the paper.

5. Missing conclusion section.

Strength of the study

This study has performed a comprehensive data extraction and analysis, utilizing all relevant data modeling and formulation techniques. The discussion section provides an extensive explanation of the results.

**Reviewer #2:**  This is a timely and relevant study that addresses the critical intersection of science communication, social media, and the pressing global issue of the energy transition. The two-part design, combining large-scale observational analysis with a novel field experiment, is a significant strength, lending ecological validity and causal insight that purely observational studies cannot provide. The experimental creation of "mirror" channels is a particularly elegant and powerful methodological choice. The manuscript is well-structured, and the writing is generally clear. However, several methodological limitations, missing details, and minor errors need to be addressed to strengthen the paper's conclusions and ensure its reproducibility.

The method for constructing the observational dataset is a critical limitation. The authors used the YouTube Data API to search for 13 predefined "concepts" (e.g., "renewable energy," "energy geopolitics") and limited the search to Spanish. While practical, this approach fundamentally biases the dataset towards content that explicitly uses these specific, top-level terms. It may miss nuanced or emerging discussions that use different vernacular, and it inherently excludes the broader, less formal ecosystem of content creators (like influencers or hobbyists) who might discuss energy topics without using such "official" terminology. The dataset, therefore, likely over-represents formal, institutional, or educational content. This bias should be explicitly discussed as a limitation, as it may explain the high formality scores observed in the first part of the study.

The authors correctly note that the API's video retrieval algorithm is not public. However, the potential impact of this "black box" on the dataset's composition (e.g., prioritization of popular channels, suppression of certain content) is underexplored. This uncertainty should be more prominently featured as a caveat.

A significant confounder in the experiment is the use of different human narrators for the two channels. The academic channel is narrated by "academics," and the conversational channel by a narrator with "well-developed interpretative and communication abilities." The study attributes all differences in engagement to "language formality." However, perceived engagement is a function of many paralinguistic features: vocal tone, pacing, enthusiasm, perceived charisma, and even the accent or vocal quality of the narrator. It is entirely possible that the conversational channel's narrator was simply a more engaging speaker, which could drive the higher initial retention and view rates, independent of the script's formality. The authors should either reframe their conclusion to be about the combined effect of a "formal/academic presentation style" vs. an "informal/professional presentation style" or discuss this confounding factor in detail as a key limitation.

The authors are advised to include the following paragraph in the introduction section:

The energy transition is not solely a technological challenge but is deeply intertwined with human behavior, decision-making, and public discourse. As Seran et al. (2025)[https://doi.org/10.1016/j.eesus.2025.100044] highlight, strategic frameworks for prioritizing renewable energy are essential for achieving sustainable development goals, yet their success ultimately depends on public engagement and acceptance. This engagement is shaped by complex human factors, including how individuals process information and make decisions within energy markets, a process that Xia et al. (2026)[https://doi.org/10.1016/j.rser.2025.116310] characterize as "boundedly rational" and influenced by communication frames. For instance, the adoption of related technologies, such as new energy vehicles, is significantly affected by perceived behavioral control and social influence, extending "beyond technology" to encompass human-centric factors (Ji et al., 2026)[https://doi.org/10.1016/j.jretconser.2025.104552]. In the digital age, social media platforms like YouTube have become critical arenas where these perceptions are formed and contested. Analyzing this online discourse requires advanced computational methods, similar to those used by Yin et al. (2026) [https://doi.org/10.1016/j.ipm.2025.104413] to detect ideological positions in social media text, underscoring the value of NLP techniques for understanding public sentiment on complex policy issues like the energy transition.

**********

6. PLOS authors have the option to publish the peer review history of their article (what does this mean?). If published, this will include your full peer review and any attached files.

If you choose “no”, your identity will remain anonymous but your review may still be made public.

**Do you want your identity to be public for this peer review?**  For information about this choice, including consent withdrawal, please see our Privacy Policy.

Reviewer #1: No

Reviewer #2: No

**********

[NOTE: If reviewer comments were submitted as an attachment file, they will be attached to this email and accessible via the submission site. Please log into your account, locate the manuscript record, and check for the action link "View Attachments". If this link does not appear, there are no attachment files.]

To ensure your figures meet our technical requirements, please review our figure guidelines: https://journals.plos.org/plosone/s/figures

You may also use PLOS’s free figure tool, NAAS, to help you prepare publication quality figures: https://journals.plos.org/plosone/s/figures#loc-tools-for-figure-preparation.

NAAS will assess whether your figures meet our technical requirements by comparing each figure against our figure specifications.

Attachment Submitted filename: PONE-D-25-62607commented.pdf

# Author comment: Author response to Decision Letter 1

**DOI:** 10.1371/journal.pone.0352691.r002

14 May 2026

This is an interesting study and a well-written paper. However, there are some aspects that require justifications and amendments. All comments have been embedded in the paper itself.

We thank the reviewer for their positive assessment and constructive feedback. The main changes we have implemented are as follows:

- Rephrased the second part of the abstract.

- Further clarified the study significance.

- Added a literature review section focused on the methodological aspects of the paper.

- Provided more detailed justifications regarding the data collection process and analysis implemented.

These changes strengthen the clarity of our contributions, improve methodological transparency, and better position the study within the existing literature.

The sections that need attention are as follows: Abstract—Kindly include more specific results for this study rather than a general statement.

We have revised the abstract to explicitly report the main empirical findings, including differences in retention patterns and audience segmentation. The updated abstract now is:

"Energy production and management face significant political, economic, and environmental challenges. The rise in information consumption through social media can, under certain conditions, reduce the visibility and uptake of reliable knowledge, particularly in environments characterised by high information overload, algorithmic amplification, and low media literacy. This study examines the ideas discussed in the energy transition content on YouTube, assesses the most effective methods of communicating knowledge and information, and identifies the most engaged audiences. We examine videos related to the subject, analysing the themes discussed, the language used, and the emotions conveyed on YouTube, linking language formality to user engagement. To test the relationship experimentally, original content was uploaded to YouTube through two mirror channels containing the same material but using different levels of language formality. We conducted a systematic statistical analysis of engagement data collected from YouTube and the Google Ads platform. The YouTube engagement suggests that the conversational channel reaches a broader audience, although retention across video segments varies. While user retention was higher in the early segments for the conversational content, a higher retention in the mid to late sections of academic videos was found. Data from the Google Ads platform provided a deeper understanding of engagement across user profiles. Younger individuals and women show greater engagement regardless of language style, although women demonstrate relatively greater interest in the academic content."

The introduction section should provide an extensive explanation of the study's significance.

We agree with the reviewer that the significance of our study should be more highlighted in the introduction. We have updated the last paragraph of the introduction with a dedicated discussion of our work significance, which now reads:

"Despite extensive research on content features and engagement, particularly in the context of scientific communication on social media, the role of linguistic formality remains insufficiently understood, with existing findings being limited and inconclusive.

This study contributes by combining large-scale observational analysis with a controlled experiment with two objectives: to understand how information on the energy transition is conveyed on YouTube and whether language features, specifically formality, influence engagement. As social media platforms become more influential in disseminating information on public policy and science to the general public, understanding the role of language use in engagement has become increasingly important. While most studies focus on observational data, there is a limited implementation of ad hoc experiments on online platforms. Such experiments provide a different perspective on the impact of language, communication, and platform design in engagement.

To address this gap, we analyse energy transition content on YouTube by examining its thematic structure and linguistic features, focusing on emotional tone and formality, and conduct an experiment to assess the influence of language on audience engagement and profiles. In the first part of this study, we collected videos related to energy transition keywords and analysed the topics covered, the sentiments expressed, and their correlation with engagement metrics (views, likes, and comments). In the second part, 20 original science communication videos were produced and published on two identical channels that differed in language formality. We then conducted systematic comparisons and statistical tests to evaluate the effect of language formality on content dissemination, focusing on the engagement metrics (views, likes, and comments) and user retention. Finally, we characterise audience segments based on their differential engagement patterns, providing insights into how communication style interacts with audience heterogeneity in shaping the reach and impact of energy transition content."

The absence of a literature review or related works section limits its contribution. To strengthen the study’s scholarly grounding, the authors should incorporate a comprehensive review of previous research and explicitly highlight how the current work is positioned. The inclusion of this section might be beneficial to novice researchers who want to gain a more intellectual understanding of its technical aspects

We have carefully considered the suggestion from the reviewer. While the journal does not require a standalone literature review section, we agree with the reviewer on the importance of strengthening the paper relation with the previous literature and contextualising the results and methodology. To avoid redundancy between a separate literature review and the introduction we have added two paragraphs extending the state-of-the-art review within the introduction. The two paragraphs now included are:

"Communication represents a critical bottleneck in the energy transition. Analysing this digital discourse therefore requires the systematic characterisation of language use, emotional tone, and thematic alignment. Language analysis initially relied on manual annotation to assess opinions and themes, which progressively evolved into the use of closed-lexicon approaches based on dictionaries created by domain experts to identify specific word categories. These developments gave rise to a wide range of methods addressing distinct dimensions of language analysis, including sentiment evaluation, automated text categorisation, and the detection of stylistic formality. However, these methods face limitations in scalability and in capturing contextual and semantic nuances, which were addressed through the increase of computing power and the development of probabilistic and machine learning methods. This transition led to significant advances in text indexing through co-occurrence patterns and more robust topic analysis based on word distributions.

More recently, transformer-based architectures, such as BERT, have enabled improved contextualised representations of textual meaning by capturing relationships between words beyond simple word frequencies. Nowadays, topic analysis is essential to unveil thematic discussions in social media related to political events, advertising strategies, public health, and scientific topics such as climate change. Sentiment analysis reveals crucial insights related to public perceptions and opinions on political and social subjects, as well as global events and crises. Furthermore, the analysis of language formality allows for the comparison of formality across scientific publications and online platforms such as Reddit, Twitter, and Wikipedia.

Together, these approaches enable large-scale examination of online discourse and highlight the need to understand how linguistic features shape information dissemination, informing the present study, which investigates energy transition content on YouTube in terms of topics, sentiment, and language formality."

We have also carefully updated the methodology section to align with the new content of the introduction.

Missing conclusion section.

Indeed the conclusion section is missing as we added a vast discussion section. However, we agree with the reviewer that a conclusion section would strengthen the message of the paper. Thus we have added a new conclusion section at the end of the paper that now reads:

"This study provides a combined observational and experimental assessment of how linguistic features relate to engagement with energy transition content on YouTube. By integrating large-scale content analysis with a controlled communication experiment, we examine the role of sentiment, thematic framing, and language formality in shaping audience interaction.

Our findings indicate that language style is a relevant dimension in the dissemination of energy-related content, with more conversational formats tending to be associated with higher levels of user engagement, particularly in terms of interaction rates. At the same time, engagement patterns vary across audience segments, suggesting that communication strategies may not have uniform effects across different demographic groups.

More broadly, these results highlight the importance of aligning scientific communication practices with the dynamics of digital platforms, where accessibility, tone, and framing can influence how information is received and interacted with. However, the relationship between communication style and engagement is mediated by platform-specific factors, including recommendation systems, visibility mechanisms, and audience composition, which should be considered when interpreting these findings.

Overall, this work contributes to a growing body of research on digital communication of complex societal challenges, offering empirical insights into how energy transition narratives circulate and engage audiences in online environments. Further research is needed to explore these dynamics across platforms, languages, and temporal scales, and to better understand how communication strategies can support informed and inclusive public engagement with energy transition processes. "

Strength of the study

This study has performed a comprehensive data extraction and analysis, utilizing all relevant data modeling and formulation techniques. The discussion section provides an extensive explanation of the results.

We appreciate the comment from the reviewer, which reinforces the robustness of our work.

The result is too general in the abstract

As detailed above, the abstract has been rewritten to provide a more concrete description of our results.

It is much beneficial to the reader if author could summarize the methods in an informative diagram to show the flow of research conducted in this study.

We agree with the reviewer that a figure summarising the approach performed by this work would be beneficial. We have added it as a new Figure 1.

Some analyses require justification, as commented on in the paper.

We provide below a point-to-point response for the comments in the paper regarding the justification of analyses. We have provided a justification of the Spanish focus of the study, the triadic motif analysis and the analysis of the audience distribution

What is the rationale for limiting the study to Spanish videos?

We thank the reviewer for this comment. We would like to clarify that the focus on Spanish videos is a deliberate design choice rather than a limitation. The study aims to analyse discourse within a specific geographical and socio-linguistic context, where language plays a central role in shaping communication styles, audience engagement, and the interpretation of energy-related narratives. In this sense, Spanish is not treated as a constrained subset, but as the appropriate linguistic domain for the research question under investigation.

Importantly, the experimental setup was specifically designed and implemented for Spanish audiences, including the creation and deployment of the video content and associated communication strategies.

From a methodological perspective, restricting the analysis to a single language ensures consistency in the application of NLP techniques, particularly for tasks such as sentiment analysis, thematic alignment, and formality detection, which are sensitive to linguistic and cultural variation.

We have clarified this rationale in the revised manuscript: Data extraction was restricted to Spanish to ensure contextual consistency in the observational analysis and to maximise the availability of comparable content on the platform."

What is the purpose to analyze the audience distribution? Provide justifications.

The main objective is to assess if interest is homogeneous across groups or there are some significant differences (as found in the manuscript). This is crucial to identify systematic communication gaps and to assess whether engagement patterns are driven by content characteristics or by audience composition. Furthermore, it could happen that one group is overrepresented in the sample producing a bias in the general results. We have clarified this matter at the beginning of the audience analysis section by adding: "The YouTube metrics report views and retention, yet no sociodemographic information is provided. This disaggregation is essential for gaining a deeper understanding of engagement heterogeneities across the population, allowing for the interpretation of differences between channels and avoiding the overrepresentation of certain segments."

Why this study only access using these two relations - keyword pairs and keyword triad?

We considered that motifs of three keywords were sufficiently representative given the small number of nodes in the network (13) while facilitating the interpretability of the results. Moreover, the network layout provides additional information beyond triadic topic interactions. We have clarified this matter in the topic interaction section by adding: "We have focused on dyads and triads to facilitate interpretability taking into account that they are already representative given the small network size."

Reviewer 2

This is a timely and relevant study that addresses the critical intersection of science communication, social media, and the pressing global issue of the energy transition. The two-part design, combining large-scale observational analysis with a novel field experiment, is a significant strength, lending ecological validity and causal insight that purely observational studies cannot provide. The experimental creation of "mirror" channels is a particularly elegant and powerful methodological choice. The manuscript is well-structured, and the writing is generally clear. However, several methodological limitations, missing details, and minor errors need to be addressed to strengthen the paper's conclusions and ensure its reproducibility.

We thank the reviewer for highlighting the strengths of our study. Below we detail the key changes made to the manuscript:

- We added a limitation section that discusses the caveats in relation to data collection and experimental design.

- We added a new paragraph in the introduction as suggested by the reviewer that further contextualises the importance of analysing social media in the context of the energy sector.

The method for constructing the observational dataset is a critical limitation. The authors used the YouTube Data API to search for 13 predefined "concepts" (e.g., "renewable energy," "energy geopolitics") and limited the search to Spanish. While practical, this approach fundamentally biases the dataset towards content that explicitly uses these specific, top-level terms. It may miss nuanced or emerging discussions that use different vernacular, and it inherently excludes the broader, less formal ecosystem of content creators (like influencers or hobbyists) who might discuss energy topics without using such "official" terminology. The dat

Attachment Submitted filename: response.pdf

# Peer review: Decision Letter 1

**DOI:** 10.1371/journal.pone.0352691.r003

**Authors:** Cheong Kim, Cheong Kim

15 Jun 2026

An analytical and experimental study of the energy transition discourse on YouTube

PONE-D-25-62607R1

Dear Dr. Vicens,

We’re pleased to inform you that your manuscript has been judged scientifically suitable for publication and will be formally accepted for publication once it meets all outstanding technical requirements.

Within one week, you’ll receive an e-mail detailing the required amendments. When these have been addressed, you’ll receive a formal acceptance letter and your manuscript will be scheduled for publication. Please note that Reviewer #1 suggested a minor change to the Abstract. Please make sure to incorporate this change into the final version during the proofreading stage.

An invoice will be generated when your article is formally accepted. Please note, if your institution has a publishing partnership with PLOS and your article meets the relevant criteria, all or part of your publication costs will be covered. Please make sure your user information is up-to-date by logging into Editorial Manager at Editorial Manager® and clicking the ‘Update My Information' link at the top of the page. For questions related to billing, please contact billing support.

If your institution or institutions have a press office, please notify them about your upcoming paper to help maximize its impact. If they’ll be preparing press materials, please inform our press team as soon as possible -- no later than 48 hours after receiving the formal acceptance. Your manuscript will remain under strict press embargo until 2 pm Eastern Time on the date of publication. For more information, please contact onepress@plos.org.

Kind regards,

Cheong Kim

Academic Editor

PLOS One

Additional Editor Comments (optional):

Reviewers' comments:

Reviewer's Responses to Questions

**Comments to the Author**

1. If the authors have adequately addressed your comments raised in a previous round of review and you feel that this manuscript is now acceptable for publication, you may indicate that here to bypass the “Comments to the Author” section, enter your conflict of interest statement in the “Confidential to Editor” section, and submit your "Accept" recommendation.

Reviewer #1: All comments have been addressed

Reviewer #2: All comments have been addressed

**********

2. Is the manuscript technically sound, and do the data support the conclusions?

The manuscript must describe a technically sound piece of scientific research with data that supports the conclusions. Experiments must have been conducted rigorously, with appropriate controls, replication, and sample sizes. The conclusions must be drawn appropriately based on the data presented.

Reviewer #1: Yes

Reviewer #2: Yes

**********

3. Has the statistical analysis been performed appropriately and rigorously?

Reviewer #1: Yes

Reviewer #2: Yes

**********

4. Have the authors made all data underlying the findings in their manuscript fully available?

The PLOS Data policy requires authors to make all data underlying the findings described in their manuscript fully available without restriction, with rare exception (please refer to the Data Availability Statement in the manuscript PDF file). The data should be provided as part of the manuscript or its supporting information, or deposited to a public repository. For example, in addition to summary statistics, the data points behind means, medians and variance measures should be available. If there are restrictions on publicly sharing data—e.g. participant privacy or use of data from a third party—those must be specified.

Reviewer #1: Yes

Reviewer #2: Yes

**********

5. Is the manuscript presented in an intelligible fashion and written in standard English?

PLOS ONE does not copyedit accepted manuscripts, so the language in submitted articles must be clear, correct, and unambiguous. Any typographical or grammatical errors should be corrected at revision, so please note any specific errors here.

Reviewer #1: Yes

Reviewer #2: Yes

**********

6. Review Comments to the Author

Please use the space provided to explain your answers to the questions above. You may also include additional comments for the author, including concerns about dual publication, research ethics, or publication ethics. (Please upload your review as an attachment if it exceeds 20,000 characters)

Reviewer #1: The revised manuscript demonstrates substantial improvement in clarity, methodological transparency, and scholarly positioning. However, the abstract still needs some improvement in terms of statistical findings instead of a general statement of findings.

"While user retention was higher in the early segments of the conversational content, a higher retention in the mid to late sections of academic videos was found. Data from the Google Ads platform provided a deeper understanding of engagement across user profiles."

Suggestion for improvement:

While user retention was higher in the early segments of the conversational content (time ratio? p value?), a higher retention in the mid to late sections of academic videos was found (time ratio? p value?). Data from the Google Ads platform provided a deeper understanding (p value?) of engagement across user profiles.

Reviewer #2: This article investigates how energy transition topics are communicated on YouTube and how communication style influences audience engagement. The authors combine two complementary approaches: (1) a large-scale observational analysis of more than 2,000 Spanish-language YouTube videos related to energy transition topics, and (2) a controlled experiment using two mirror YouTube channels that present identical content with different levels of linguistic formality. The study employs natural language processing (NLP) techniques, sentiment analysis, topic modelling, and audience engagement metrics obtained from YouTube Analytics and Google Ads.

The research addresses an important and timely issue because social media increasingly shapes public understanding of scientific and policy-related topics. The study aims to contribute to knowledge about science communication, digital engagement, and public discourse surrounding energy transition.

One of the strongest aspects of the paper is its integration of observational and experimental approaches. Most previous studies rely exclusively on observational social media data, limiting causal interpretation. By creating two mirror YouTube channels with different communication styles, the authors move beyond correlation and attempt to assess causal relationships between language formality and audience engagement.

The mirror-channel experiment represents a novel contribution. Few studies in science communication conduct real-world platform experiments. The design increases ecological validity because engagement is measured in an authentic social media environment rather than a laboratory setting.

1. Although several correlations are statistically significant, their practical magnitudes are small. For example, negative sentiment and comments show a weak positive correlation (r = 0.11), while formality and comments show a weak negative correlation (r = −0.11). These effect sizes indicate weak relationships despite statistical significance. The large sample size likely contributed to the detection of statistical significance. The manuscript would benefit from a more explicit discussion of practical significance in addition to statistical significance.

**********

7. PLOS authors have the option to publish the peer review history of their article (what does this mean?). If published, this will include your full peer review and any attached files.

If you choose “no”, your identity will remain anonymous but your review may still be made public.

**Do you want your identity to be public for this peer review?**  For information about this choice, including consent withdrawal, please see our Privacy Policy.

Reviewer #1: No

Reviewer #2: No

**********

# Editor report: Acceptance letter

**DOI:** 10.1371/journal.pone.0352691.r004

**Authors:** Cheong Kim, Cheong Kim

PONE-D-25-62607R1

PLOS One

Dear Dr. Vicens,

I'm pleased to inform you that your manuscript has been deemed suitable for publication in PLOS One. Congratulations! Your manuscript is now being handed over to our production team.

At this stage, our production department will prepare your paper for publication. This includes ensuring the following:

* All references, tables, and figures are properly cited

* All relevant supporting information is included in the manuscript submission,

* There are no issues that prevent the paper from being properly typeset

You will receive further instructions from the production team, including instructions on how to review your proof when it is ready. Please keep in mind that we are working through a large volume of accepted articles, so please give us a few days to review your paper and let you know the next and final steps.

Lastly, if your institution or institutions have a press office, please let them know about your upcoming paper now to help maximize its impact. If they'll be preparing press materials, please inform our press team within the next 48 hours. Your manuscript will remain under strict press embargo until 2 pm Eastern Time on the date of publication. For more information, please contact onepress@plos.org.

You will receive an invoice from PLOS for your publication fee after your manuscript has reached the completed accept phase. If you receive an email requesting payment before acceptance or for any other service, this may be a phishing scheme. Learn how to identify phishing emails and protect your accounts at https://explore.plos.org/phishing.

If we can help with anything else, please email us at customercare@plos.org.

Thank you for submitting your work to PLOS ONE and supporting open access.

Kind regards,

PLOS ONE Editorial Office Staff

on behalf of

Dr. Cheong Kim

Academic Editor

PLOS One
